# Endogenous glutamate determines ferroptosis sensitivity via ADCY10-dependent YAP suppression in lung adenocarcinoma

**DOI:** 10.7150/thno.55482

**Published:** 2021-03-24

**Authors:** Xiao Zhang, Keke Yu, Lifang Ma, Zijun Qian, Xiaoting Tian, Yayou Miao, Yongjie Niu, Xin Xu, Susu Guo, Yueyue Yang, Zhixian Wang, Xiangfei Xue, Chuanjia Gu, Wentao Fang, Jiayuan Sun, Yongchun Yu, Jiayi Wang

**Affiliations:** 1 Department of Thoracic Surgery, Shanghai Chest Hospital, Shanghai Jiao Tong University, Shanghai, 200030, China.; 2 Shanghai Institute of Thoracic Oncology, Shanghai Chest Hospital, Shanghai Jiao Tong University, Shanghai, 200030, China.; 3 Department of Bio-bank, Shanghai Chest Hospital, Shanghai Jiao Tong University, Shanghai, 200030, China.; 4 Shanghai Municipal Hospital of Traditional Chinese Medicine, Shanghai University of Traditional Chinese Medicine, Shanghai, 200071, China.; 5 Department of Clinical Laboratory Medicine, Shanghai Tenth People's Hospital of Tongji University, Shanghai, 200072, China.; 6 Department of Respiratory Endoscopy, Shanghai Chest Hospital, Shanghai Jiao Tong University, Shanghai, 200030, China.; 7 Department of Respiratory and Critical Care Medicine, Shanghai Chest Hospital, Shanghai Jiao Tong University, Shanghai 200030, China.

**Keywords:** XBP1 splicing, Ferritin, Hippo pathway, GFPT1, HBP-dependent O-GlcNAcylation, NCOA4

## Abstract

**Rationale:** Ferroptosis, a newly identified form of regulated cell death, can be induced following the inhibition of cystine-glutamate antiporter system X_C_^-^ because of the impaired uptake of cystine. However, the outcome following the accumulation of endogenous glutamate in lung adenocarcinoma (LUAD) has not yet been determined. Yes-associated protein (YAP) is sustained by the hexosamine biosynthesis pathway (HBP)-dependent O-linked beta-N-acetylglucosaminylation (O-GlcNAcylation), and glutamine-fructose-6-phosphate transaminase (GFPT1), the rate-limiting enzyme of the HBP, can be phosphorylated and inhibited by adenylyl cyclase (ADCY)-mediated activation of protein kinase A (PKA). However, whether accumulated endogenous glutamate determines ferroptosis sensitivity by influencing the ADCY/PKA/HBP/YAP axis in LUAD cells is not understood.

**Methods:** Cell viability, cell death and the generation of lipid reactive oxygen species (ROS) and malondialdehyde (MDA) were measured to evaluate the responses to the induction of ferroptosis following the inhibition of system X_C_^-^. Tandem mass tags (TMTs) were employed to explore potential factors critical for the ferroptosis sensitivity of LUAD cells. Immunoblotting (IB) and quantitative RT-PCR (qPCR) were used to analyze protein and mRNA expression. Co-immunoprecipitation (co-IP) assays were performed to identify protein-protein interactions and posttranslational modifications. Metabolite levels were measured using the appropriate kits. Transcriptional regulation was evaluated using a luciferase reporter assay, chromatin immunoprecipitation (ChIP), and electrophoretic mobility shift assay (EMSA). Drug administration and limiting dilution cell transplantation were performed with cell-derived xenograft (CDX) and patient-derived xenograft (PDX) mouse models. The associations among clinical outcome, drug efficacy and ADCY10 expression were determined based on data from patients who underwent curative surgery and evaluated with patient-derived primary LUAD cells and tissues.

**Results:** The accumulation of endogenous glutamate following system X_C_^-^ inhibition has been shown to determine ferroptosis sensitivity by suppressing YAP in LUAD cells. YAP O-GlcNAcylation and expression cannot be sustained in LUAD cells upon impairment of GFPT1. Thus, Hippo pathway-like phosphorylation and ubiquitination of YAP are enhanced. ADCY10 acts as a key downstream target and diversifies the effects of glutamate on the PKA-dependent suppression of GFPT1. We also discovered that the protumorigenic and proferroptotic effects of ADCY10 are mediated separately. Advanced-stage LUADs with high ADCY10 expression are sensitive to ferroptosis. Moreover, LUAD cells with acquired therapy resistance are also prone to higher ADCY10 expression and are more likely to respond to ferroptosis. Finally, a varying degree of secondary labile iron increase is caused by the failure to sustain YAP-stimulated transcriptional compensation for ferritin at later stages further explains why ferroptosis sensitivity varies among LUAD cells.

**Conclusions:** Endogenous glutamate is critical for ferroptosis sensitivity following the inhibition of system X_C_^-^ in LUAD cells, and ferroptosis-based treatment is a good choice for LUAD patients with later-stage and/or therapy-resistant tumors.

## Introduction

In 2012, Dixon et al. first described ferroptosis, a type of regulated cell death that differs from apoptosis and necroptosis 1. Ferroptosis is characterized by excessive oxidative modification of phospholipids via an iron-dependent mechanism 1, 2. While peroxidation of polyunsaturated fatty acids, including arachidonic acid (AA) and adrenic acid (AdA), is susceptible to ferroptosis 3, iron is essential for this process 4. Iron homeostasis is maintained by cargo proteins such as ferritin, which directly binds iron and protects cells from iron-induced stress 5. Notably, the selective autophagy of ferritin, referred to as ferritinophagy, leads to the release of free iron and increases ferroptosis sensitivity 6, 7.

One of the many ways to elicit ferroptosis is by inhibiting system X_C_^-^ 1. Sorafenib, erastin and its derivatives are potent ferroptotic agonists 8. These molecules block cystine from entering the cell and thus decrease glutathione (GSH) production 9. Because system X_C_^-^ is a 1:1 cystine:glutamate exchange antiporter, a concurrent inhibitory effect on cystine uptake reduces endogenous glutamate efflux 10. To date, many studies have shown the critical roles of cystine depletion 2, 11, but little is known about the outcome following the accumulation of endogenous glutamate. Here, we focus on the so-called intracellular “endogenous glutamate” to distinguish it from externally added glutamate, which can also inhibit system X_C_^-^ by disrupting the concentration gradient.

Lung cancer is one of the leading causes of cancer-related death worldwide, with lung adenocarcinoma (LUAD) being the most prevalent subtype 12. While surgical resection is the most effective therapy for early-stage LUAD 13, advanced-stage patients may benefit from adjuvant cytotoxic therapy 14. However, therapy resistance is often the major cause of LUAD recurrence 15. Recently, tumor cells exhibiting Cisplatin resistance were shown to be sensitive to ferroptosis 16. Furthermore, ferroptosis induction is more pronounced in certain types of tumor cells than in normal cells 11, 16, 17. However, ferroptosis sensitivity may vary among LUAD cells, similar to its variations among types of cancer, and the mechanism underlying the ferroptosis sensitivity of LUAD cells remains unclear.

Yes-associated protein (YAP) functions as a proto-oncoprotein in several malignancies, including LUAD 18, 19. However, YAP can be inhibited after phosphorylation by the tumor-suppressing Hippo pathway 18. Recently, O-linked beta-N-acetylglucosaminylation (O-GlcNAcylation) has been shown to suppress YAP phosphorylation, thus highlighting a novel mechanism to protect YAP against the Hippo pathway activity 20. Uridine-diphosphate N-acetylglucosamine (UDP-GlcNAc), a donor for O-GlcNAcylation, is synthesized from glucose through the hexosamine biosynthetic pathway (HBP), in which glutamine-fructose-6-phosphate transaminase (GFPT1) is the rate-limiting enzyme 21-23. Prior studies have shown that GFPT1 is phosphorylated and inhibited by cAMP-dependent protein kinase A (PKA) 24, and the major source of cAMP is ATP, which can be converted by the members of the adenylate cyclase (ADCY) family 25, 26. In humans, there are nine transmembrane ADCYs, i.e., ADCY1-9 and one soluble ADCY enzyme, i.e., ADCY10 27. Although enhanced tumorigenesis is a result of elevated O-GlcNAcylation and YAP, a very recent study showed that cancer cells with a higher YAP expression are more sensitive to ferroptosis 17. Although the accumulation of endogenous glutamate is accompanied by impaired cystine uptake following the inhibition of system X_C_^-^ and YAP is associated with ferroptosis; to the best of our knowledge, no study has linked system X_C_^-^ and YAP to the determination of the ferroptosis sensitivity in LUAD cells.

Therefore, we investigated whether and how the accumulation of endogenous glutamate following system X_C_^-^ inhibition determines ferroptosis sensitivity by influencing the ADCY/PKA/HBP/YAP axis in LUAD cells. GFPT1 at low levels is unable to preserve YAP O-GlcNAcylation upon the accumulation of glutamate, thus enhancing YAP phosphorylation and degradation. We also discovered that the concentration of ADCY10 correlates with the degree of glutamate-induced PKA-dependent GFPT1 inhibition; thus is the most important upstream factor in determining ferroptosis sensitivity. Clinically, we provide further evidence showing that ferroptosis-based treatment is a good choice for LUAD patients with higher ADCY10 levels.

## Materials and Methods

### Cell culture

Established MRC-5, WI38, BEAS-2B, H358, H1650, PC9, H1975, A549, H1299 and HCC827 cell lines were purchased from Fuheng Biotechnology (Shanghai, China). All the cell lines were validated by short tandem repeat analysis (Supplementary Document). H1975- and HCC827-based AZD9291 resistant (H1975-A^Res^ and HCC827-A^Res^) cell lines were gifts from Dr. Tianxiang Chen (Shanghai Lung Cancer Center, Shanghai Chest Hospital, Shanghai Jiao Tong University). A549-based Cisplatin resistant (A549-C^Res^) cell lines were purchased from Fuheng Biotechnology. Patient-derived primary LUAD cells were established from LUAD tissues. Briefly, the tissues in a size less than 1.0 cm^3^ without necrosis were immediately washed with ice-cold Dulbecco's phosphate buffered saline (DPBS) for 3 times before re-suspending in Dulbecco's modified eagle medium (DMEM) containing collagenase I (2 mg/ml, Solarbio, Shanghai, China) at 37 °C for 4 h. After washing for 3 additional times with DMEM, cells were cultured in routine conditions. For monolayer culture, cells were cultured with DMEM supplemented with 10% FBS and 1% penicillin/streptomycin. For 3D spheroid culture, basement membrane extract (BME) (Trevigen, Gaithersburg, MD, USA) was seeded in a 96-well plate at 50 μl/well and pre-warmed at 37 °C for 0.5 h. Subsequently, cells were seeded on top of the plate coated with BME at a density of 1×10^5^ cells per well. Images were captured using microscope after 7-day-culture, and the relative size and numbers of spheroids were calculated. For analysis following cystine deprivation, cells were cultured in DMEM without cystine. For hypoxia condition, cells were maintained in an anaerobic chamber with 0.1% O_2_. For glucose deprivation, cells were cultured in DMEM without glucose.

### Mouse experiments and tissue samples

For generation of cell-derived tumor xenograft (CDX) mouse models, established LUAD cells (initial 5×10^6^) were subcutaneously injected into 6-week-old athymic nude mice (Jiesijie, Shanghai, China). For generation of patient-derived tumor xenograft (PDX) mouse models, fresh LUAD specimens in a size of 2-3 mm^3^ were implanted into six-week-old athymic nude mice. The 3^rd^ generations of PDX-bearing mice were used for drug administration and survival study. For intrapulmonary tumor model, 6-week-old nude mice were intrapleurally injected with LUAD cells (5×10^6^) under anesthesia. For limiting dilution cell transplantation assays, a serial dilution of H1975 cells were injected into 6-week-old athymic nude mice and the tumor incidence was reported 12 weeks after transplantation. For drug administration experiments, mice bearing obvious tumors were subcutaneously injected by imidazole ketone erastin (IKE, 50 mg/kg) with or without liproxstatin-1 (Lipro-1, 10 mg/kg). The tumor volume was calculated as 0.5 × L × W^2^ (L indicating length while W indicating width). Tumorous and adjacent lung tissues of patients (mean age ± SD, 64.62 ± 10.02 years; male: female ratio, 1.15:1) were recruited in Shanghai Chest Hospital (Shanghai, China) from May 2013 to March 2019. Informed written consents were obtained from all patients. The study (including animal experiments) was approved by the institutional ethics committee of Shanghai Chest Hospital.

### Reagents and plasmids

For reagents, erastin (Sigma, St Louis, MO, USA), ferrostatin-1 (Fer-1, Sigma), deferoxamine (DFO, sigma), Sorafenib (Selleck, Houston, TX, USA), RSL3 (Sigma), doxycycline (Dox, Clontech, Mountain View, CA, USA), epigallocatechol gallate (EGCG, MedChemExpress, Monmouth Junction, NJ, USA), glucose (Sigma), glucosamine (GlcN, MedChemExpress), N-acetylglucosamine (GlcNAc, Sigma), cyclohexamide (CHX, Sigma), H89 (MedChemExpress), Rp-Adenosine 3',5'-cyclic monophosphorothioate (Rp-cAMPS, MedChemExpress), PKA inhibitor (PKI, MedChemExpress), β-mercaptoethanol (β-ME, Sigma), N-Acetyl-L-cysteine (NAC, Sigma), GSH (Sigma), aminooxyacetic acid (AOA, MedChemExpress), dimethyl alpha ketoglutarate (DMK, Sigma), ethylenebis (oxyethylenenitrilo) tetraacetic acid (EGTA, MedChemExpress), 8-(4-Chlorophenylthio)-2'-O-methyladenosine 3',5'-cyclic monophosphate (8-pCPT-cAMP, Sigma), N^6^-Benzoyladenosine-3',5'-cyclic monophosphate (6-Bnz, Sigma), KH7 (Sigma), Gemcitabine (MedChemExpress), Paclitaxel (MedChemExpress), Cisplatin (MedChemExpress) and AZD9291 (MedChemExpress) were used for cell treatments.

For plasmids, wild type (WT)-YAP-FLAG, T241A-YAP-FLAG, transcriptional factor CP2 (TFCP2) luciferase (LUC) #1-#3 and TEA domain family member (TEAD) LUC reporters were acquired from our previous studies 20, 28. LentiCRISPR v2 based constructs were used for knockout of beta-transducin repeat containing E3 ubiquitin protein ligase (βTrCP), SMAD specific E3 ubiquitin protein ligase 1 (Smurf1), solute carrier family 7 member 11 (SLC7A11), solute carrier family 3 members (SLC3A2), GFPT1 and ADCY1-10. Inducible glutamate dehydrogenase 1 (iGLUD1) expressing plasmid was constructed using pLVX-Tet-on-Puro. Inducible shRNA targeting glutamate and aspartate transporter (ishGLAST) and glutamate transporter-1 (ishGLT1) were constructed using Tet-pLKO-neo. shRNAs targeting against nuclear receptor coactivator 4 (NCOA4), X-box binding protein 1 (XBP1) and plasmids expressing glutamic-oxaloacetic transaminase 2 (GOT2), glutamic-pyruvic transaminase 2 (GPT2), WT-GFPT1-FLAG, PKACα, ADCY10 and spliced form of X-Box binding protein 1 (XBP1s) were purchased from Biolink LTD (Shanghai, China). ADCY10 expressing plasmid was purchased from Vigene Biosciences (Shanghai, China). siRNA targeting against ferritin heavy chain 1 (FTH1) was purchased from Shanghai GenePharma Co., Ltd (Shanghai, China). *GFPT1 and FTH1*-promoter luciferase reporter was constructed using pGL4.21 vector. S205A-, S205E-, S235A-, S243A-GFPT1-FLAG were constructed using overlapping PCR and cloned into pcDNA3.1(+) vector. The primers, gRNAs and siRNAs were summarized in Table S1.

### Immunofluorescence (IF), immunohistochemistry (IHC), immunoblotting (IB) and enzyme linked immunosorbent assay (ELISA)

IF and IHC were performed according to the conventional protocols. The primary antibodies used for IF were: anti-YAP (Abcam, Hong Kong, China, #ab52771), anti-human vimentin (hVIM, Raybiotech, Peachtree Corners, GA, USA, #128-10172-1), anti-ADCY10 (Abcam, #ab203204) and anti-cluster of differentiation 31 (CD31) (Abcam, #ab9498). The primary antibodies used for IHC were: anti-YAP (Abcam, #ab52771) and anti-ADCY10 (Abcam, #ab203204). For IB, nuclear and cytosol fractions of cells were prepared using a kit from Active Motif (Carlsbad, CA, USA). The proteins were resolved on SDS-PAGE gels with or without phostag™ reagents (Dako, Kyoto, Japan) prior to the standard protocol. The primary antibodies used for IB were: anti-YAP (Abcam, #ab52771 or Santa Cruz Biotechnology, Santa Cruz, CA, USA, #sc-101199), anti-glyceraldehyde-3-phosphate dehydrogenase (GAPDH, Cell Signaling Technology (CST), Boston, MA, USA, #5174 or #51332)), anti-phosphorylated YAP at serine 127 (p-YAP^S127^, Abcam, #ab76252), anti-O-GlcNAcylated YAP at threonine 241 (O-YAP^T241^, developed by Biolynx, Hangzhou, China, 20), anti-O-GlcNAc (Abcam, #ab2739), anti-FLAG (CST, #8146 or #2368), anti-vitronectin (VTN, Abcam, #ab45139), anti-four and a half LIM domains 2 (FHL2, Abcam, #ab202584), anti-glutaminase (GLS, Abcam, #ab156876), anti-alubumin (ALB, Abcam, #ab207327), anti-hemoglobin subunit alpha 1 (HBA1, Abcam, #ab77125), anti-metallothionein 2A (MT2A, Abcam, #ab12228), anti-keratin 81 (KRT81, Abcam, #ab55407), anti-hemoglobin subunit delta (HBD, Abcam, #ab131225), anti-carbonic anhydrase 12 (CA12, Abcam, #ab195233), anti-GFPT1 (Abcam, #ab125069), anti- inositol-requiring protein 1alpha (IRE1α, Abcam, #ab37073), anti-p-IRE1α (Abcam, #ab243665), anti-XBP1s (CST, #40435), anti-LaminB (Abcam, #ab16048), anti-βtubulin (Abcam, #ab18207), anti-ADCY10 (Abcam, #ab203204), anti-phosphorylated GFPT1 at Ser205 (p-GFPT1^S205^, developed by Biolynx), anti-FTH1 (Abcam, #ab65080), anti-βTrCP (Abcam, #ab71753 or #ab233638), anti-ubiquitin, (Ub, Abcam, #ab7780), anti-lysine 48-linked polyubiquitin chain (K48-Ub, CST, #12805), anti-GOT2 (Proteintech, Rosemont, IL, USA, #14800-1-AP), anti-GPT2 (Proteintech, #16757-1-AP), anti-GLAST (Abcam, #ab181036), anti-GLT1 (Abcam, #ab205248), anti-GLUD1 (Abcam, #ab168352), anti-XBP1 (Abcam, #ab37152), anti-PKACα (Abcam, #ab32376), anti-ADCY1 (Abcam, #ab69597), anti-ADCY2 (Abcam, #ab151470), anti-ADCY3 (Abcam, #ab125093), anti-ADCY4 (Abcam, #ab230192), anti-ADCY5 (Abcam, #ab66037), anti-ADCY6 (Abcam, #ab14781), anti-ADCY7 (Abcam, #ab14782), anti-ADCY8 (Abcam, #ab196686), anti-ADCY9 (Abcam, #ab191423), anti-epithelial cadherin (E-cadherin, Abcam, ab231303), anti-neural cadherin (N-cadherin, Abcam, #ab18203), anti-Snail (Abcam, ab216347), anti-Vimentin (Abcam, #ab92547), anti-ferritin light chain 1 (FTL1, Abcam, #ab69090), anti-transferrin receptor (TFRC, Abcam, #ab214039), anti- transferrin (TF, Abcam, #ab109503), anti-iron responsive element binding protein 2 (IREB2, Abcam, #ab232994), anti-CDGSH iron sulfur domain 1 (CISD1, Abcam, ab133970), anti-NCOA4 (Abcam, #ab86707), anti-ferroportin-1 (FPN1, Abcam, #ab239511), anti- heat shock protein family B (small) member 1 (HSPB1, Abcam, #ab109376), anti- nuclear factor, erythroid 2 like 2 (NRF2, Abcam, #ab62352), anti-phosphorylase kinase catalytic subunit gamma 2 (PHKG2, Abcam, #ab167424) and anti-divalent metal transporter 1 (DMT1, Abcam, #ab55812). For ELISA, YAP and ADCY10 levels in tissues were measured using kits from Lichen Biotech Ltd. (Shanghai, China).

### Co-immunoprecipitation (co-IP) and tandem mass tags (TMT) analysis

For co-IP, whole cell lysates (WCL) were incubated with antibodies-conjugated protein A/G magnetic beads (Novex, Oslo, Norway) in Western/IP lysis buffer (Beyotime, Haimen, China) at 4 °C overnight. Immunoprecipitates were washed five times by Western/IP lysis buffer before subjection to IB. The antibodies used for co-IP were: anti-FLAG (CST, #8146 or #2368), anti-YAP (Santa Cruz Biotechnology, #sc-101199), anti-βTrCP (Abcam, #ab233638) and anti-IgG (CST, #3900). TMT was performed and analyzed by Luming Biotechnology (Shanghai, China). A fold change (FC) > 2 or < 0.5 with a P < 0.05 was considered as significance for differential expression.

### Measurements of metabolites

AA was measured using a kit from CUSABIO (Houston, TX, USA). Labile iron, malondialdehyde (MDA), Ca^2+^ and cyclic adenosine monophosphate (cAMP) were measured using the kits from Abcam. Glucose, fructose-6-phosphate (F-6-P), GSH, glutamine and glutamate were assessed using the kits from Sigma. AdA, Glucosamine-6-phosphate (GlcN-6-P), UDP-N-acetylglucosamine (UDP-GlcNAc), N-acetylglucosamine-6-phosphate (GlcNAc-6-P) and N-acetylglucosamine-1-phosphate (GlcNAc-1-P) were measured using the kits from Lichen Biotech Ltd. (Shanghai, China). All the measurements were carried out in strict accordance with the instructions provided by the manufacturer.

### Quantitative RT-PCR (qPCR)

Total RNA was extracted using Trizol (Ambion, Carlsbad, CA, USA) and reverse-transcribed into complementary DNA using the PrimeScript^TM^ RT reagent kit (Takara, Dalian, China). For real-time qPCR, the SYBR premix Ex Taq (Takara) kit was used for the detection of *YAP*, *ADCY10* and *FTH1* mRNAs. For the evaluation of *XBP1* splicing, semi-qPCR was performed to examine the *XBP1s* and total *XBP1* mRNA. The PCR was terminated at the cycle 29 and the products were visualized by agarose gel electrophoresis. The primers are listed in Table S1.

### Luciferase reporter assay

Firefly luciferase reporter plasmids for the detection of *GFPT1* and* FTH1* promoter activity, TFCP2- and TEAD-based transcription activities were co-transfected with renilla luciferase reporter plasmids. Twenty-four hours after transfection, the ratios between firefly and renilla luciferase activities were measured using a Dual-luciferase® reporter assay system (Promega, Madison, WI, USA).

### Chromatin immunoprecipitation (ChIP) and Re-ChIP

ChIP and Re-ChIP experiments were performed using the kits from Active Motif (Carlsbad, CA, USA). For ChIP, cells (2×10^7^) were fixed using 1% formaldehyde, washed with PBS and lysed using lysis buffer. After sonication, protein-DNA complexes were incubated overnight with antibody-coupled protein G beads at 4 °C. In the second day, DNA was eluted in 1% SDS/0.1M NaHCO_3_, reversed cross-link at 65 °C, purified via phenol/chloroform extraction and ethanol precipitation, and subjected to qPCR. For Re-ChIP experiments, the complexes pulled down by the ChIP using the antibodies specific for the first protein were eluted by incubation 30 min at 37 °C in 10 mM DTT. After centrifugation, the supernatant was diluted 20 times with Re-ChIP buffer (1% Triton X-100, 2 mM EDTA, 150 mM NaCl, 20 mM Tris-HCl, pH 8.1), and subjected again into the ChIP procedure with the antibodies targeting against the second protein. The primary antibodies used in ChIP and Re-ChIP experiments were: anti-YAP (CST, #14074), anti-TFCP2 (CST, #80784), and anti-IgG (CST, #3900).

### Measurements of cell viability, cell death and Lipid ROS generation

Cell viability was measured using a CellTiter-Glo luminescent cell viability assay (Promega) according to the manufacturer's instructions. Cell death was analyzed by staining with SYTOX Green (Invitrogen, Carbsland, CA, USA) followed by flow cytometry. Lipid ROS generation was measured by adding C11-BODIPY (Invitrogen) to a final concentration of 1.5 μM for 20 min before cell harvest. Lipid ROS positive cells were finally assessed by a flow cytometer.

### Electron microscopic analysis (EM)

To observe the morphological change of mitochondria following induction of ferroptosis, cells were seeded onto 4-well chambered cover glass (Thermo Scientific, #155382) at a density of 15,000 cells/well and treated with or without erastin for 24 h. Images were captured using the Olympus EM208S transmission electron microscope (HITACHI, Tokyo, Japan).

### Anchorage-independent colony formation assay

LUAD cells were seeded in a 6-well plate containing 0.3% agarose in DMEM at a density of 6×10^3^ cells per well. Two weeks later, the size and numbers of colonies were calculated under microscope.

### *Ex vivo* tumor slice culture

Fresh LUAD tissues were perfused with 2% low melting point agarose (Sigma). The tumors were cooled, excised, placed in ice-cold Hank's balanced salt solution (HBSS), and cut with a tissue slicer. Slices were incubated in DMEM containing penicillin and streptomycin at 37 °C, 5% CO_2_ for 4 h, and the medium was replaced three times to remove excess agarose. Subsequently, tissue slice was treated with erastin with or without Fer-1 for 24 h before Propidium Iodide (PI) staining.

### Electrophoretic mobility shift assay (EMSA)

EMSA was performed as described in the previous study 29. The light shift kit (Pierce, Rockford, IL, USA) was used. Nuclear extracted proteins were prepared using the kit from Active Motif (Carlsbad, CA, USA) and incubated in the reaction buffer on ice followed by the addition of the biotin-labeled probes (synthesized and 5' labeled by Sangon Inc., Shanghai, China). For supershift assays, antibodies against TFCP2 (CST, #80784) or IgG (CST, #3900) were added to the mixture before adding the probe. The probes used are listed in Table S1.

### GFPT1 activity assay

The total cellular enzymatic activity of GFPT1 was assayed using the glutamate dehydrogenase (GDH) method. Briefly, cells were lysed in CHAPS lysis buffer (Sigma). GFPT buffer (50 mM Tris, pH 7.8, 5 mM EDTA, 5 mM GSH, 5 mM glucose-6-P, 50 mM KCl) and GDH reaction buffer (6 mM glutamine, 0.8 mM fructose-6-phosphate, 0.3 mM APAD, 50 mM KCl, 0.1 mM KH_2_PO_4_, 6 U of glutamate dehydrogenase (Sigma)) were added into cell lysate in a 96-well plate and incubated for 37 °C for 90 min. Finally, OD370 was measured to estimate GFPT1 activity in cell lysates.

### Statistical analysis

Tests used to examine the differences between groups were student's t test, one-way, two-way ANOVA, χ^2^ test and the Spearman rank-correlation analysis. A P < 0.05 was considered statistically significant.

### Data availability

TMT data have been deposited in ProteomeXchange Consortium (accession number: PXD020205). The username for reviewing TMT data is reviewer51782@ebi.ac.uk, and the password is cjPsijRs.

## Results

### The degree to which YAP suppressed is critical to the ferroptosis sensitivity of LUAD cells

**Figure 1:**
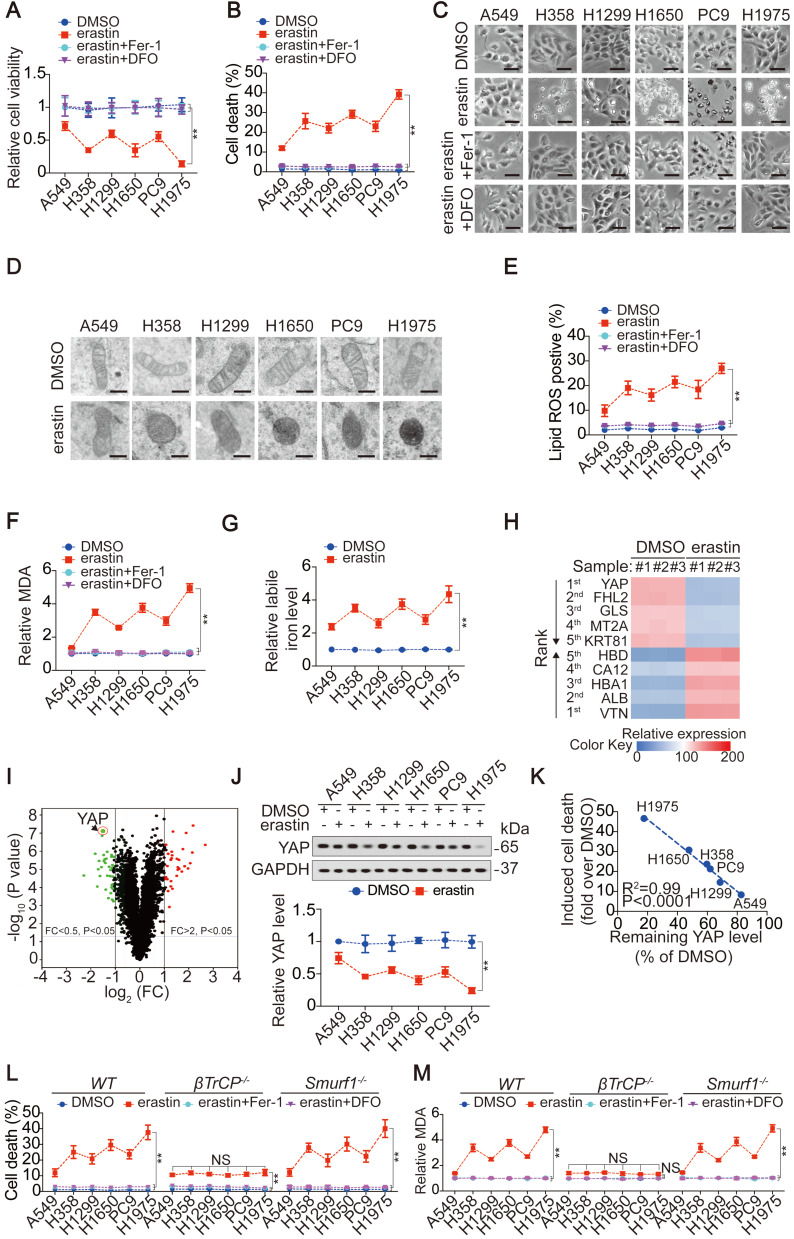
** The level YAP suppressed contributes to ferroptosis sensitivity in LUAD cells. (A-B)** Cell viability, and cell death were assayed by measuring cellular ATP (A), and SYTOX green staining followed by flow cytometry (B) in LUAD cells after treating with erastin (10 µM) with or without Fer-1 (1 µM) or DFO (80 µM) for 24 h. **(C)** Cell morphology was monitored by phase-contract microscopy in LUAD cells under the same treatment as that in panel A for 12 h. **(D)** Representative TEM images for LUAD cells following treating with or without erastin (10 µM) for 12 h. Scale bar, 500 nm. **(E-F)** Generation of lipid ROS and MDA were assayed by measuring C11BODIPY staining followed by flow cytometry (E) and an MDA testing kit (F) in LUAD cells under the same treatment as that in panel A for 16 h. **(G)** Relative labile iron was measured in LUAD cells after treating with or without erastin (10 µM) for 24 h. (H-I) Heatmap showing the first five ranked upregulated and downregulated proteins in H1975 cells, as measured by TMT, after treating with erastin (10 µM) for 24 h (H). Volcano plot was also used to display altered proteins (I). **(J)** IB of YAP in LUAD cells treated with DMSO or erastin (10 µM) for 24 h. The level of YAP was normalized to that of GAPDH, and the normalized level of YAP in DMSO-treated A549 cells was arbitrarily set to 1. **(K)** Correlation between induced cell death and remaining YAP level after erastin (10 µM) treatment for 24 h. Induced cell death and remaining YAP level was calculated as fold or percentage to the ones treated with DMSO. **(L-M)** Cell death (L) and MDA (M) were measured in *WT*, *βTrCP^-/-^* and *Smurf1^-/-^* H1975 cells after treating with erastin (10 µM) with or without Fer-1 (1 µM) or DFO (80 µM) for 24 h. The data are shown as the mean ± SD from three biological replicates (including IB). **P < 0.01 indicates statistical significance. Data in A, B, E, F, G, J, L, M were analyzed using a two-way ANOVA test. Data in H and I were analyzed using a Student's t test. Data in K were analyzed using the Spearman rank-correlation analysis.

First, we investigated whether LUAD cells are more sensitive to ferroptosis than lung fibroblast cells. Compared to those in LUAD cells, lower levels of labile iron, AA and AdA were found in lung fibroblast cells, resulting in resistance to erastin-induced ferroptosis (Figure S1A-D). However, LUAD cells were sensitive to erastin, although the degrees of reduced-cell viability (Figure 1A), induced-cell death (Figure 1B-C), -smaller than normal mitochondria with condensed mitochondrial membrane densities (Figure 1D), -lipid ROS and MDA generation (Figure 1E-F) and -labile iron elevation (Figure 1G), varied. Reasoning that the effects induced by erastin could be reversed by Fer-1, a lipophilic radical scavenger and DFO, a redox-inactive iron chelator (Figure 1A-C, E-F), they were ferroptosis-associated. These data demonstrated that ferroptosis can be induced but the ferroptosis sensitivities in LUAD cells were varied.

To investigate the mechanism that determines the sensitivity of LUAD cells to ferroptosis, we performed proteomics analysis of H1975 cells before and after treatment with erastin. The previously reported ferroptosis-associated genes HBA1 30, solute carrier family 1 member 4 (SLC1A4) 31 and prostaglandin-endoperoxide synthase 2 (PTGS2) 32 were also found to be upregulated by erastin (Figure S1E), verifying the reliability of the proteomics analysis. Because H1975 and A549 cells showed the highest and lowest sensitivity to erastin (Figure 1A-G), respectively, we compared the alterations of the top 5 ranked downregulated and upregulated proteomics-predicated proteins (Figure 1H-I) between H1975 and A549 cells. In A549 cells, all 10 proteins were altered in the same direction as those in the H1975 cells. However, in contrast to the other 9 candidates, erastin suppression of YAP in A549 cells was not as pronounced as that in H1975 cells (Figure S1F). A comparison of xenografts generated by H1975 and A549 cells further indicated that the greater the suppression of YAP, the greater the inhibition of ferroptosis-associated tumor growth and elevation of MDA level (Figure S1G-H).

Similar to erastin, Sorafenib is also an inhibitor of system X_C_^-^ and has the capacity to induce ferroptosis and reduce YAP levels in LUAD cells (Figure 1J and Figure S1I-J). However, RSL3, another stimulator of ferroptosis targeting glutathione peroxidase 4 (GPX4), failed to induce ferroptosis or reduce YAP levels (Figure S1I and K), indicating that YAP is suppressed in LUAD cells specifically following system X_C_^-^ inhibition. The finding that erastin did not suppress YAP at the mRNA level (Figure S1L) led us to investigate whether erastin regulates YAP at the protein level. Indeed, treatment with erastin caused a Hippo pathway-like nuclear separation of YAP in H1975 cells (Figure S1M). Treatment with erastin or Sorafenib but not RSL3 also induced K48-linked polyubiquitination of YAP by recruiting the well-established YAP-specific ubiquitin E3 ligase (E3) βTrCP (Figure S1N-O). Deletion of βTrCP but not Smurf1, a YAP-unrelated E3, blocked erastin suppression of YAP in all the tested LUAD cells (Figure S1P-Q), implying that βTrCP is the key to decreasing YAP following system X_C_^-^ inhibition.

Subsequently, we investigated whether the degree to which YAP is suppressed is critical to ferroptosis sensitivity. The degree of induced cell death was negatively correlated with the level of YAP remaining in LUAD cells (Figure 1J-K). Blocking erastin suppression of YAP by βTrCP deletion led to robust reduction in ferroptosis and MDA, which were nearly at the same levels among LUAD cells (Figure 1L-M), suggesting that ferroptosis sensitivity was diminished. However, deletion of Smurf1 did not have a similar effect (Figure 1L-M). Moreover, we excluded the possible impact of βTrCP deletion on cell proliferation during the timeframe of our experiments (Figure S1R), thus further indicating that the βTrCP deletion solely affect ferroptosis.

### Ferroptosis sensitivity of LUAD cells is determined by endogenous glutamate-dependent suppression of YAP O-GlcNAcylation

Inhibiting system X_C_^-^ simultaneously suppresses cystine uptake and glutamate efflux 1, 33. To provide direct evidence to support the hypothesis that the accumulation of endogenous glutamate is essential for ferroptosis sensitivity of LUAD cells, cystine was depleted to mimic the impaired cystine uptake, and glutamate was added to reach an intracellular concentration similar to that following the inhibition of system X_C_^-^ (Figure S2A-B). Cystine deprivation induced ferroptosis (Figure S2C), a finding consistent with that of other studies 17, 34; however, the degree of ferroptosis did not vary among LUAD cells until glutamate levels were forcibly elevated (Figure S2C), further demonstrating that the shortage of cystine is the key to trigger ferroptosis and glutamate accumulation is essential to determine ferroptosis sensitivity following the inhibition of system X_C_^-^.

**Figure 2:**
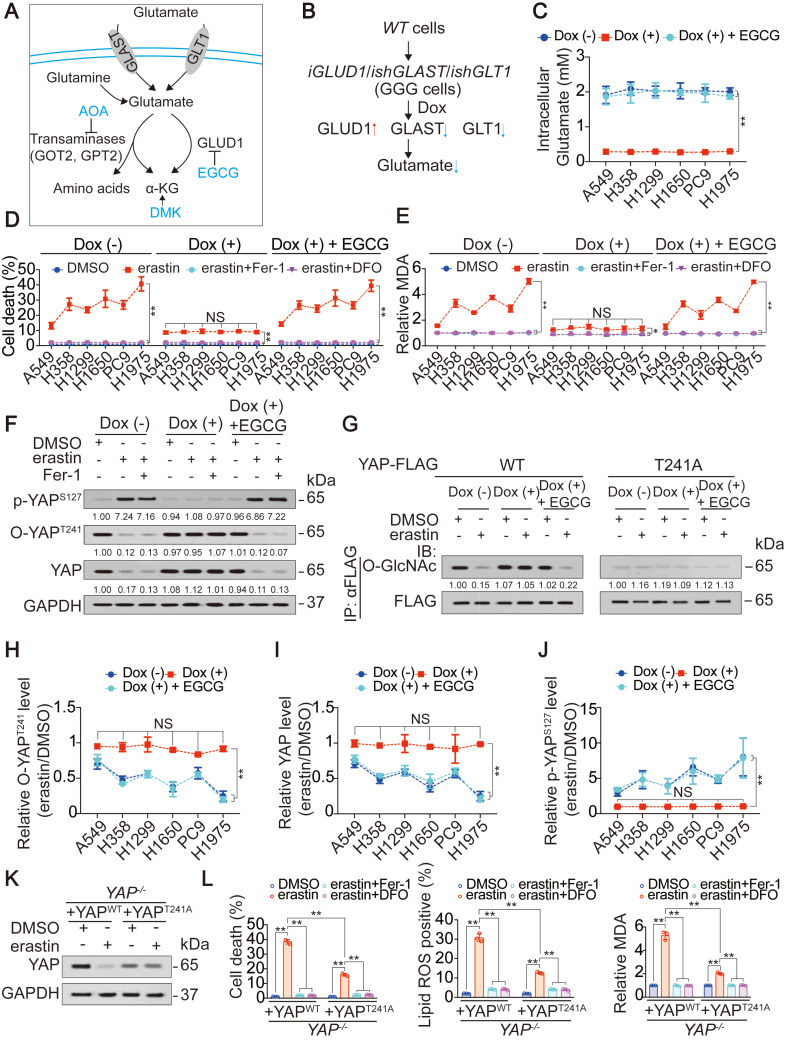
** Accumulation of endogenous glutamate determines ferroptosis sensitivity via decreasing YAP O-GlcNAcylation. (A)** Schematic overview of the glutamate uptake and metabolism. **(B)** Schematic representation of the construction of GGG cells. **(C)** Intracellular glutamate was measured in GGG cells after treating with or without Dox (1 µg/ml), in the presence or absence of EGCG (5 µM) for 24 h. **(D-E)** Endogenous glutamate contributes to ferroptosis sensitivity. GGG cells were pretreated with or without Dox (1 µg/ml) and EGCG (5 µM) for 24 h before further treating with erastin (10 µM) with or without Fer-1 (1 µM) or DFO (80 µM). Cell death was measured after treatment for 24 h by SYTOX green staining followed by flow cytometry (D). Relative MDA was measured after treatment for 16 h (E). **(F)** Endogenous glutamate modulates phosphorylation and O-GlcNAcylation of YAP. Phosphorylation of YAP at S127, O-GlcNAcylation of YAP at T241 and total YAP were measured by IB in H1975-based GGG cells treated the same way as that in panel D, but treated with erastin for 8 h. The level of proteins was normalized to that of GAPDH, and the normalized level of proteins in DMSO-treated cells was arbitrarily set to 1. **(G)** Thr241 is the major O-GlcNAcylation site of YAP to be regulated by endogenous glutamate. Exogenous YAP-FLAG, either WT or T241A was transfected into H1975-based GGG cells before treating the same way as that in panel D, but treated with erastin for 8 h. Immunoprecipitation of YAP-FLAG was performed using anti-FLAG antibodies, and YAP O-GlcNAcylation was measured by IB using anti-O-GlcNAc antibodies. The level of proteins was normalized to that of FLAG, and the normalized level of proteins in DMSO-treated cells was arbitrarily set to 1. **(H-J)** Endogenous glutamate is critical for YAP modification and regulation. The relative O-YAP^T241^ (H), total YAP level (I) and p-YAP^S127^ (J) were measured by IB and calculated between those treated with erastin (10 µM) and DMSO in GGG cells treated the same way as that in panel F. **(K-L)** Thr241 O-GlcNAcylation of YAP is critical for ferroptosis sensitivity. YAP level (K), cell death, lipid ROS and MDA generations (L) were measured in *YAP^-/-^* H1975 cells reconstituting with YAP^WT^ or YAP^T241A^ before further treating with erastin (10 µM) with or without Fer-1 (1 µM) or DFO (80 µM). YAP level, cell death and lipid ROS and MDA generations were measured after treatment for 8 h, 24 h and 16 h, respectively. The data are shown as the mean ± SD from three biological replicates (including IB). **P < 0.01 indicates statistical significance. Data in C, D, E, H, I, J were analyzed using a two-way ANOVA test. Data in L were analyzed using a one-way ANOVA test.

Then, we investigated whether the accumulation of endogenous glutamate following the inhibition of system X_C_^-^ is linked to the suppression of YAP in LUAD cells. Cystine can be taken up by the system X_C_^-^ and converted into cysteine for the synthesis of GSH, one of the major intracellular antioxidants 35. Blocking system X_C_^-^ eventually leads to a shortage of GSH, which is also a contributor to ferroptosis. Adding β-ME, a compound that circumvents the inhibition of system X_C_^-^ by promoting the cystine to cysteine transition and the inflow of cysteine uptake through an alternative pathway 36, failed to rescue erastin suppression of YAP in H1975 cells (Figure S2D). Similarly, the addition of NAC, the precursor of cysteine and GSH, did not attenuate YAP suppression (Figure S2D). Thus, impaired cystine uptake and GSH synthesis following the inhibition of system X_C_^-^ was further excluded as the mechanism of YAP suppression. To address whether the accumulation of endogenous glutamate contributes to YAP suppression in LUAD cells, we tried to eliminate endogenous glutamate in LUAD cells. A mechanism including uptake, deamination and transamination maintains glutamate homeostasis (illustrated in Figure 2A). Forced overexpression of the transaminases GOT2 and GPT2 failed to decrease glutamate in H1975 cells (Figure S2E). In contrast, overexpressing GLUD1, which is a dehydrogenase mediating glutamate deamination significantly reduced glutamate, but at an unsatisfactory level (Figure S2F). GLAST and GLT1, which are transporters that absorb glutamate, can be induced by a feedback mechanism involving an increase in GLUD1 37. Only when GLAST and GLT1 were simultaneously knocked down was the endogenous glutamate content reduced to a very low level (Figure S2F). Glutamate is physiologically important to tumor cells 38; thus, we constructed LUAD cell-based Dox-inducible GGG cells in which GLUD1 overexpression (G) and GLAST (G) and GLT1 knockdown (G) was simultaneously achieved (illustrated in Figure 2B and Figure S2G). To exclude whether Dox could induce ferroptotic changes in LUAD cells beyond the intracellular glutamate, we treated LUAD cells with or without Dox, and noticed that Dox with a concentration of at 1 µg/ml, which was exactly the same as that for GGG induction did not affect cell viability, cell death and generation of lipid ROS and MDA in LUAD cells (Figure S2H-K), demonstrating that Dox might not interfere the roles of glutamate on ferroptosis. To reconstitute intracellular glutamate in the Dox-induced GGG cells, we added increasing concentrations of glutamate. Unfortunately, even at a concentration of 16 mM, the intracellular glutamate was still not elevated (Figure S2L), which may be due to impaired glutamate uptake resulting from the deficiency of GLAST and GLT1. However, inhibiting GLUD1 with the inhibitor EGCG successfully reversed intracellular glutamate to the basal level (Figure 2C). Using the Dox/GGG/EGCG cell models, we found that Dox induction successfully abolished endogenous glutamate accumulation, which was triggered 1 h following erastin treatment and could be sustained for 24 h in H1975 cells (Figure S2M). We also observed that the accumulation of endogenous glutamate following erastin and Sorafenib treatment was relied on SLC7A11 and SLC3A2, two subunits of system X_C_^-^ (Figure S2N-O). Cell proliferation was not involved during the timeframe of our experiment (Figure S2P). However, ferroptosis sensitivity and variation of MDA was suppressed following endogenous glutamate depletion and was restored by EGCG (Figure 2D-E). These results resembled those when erastin suppression of YAP was blocked (Figure 1J-K), indicating that YAP suppression is a result of endogenous glutamate accumulation.

Subsequently, we investigated how endogenous glutamate accumulation suppresses YAP. Glutamate accumulation did not affect the mRNA level but stimulated Hippo pathway-like YAP suppression (Figure S1L-O), and O-GlcNAcylation at the Thr241 site stabilized YAP by antagonizing its Hippo-dependent phosphorylation 20; thus, we speculated that endogenous glutamate accumulation suppresses YAP by inhibiting its O-GlcNAcylation. Indeed, O-YAP^T241^ and total YAP were both substantially reduced following treatment with erastin, and these effects were diminished upon depletion of glutamate. In contrast, p-YAP^S127^, the major phosphorylation site targeted by the Hippo pathway, was increased by erastin in H1975 cells in a glutamate dependent manner (Figure 2F). Replacement of the Thr241 by alanine (A) blocked erastin-induced and glutamate-dependent suppression of YAP O-GlcNAcylation (Figure 2G), indicating that Thr241 is the major affected O-GlcNAcylation site following system X_C_^-^ inhibition. Notably, endogenous glutamate deletion desensitized LUAD cells to the erastin suppression of O-YAP^T241^ and total YAP (Figure 2H-I). Similar effects were observed for the elevation of p-YAP^S127^ (Figure 2J). These results suggested that a reduction in O-GlcNAcylation is a prerequisite for endogenous glutamate-induced YAP suppression. However, glutamate-dependent suppression of YAP was not rescued by Fer-1 (Figure 2F), indicating that this suppression is not an effect of lipid ROS generation. After reconstituting WT- or T241A-YAP in *YAP^-/-^* H1975 cells, we noticed that introducing the T241A mutation desensitized the H1975 cells to erastin treatment (Figure 2K-L), indicating that O-GlcNAcylation of YAP at Thr241 is the target to increase ferroptosis sensitivity.

Finally, because glutamate is the intermediate metabolite between glutamine and alpha-ketoglutaric acid (α-KG) (illustrated in Figure 2A), we investigated whether endogenous glutamate suppression of YAP is mediated by glutamine and α-KG. Intracellular glutamine was comparable before and after erastin treatment in LUAD cells (Figure S2Q). Blocking α-KG influx by treatment with AOA, a pan-inhibitor of transaminase, as well as simultaneous supplementation with DMK, a precursor of α-KG that is more cell permeable, failed to alter the erastin-induced suppression of YAP (illustrated in Figure 2A and Figure S2R). Thus, the suppression of YAP following system X_C_^-^ inhibition is a specific effect of glutamate.

### GFPT1 mediates glutamate-induced suppression of YAP O-GlcNAcylation to modulate ferroptosis sensitivity

**Figure 3:**
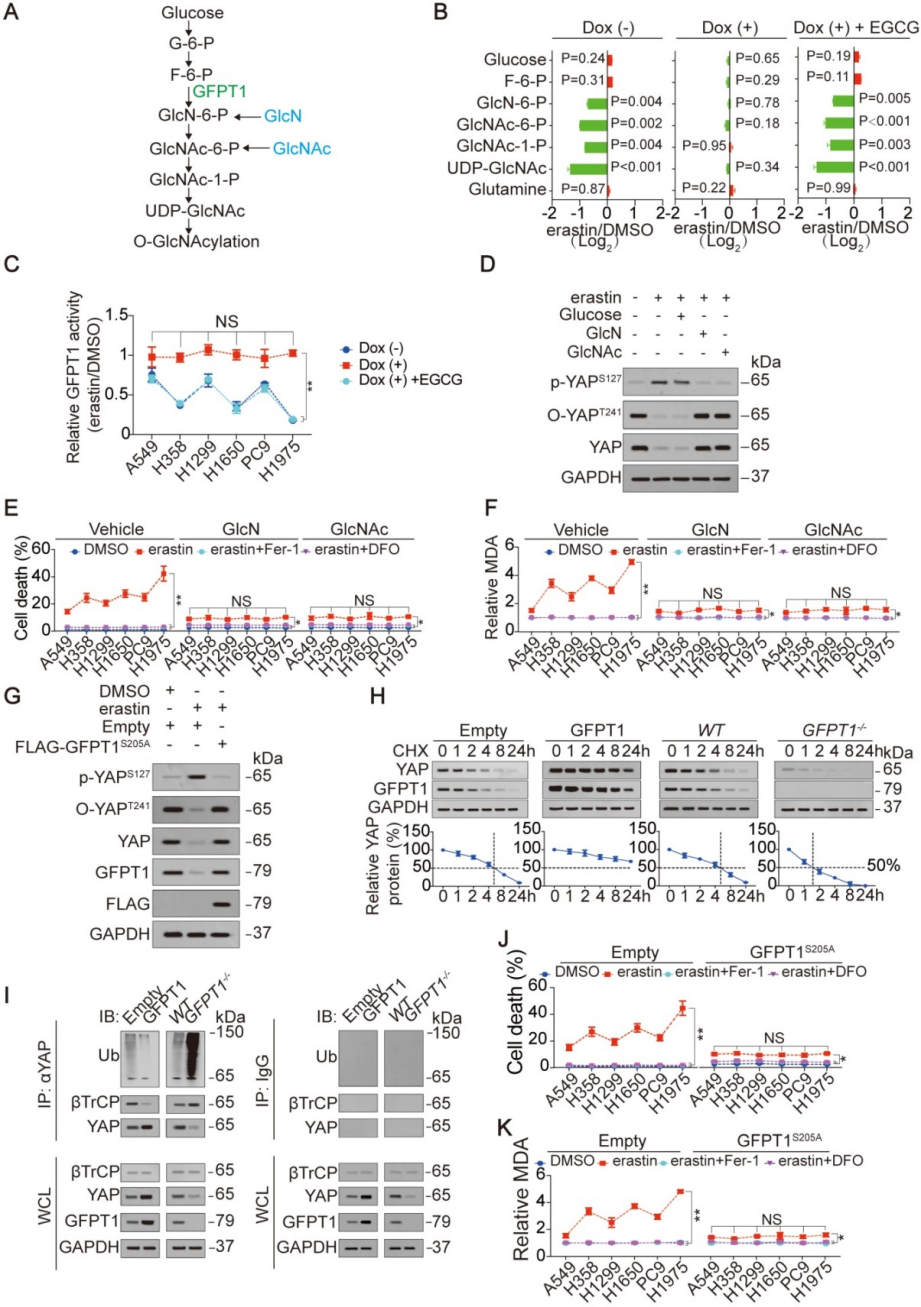
** GFPT1 is critical for endogenous glutamate to determine ferroptosis sensitivity. (A)** Schematic overview of the HBP signaling from glucose to O-GlcNAcylation. **(B)** Endogenous glutamate affects HBP metabolites. Indicated metabolites were measured in H1975-based GGG cells under the same treatment as that in Figure 2D. **(C)** Relative GFPT1 activity was measured in GGG cells under the same treatment as that in Figure 2D but treated with or without erastin (10 µM) for 8 h. The GFPT1 activity was normalized between those treated with erastin and DMSO. **(D)** Metabolites downstream of GFPT1 restore YAP alteration by erastin. p-YAP^S127^, O-YAP^T241^ and YAP were measured by IB in H1975 cells treated with or without erastin (10 µM), glucose (25 mM), GlcN (5 mM) or GlcNAc (5 mM) for 8 h. **(E-F)** GlcN and GlcNAc diminish ferroptosis sensitivity. LUAD cells were treated with erastin (10 µM) with or without Fer-1 (1 µM) or DFO (80 µM), in the presence or absence of GlcN (5 mM) or GlcNAc (5 mM). Cell death was measured after treatment for 24 h (E). MDA was measured after treatment for 16 h (F). **(G)** GFPT1^S205A^ restores YAP alteration by erastin. FLAG-GFPT1^S205A^ was ectopically expressed in H1975 cells prior to the treatment with or without erastin (10 µM) for 8 h. Indicated proteins were analyzed by IB. **(H)** GFPT1 boosts protein stability of YAP. CHX (10 µg/ml) chase experiments were performed in H1975 cells with or without GFPT1 overexpression or knockout. The relative protein levels of YAP were shown as the ratios between YAP and GAPDH, and the “0 h” points were arbitrarily set to 100%. **(I)** GFPT1 reduces ubiquitination of YAP. The ubiquitination of YAP was measured in control cells and H1975 cells with or without GFPT1 overexpression or knockout using anti-Ub antibodies following immunoprecipitation using anti-YAP antibodies. IgG antibodies were used as negative controls. **(J-K)** Restore of GFPT1 diminishes ferroptosis sensitivity. FLAG-GFPT1^S205A^ was ectopically expressed in LUAD cells before treatment with erastin (10 µM) with or without Fer-1 (1 µM) or DFO (80 µM). Cell death was measured after treatment for 24 h by SYTOX green staining followed by flow cytometry (J). MDA was measured after treatment for 16 h (K). The data are shown as the mean ± SD from three biological replicates (including IB). **P < 0.01 indicates statistical significance. Data in B were analyzed using a one-way ANOVA test. Data in C, E, F, J, K were analyzed using a two-way ANOVA test.

Here, we explored the mechanism underlying how the accumulation of endogenous glutamate suppresses the O-GlcNAcylation of YAP. The influx of O-GlcNAcylation is determined by the HBP (illustrated in Figure 3A and Ref. 23). After testing metabolites that belong to the HBP, we found an endogenous glutamate-dependent decrease in metabolites generated during the change from GlcN-6-P to UDP-GlcNAc following treatment with erastin in H1975 cells (Figure 3B). These metabolites are downstream of GFPT1 (illustrated in Figure 3A). In contrast, only a slight accumulation of metabolites upstream of GFPT1, namely, glucose and F-6-P, was observed (Figure 3B), implying that metabolism downstream of GFPT1 is obstructed. GFPT1 catalyzes the amidation of F-6-P to GlcN-6-P in the presence of glutamine. However, because glutamine is not utilized, the glutamine level was not significantly changed (Figure 3B and Figure S2Q). Similar to that of YAP O-GlcNAcylation and total YAP (Figure 2H-I), the degree of GFPT1 activity suppressed by erastin was also glutamate-dependent in LUAD cells (Figure 3C), therefore, we hypothesized that the glutamate-dependent suppression of YAP and the reduction in its O-GlcNAcylation are mediated by the suppression of GFPT1 following inhibition of system X_C_^-^. To test this supposition, we circumvented GFPT1 inhibition by replenishing downstream metabolites. Although supplementation with GlcN and GlcNAc, which can bypass GlcN-6-P and GlcNAc-6-P synthesis 39, failed to restore GFPT1 activity in H1975 cells (Figure S3A), erastin induced the induction of p-YAP^S127^, and the reductions in O-YAP^T241^ and total YAP were thus diminished (Figure 3D). However, the glucose level did not influence YAP (Figure 3D). At the genetic level, the reconstitution of GFPT1 by an erastin-resistant GFPT1^S205A^ (will be discussed in the following section) successfully restored GFPT1 activity and expression (Figure 3G and Figure S3B). This change also prevented the phosphorylation and suppression of YAP following system X_C_^-^ inhibition in H1975 cells (Figure 3G). Because changes of YAP modifications influence its protein stability 18, 20, 28, we assessed the half-life of YAP before and after alteration of GFPT1 expression. We found that GFPT1 prolonged half-life of YAP in H1975 cells, suggesting that GFPT1 boosts protein stability of YAP (Figure 3H), and such effects might be a result from preventing βTrCP-YAP interaction and subsequent reduction of YAP ubiquitination (Figure 3I). In summary, endogenous glutamate-induced suppression of YAP and its O-GlcNAcylation are mediated by GFPT1 following the inhibition of system X_C_^-^.

Next, we assessed whether suppression of GFPT1 determines ferroptosis sensitivity. As shown in Figure 3E-F and 3J-K, ferroptosis sensitivity and ferroptosis-associated MDA level variation following inhibition of system X_C_^-^ were abolished by either supplementation with GlcN and GlcNAc or the reconstitution of GFPT1 via GFPT1^S205A^ in LUAD cells, demonstrating that the suppression of GFPT1 is critical for determining ferroptosis sensitivity.

To confirm that the suppression of GFPT1 in LUAD cells is a genuine result of system X_C_^-^ inhibition following erastin treatment, LUAD cells were also treated with Sorafenib and RSL3. Similar to the effect of erastin, Sorafenib but not RSL3 reduced GFPT1 activity (Figure S3C), and GFPT1^S205A^ reversed this effect in H1975 cells (Figure S3D), further demonstrating that the suppression of GFPT1 is indeed occurred following the inhibition of system X_C_^-^.

### Ineffective XBP1 splicing facilitates glutamate-dependent GFPT1 suppression in LUAD cells

**Figure 4:**
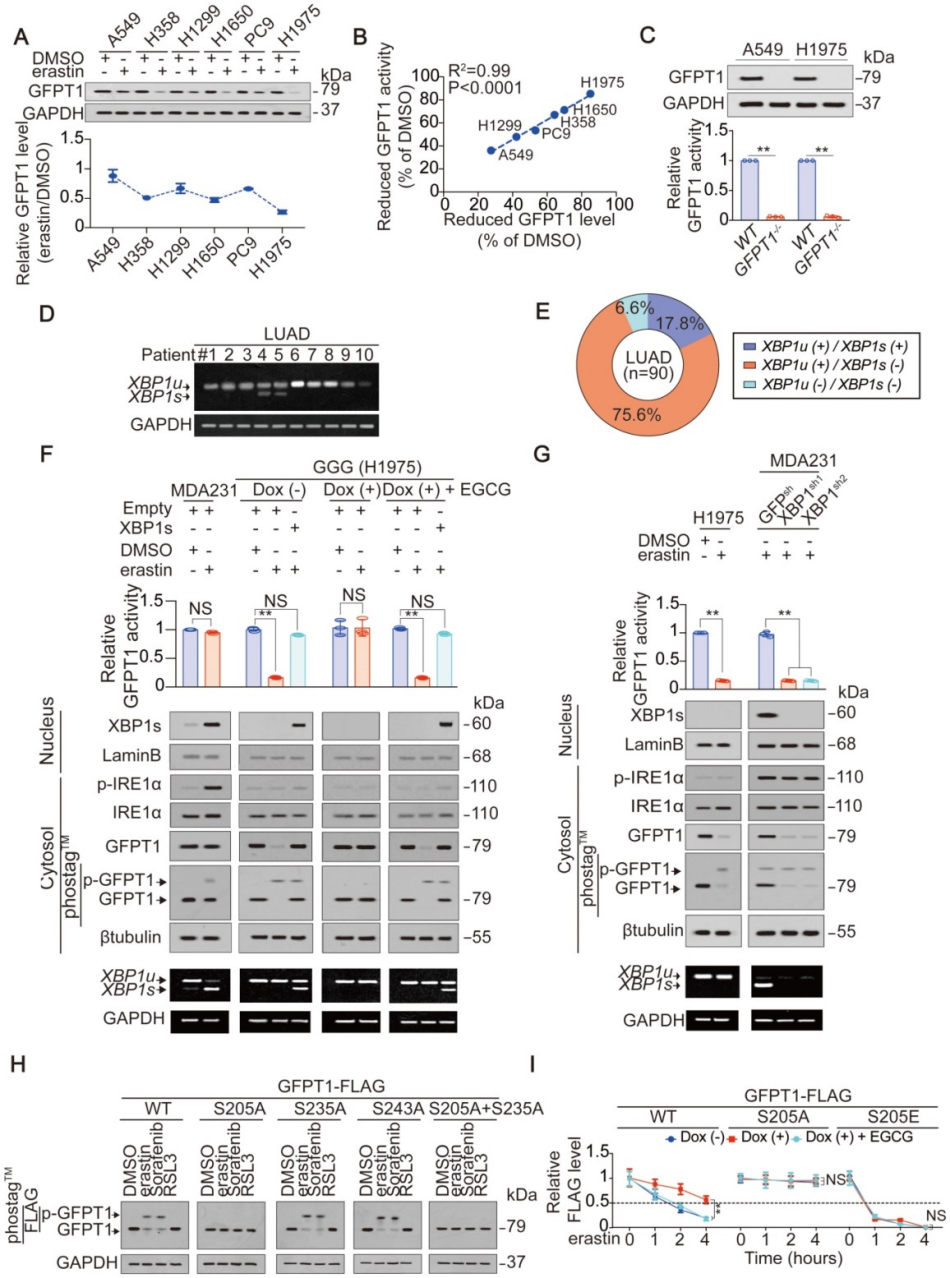
** Ineffective XBP1 splicing in LUAD cells facilitates glutamate suppression of GFPT1. (A)** IB of GFPT1 in LUAD cells treated with DMSO or erastin (10 µM) for 8 h. The level of GFPT1 was normalized to that of GAPDH, and the normalized level of GFPT1 in DMSO-treated A549 cells was arbitrarily set to 1. **(B)** Reduced activity and expression of GFPT1 by erastin are closely correlated, and were calculated as the percentage to the ones treated with DMSO for 8 h. **(C)** GFPT1 expression and activity were analyzed using IB and GDH assay method in *WT* and *GFPT1^-/-^* A549 and H1975 cells. **(D)** Spliced (s) or unspliced (u) form of *XBP1* mRNA in LUAD specimens, as measured by semi-RT-qPCR via agarose gel electrophoresis. **(E)** Percentage of LUAD specimens with indicated *XBP1* splicing conditions. **(F)** Ineffective XBP1 splicing facilitates GFPT1 decrease. H1975-based GGG cells with or without ectopically expressed XBP1s were under the same treatment as that in Figure 2D but treating with or without erastin (10 µM) for 8 h. MDA-MB-231 cells were treated as the paralleled control. Relative GFPT1 activity was analyzed utilizing GDH assay method. Total and phosphorylated GFPT1 in nuclear and cytosolic fractions were analyzed by IB using normal gels or gels containing phostag™. **(G)** XBP1 was knocked down in MDA-MB-231 cells before treating with erastin (10 µM) for 8 h. H1975 cells were treated as the paralleled control. GFPT1 activity, total and phosphorylated GFPT1 were analyzed the same way as that in Figure 4F. **(H)** GFPT1 is phosphorylated at S205 by erastin and Sorafenib. Phosphorylation of indicated GFPT1-FLAG was analyzed by IB using gels containing phostag™ in H1975 cells after treating with or without erastin (10 µM), Sorafenib (5 µM) and RSL3 (5 µM) for 8 h. **(I)** Phosphorylation at S205 is critical for GFPT1 degradation. The degradation of indicated GFPT1-FLAG was measured by IB in H1975-based GGG cells after pretreating with or without Dox and EGCG for 24 h, before further treating with erastin (10 µM) for indicated hours. GFPT1 level was normalized to that of GAPDH. The data are shown as the mean ± SD from three biological replicates (including IB and semi-RT-qPCR). **P < 0.01 indicates statistical significance. Data in C, F, G were analyzed using a one-way ANOVA test. Data in I were analyzed using a two-way ANOVA test. Data in B were analyzed using the Spearman rank-correlation analysis.

Although glutamate-dependent suppression of YAP and ferroptosis sensitivity is mediated by suppression of GFPT1 in LUAD cells (Figure 3), the mechanism underlying how GFPT1 suppression is boosted following inhibition to system X_C_^-^ in LUAD cells still remains unclear. We found that the downregulation of GFPT1 expression correlated with the suppression of GFPT1 activity in LUAD cells following treatment with erastin (Figure 3C and 4A-B). Knocking out GFPT1 simultaneously lead to diminished GFPT1 activity in A549 and H1975 cells (Figure 4C), further supporting the idea that the suppression of GFPT1 activity is a result of the previous suppression of its expression. Therefore, we hereafter focused on the mechanism by which GFPT1 expression is decreased following the inhibition of system X_C_^-^. The expression of GFPT1 can be transcriptionally sustained by the XBP1s under endoplasmic reticulum (ER) stress, such as that generated by glucose deprivation or hypoxia 40. Without effective XBP1 splicing, GFPT1 level usually decreases rapidly 21, 41. Interestingly, the inhibition of system X_C_^-^, such as that induced by erastin, can also induce ER stress 31. Therefore, we hypothesized that the suppression of GFPT1 expression is boosted by ineffective XBP1 splicing following treatment with erastin. To test this supposition in LUAD samples, we first examined the levels of *XBP1u* and *XBP1s* mRNAs, which encode the unspliced and spliced forms of the XBP1 protein, respectively 31. As expected, compared to the *XBP1u* mRNA, the *XBP1s* mRNA was absent in LUAD at a higher frequency (Figure 4D-E). Because IRE1α processes *XBP1* transcripts that result in *XBP1s* mRNAs upon ER stress, and since the autophosphorylation of IRE1α (p-IRE1α) is an indicator of its activation 42, we measured the p-IRE1α and total-IRE1α levels to evaluate whether inactivation of IRE1α is the reason for ineffective XBP1 splicing following treatment with erastin in LUAD cells. The MDA-MB-231 breast cancer cell line was used as a positive control cell line because it displays highly efficient IRE1α activation and *XBP1* mRNA splicing under ER stress 40, and glucose deprivation and hypoxia were set as the positive control conditions for ER stress. In contrast to its status in MDA-MB-231 cells, IRE1α was inactivated, and the splicing of *XBP1* mRNA was ineffective upon glucose deprivation, hypoxia or erastin treatment (Figure 4F-G and Figure S4A-D). In contrast to XBP1u, XBP1s can be translocated into the nucleus and act as a transcription factor 21. Compared to its localization in MDA-MB-231 cells, the XBP1s protein was also not obviously observed in the nuclear fraction of the H1975 cells under glucose deprivation conditions and the treatment with erastin, reasoning that *XBP1s* mRNA was inefficiently generated (Figure 4F-G and Figure S4E). Because XBP1s sustains GFPT1 expression by stimulating *GFPT1* transcription under ER stress 21, we assessed *GFPT1* promoter activity and mRNA level by testing the luciferase activity of a reporter containing the *GFPT1* promoter and performing qPCR, and found that the treatment of MDA-MB-231 cells with erastin resulted in significant promoter activity elevation and mRNA expression of *GFPT1*; however, this effect was absent in LUAD cells (Figure S4F-G), further demonstrating that *XBP1* mRNA splicing is relatively ineffective in LUAD cells. To further investigate whether ineffective XBP1 splicing facilitates the suppression of GFPT1 expression following treatment with erastin, we replenished XBP1s in H1975 cells, and found that the impaired GFPT1 expression and activity was diminished (Figure 4F), which might be the result of XBP1s-mediated stimulation of *GFPT1* promoter activity (Figure S4H). Intriguingly, depletion of glutamate had no influence on IRE1α activation or XBP1s generation in H1975 cells (Figure 4F), hinting that ineffective XBP1 splicing might be a specific and glutamate-independent phenomenon in LUAD cells. Oppositely, similar to the results in H1975 cells, GFPT1 expression and activity could not be sustained upon XBP1 knockdown in the MDA-MB-231 cells after treatment with erastin (Figure 4G). Collectively, these results imply that ineffective XBP1 splicing in LUAD cells facilitates the suppression of GFPT1 following the inhibition of system X_C_^-^.

As described above, inefficient splicing of *XBP1* mRNA boosts GFPT1 reduction in LUAD cells (Figure 4D-G); however, how the reduction in GFPT1 is triggered following the inhibition of system X_C_^-^ remains elusive. Posttranslational modifications (PTMs) are critical for protein turnover, and phosphorylation is one of the most prevalent ones 43, 44. Reasoning that GFPT1 can be phosphorylated 24, we explored whether phosphorylation of GFPT1 is also influenced by glutamate following the inhibition of system X_C_^-^. Indeed, the phosphorylation of GFPT1 was induced by erastin in a glutamate-dependent manner (Figure 4F-G), suggesting that the phosphorylation of GFPT1 might be a prerequisite for its suppression. GFPT1 can be phosphorylated at Ser205, Ser235 and Ser243 24, 45. We wondered whether phosphorylation at these sites is affected by system X_C_^-^ inhibition. Treating H1975 cells with erastin or Sorafenib, but not RSL3, resulted in significant phosphorylation of GFPT1^wild-type (WT)^; however, these treatments were ineffective when the Ser205 was replaced by an alanine (S205A) (Figure 4H). Interestingly, the accumulation of endogenous glutamate triggered by erastin treatment accelerated the degradation of GFPT1^WT^ (Figure 4I and Figure S4I). Compared to the GFPT1^WT^, GFPT1^S205A^ is much more stable and shows resistance to glutamate. Serine is often mutated to glutamic acid (E) to mimic the phosphorylation of the serine residue. As expected, GFPT1^S205E^ was still glutamate resistant; however, the degradation of this mutant was the fastest among the variants (Figure 4I and Figure S4I). Overall, GFPT1 suppression is triggered by glutamate-dependent phosphorylation at Ser205, which facilitates its degradation following system X_C_^-^ inhibition in LUAD cells.

### ADCY10 is the key factor in the glutamate-dependent phosphorylation of GFPT1 and reflects the ferroptosis sensitivity of LUAD cells

**Figure 5:**
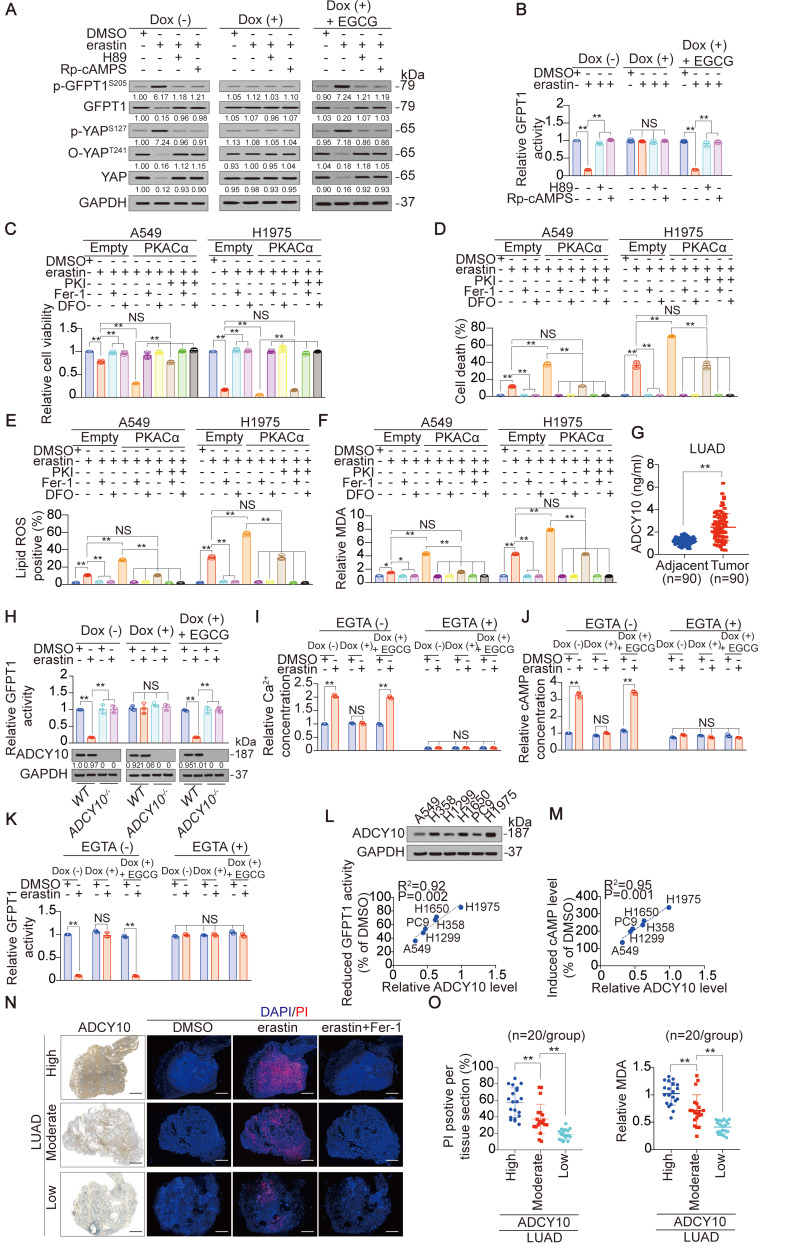
** ADCY10 PKA-dependently determines ferroptosis sensitivity in LUAD cells. (A)** Endogenous glutamate phosphorylates GFPT1 via PKA. H1975-based GGG cells were under the same treatment as that in Figure 2C, before further treating with or without erastin (10 µM), in the presence or absence of H89 (10 µM) and Rp-cAMPs (200 µM) for 8 h. Indicated proteins were analyzed by IB. **(B)** GFPT1 activity was analyzed by GDH method in H1975-based GGG cells under the same treatment as that in panel A. **(C-F)** PKA boosts erastin-induced ferroptosis. A549 and H1975 cells ectopically expressing PKACα were treated with erastin (10 µM), in the presence or absence of PKI (10 µM), Fer-1 (1 µM) or DFO (80 µM). Cell viability (C) and cell death (D) were analyzed 24 h after treatment, and lipid ROS (E) and MDA generations (F) were analyzed 16 h after treatment. **(G)** ADCY10 level were analyzed in 90 paired LUAD and their adjacent specimens. **(H)** *WT* or *ADCY10^-/-^* H1975-based GGG cells were under the same treatment as that in Figure 2D but treating with or without erastin (10 µM) for 8 h. Relative GFPT1 activity was analyzed utilizing GDH method, and ADCY10 was analyzed by IB. The level of proteins was normalized to that of GAPDH, and the normalized level of proteins in DMSO-treated cells was arbitrarily set to 1. **(I-K)** H1975-based GGG cells were pretreated with or without EGTA (0.1 mM), Dox (1 µg/ml) and EGCG (5 µM) for 24 h before further treating with erastin (10 µM) for 8 h. Relative Ca^2+^ (I) and cAMP concentration (J), and GFPT1 activity (K) were then measured. **(L-M)** ADCY10 expression correlates with reduced GFPT1 activity and induced cAMP following erastin (10 µM) treatment for 8 h. Reduced GFPT1 activity (L) and induced cAMP (M) were shown as the percentage to the DMSO-treated control, and ADCY10 was measured by IB in indicated LUAD cells. **(N)** Representative micrograph of LUAD tissue sections with different ADCY10 level treating with erastin (10 µM) with or without Fer-1 (1 µM) for 24 h. ADCY10 level was measured by IHC. Ferroptotic cells and nuclei were visualized using PI (red) and DAPI (blue). PI positive area from each sample was also calculated as the percentage to the whole section and graphed on the right. Scale bar, 1 mm. **(O)** MDA concentration was measured in the same sample as that in the panel N (n = 20/group). The data are shown as the mean ± SD from three biological replicates (including IB). **P < 0.01 indicates statistical significance. Data in B, C, D, E, F, H, I, J, K, N, O were analyzed using a one-way ANOVA test. Data in G were analyzed using a Student's t test. Data in L, M were analyzed using the Spearman rank-correlation analysis.

Although GFPT1 can be phosphorylated at Ser205 (Figure 4F-H), how it happens via glutamate following inhibition of system X_C_^-^ is unclear. Reasoning that p-GFPT1^S205^ is modulated by PKA 34, we investigated whether PKA is also involved in this phosphorylation following system X_C_^-^ inhibition. First, antibodies specifically recognizing p-GFPT1^S205^ were developed (Figure S5A-B). Consistent with the results from electrophoresis using Phostag™-containing gel (Figure 4F), a glutamate-dependent increase in p-GFPT1^S205^ following erastin treatment was observed with an anti-p-GFPT1^S205^ antibody (Figure 5A). Treating H1975 cells with H89 and Rp-cAMPS, two structurally distinct PKA inhibitors targeting the ATP-binding site and cAMP-binding site, respectively 46, 47, successfully blocked the glutamate-dependent induction of p-GFPT1^S205^ (Figure 5A). YAP and GFPT1 expression and activity were also suppressed in a PKA-dependent manner (Figure 5A-B). If PKA truly phosphorylates GFPT1 and facilitates its subsequent suppression, stimulating PKA should sensitize cells to erastin-triggered ferroptosis. Therefore, at the genetic level, PKACα, the catalytic subunit of PKA, was overexpressed in A549 and H1975 cells prior to erastin treatment (Figure S5C). Indeed, ferroptosis and ferroptosis-associated lipid ROS and MDA generation were enhanced in the PKAα-overexpressing A549 and H1975 cells (Figure 5C-F). However, these effects were prevented when PKI, a potent synthetic peptide inhibitor of PKA, was simultaneously added (Figure 5C-F). These results suggested that GFPT1 is phosphorylated by PKA in a glutamate-dependent manner following inhibition of system X_C_^-^.

PKA receives upstream signals from cAMP, the formation of which is catalyzed by ADCYs 48, 49. We therefore investigated the possibility that ADCY is responsible for PKA-dependent suppression of GFPT1. According to the UALCAN database, among the 10 ADCYs evaluated, only ADCY10 is upregulated in LUAD tissue, compared to normal lung tissue (Figure S5D). This finding was extended to include the paired tumor and adjacent tissue samples (Figure 5G). Additionally, only knocking out ADCY10 resulted in H1975 cell resistance to erastin suppression of GFPT1 activity (Figure S5E-F), and this effect was glutamate-dependent (Figure 5H). Furthermore, ADCY10 was identified as the driver of cAMP induction following the inhibition of system X_C_^-^ because the effects of erastin- and Sorafenib-induced elevation of cAMP were abolished in ADCY10-knockout H1975 cells (Figure S5G). In addition, we noticed that the inhibition of GPX4 by either siRNA-mediated *GPX4* gene silencing or RSL3 did not alter ADCY10 expression or the cAMP production dependent on it (Figure S5G-H), further explaining why the subsequent suppression of YAP was not observed following the inhibition of GPX4 (Figure S1K and 1N-O). In summary, ADCY10 is the target of glutamate, acting upstream to produce cAMP for the PKA-dependent suppression of GFPT1 following the inhibition of system X_C_^-^.

Then, we explored how glutamate stimulates ADCY10. Because the accumulation of glutamate has the capacity to elevate intracellular Ca^2+^, which is a genuine stimulator of ADCY10 50, we speculated that glutamate stimulates ADCY10 by increasing Ca^2+^. To test this hypothesis, we measured intracellular Ca^2+^, cAMP and GFPT1 activity following erastin treatment in the presence or absence of EGTA, a chelator of calcium. The glutamate-dependent increase in Ca^2+^ and cAMP levels and reduction in GFPT1 activity were diminished in the presence of EGTA (Figure 5I-K), indicating that increasing Ca^2+^ is key to the glutamate stimulation of ADCY10 and the functions that depend on ADCY10.

Due to the critical roles of ADCY10 in suppressing GFPT1 (Figure 5H), we wondered whether ADCY10 expression is affected by the inhibition of system X_C_^-^. Unfortunately, ADCY10 expression was not affected by either erastin or Sorafenib in LUAD cells (Figure S5H-I), suggesting that the inhibition of system X_C_^-^ is not sufficient to alter ADCY10 expression. The forced overexpression of ADCY10 was similarly unable to trigger cell death or alter lipid ROS generation in H1975 cells (Figure S5J), implying that ADCY10 activation per se might not be sufficient to induce ferroptosis. However, whether ADCY10 augments ferroptosis following the inhibition of system X_C_^-^ remains unclear. We found that the degree to which erastin reduced GFPT1 activity and induced cAMP concentration was significantly correlated with basal ADCY10 levels (Figure 5L-M). By ectopically expressing increasing concentrations of ADCY10 in H1975 cells, we found that the higher the ADCY10 expression was, the greater the ability of erastin to suppress O-YAP^T241^, total YAP and GFPT1 while induce increase in cAMP concentration (Figure S5K-M). These results demonstrated that ADCY10 sensitizes cells to ferroptosis and that the ADCY10 concentration-dependent production of cAMP determines the degree to which downstream suppression of GFPT1 following inhibition of system X_C_^-^. Notably, intracellular *ADCY10* mRNA levels also reflected erastin suppression of GFPT1 in LUAD cells (Figure S5N), suggesting that simply performing qPCR to evaluate *ADCY10* mRNA can predict sensitivity to ferroptosis in LUAD patients.

Subsequently, slice culture of primary LUAD tissue was performed to assess the role of ADCY10 in determining ferroptosis sensitivity *ex vivo*. By comparing the responses to ferroptosis in the LUADs with 3 distinct ADCY10 levels (n = 20/group), we found that erastin tended to induce ferroptosis and MDA level in the LUAD samples with high ADCY10 expression (Figure 5N-O). ADCY10 is a possible independent indicator for predicting cell sensitivity to ferroptosis because erastin-accumulated glutamate was similar among the LUADs regardless of ADCY10 expression (Figure S5O).

### Ferroptosis is beneficial for advanced-stage and therapy-resistant LUAD because of elevated ADCY10 levels

**Figure 6:**
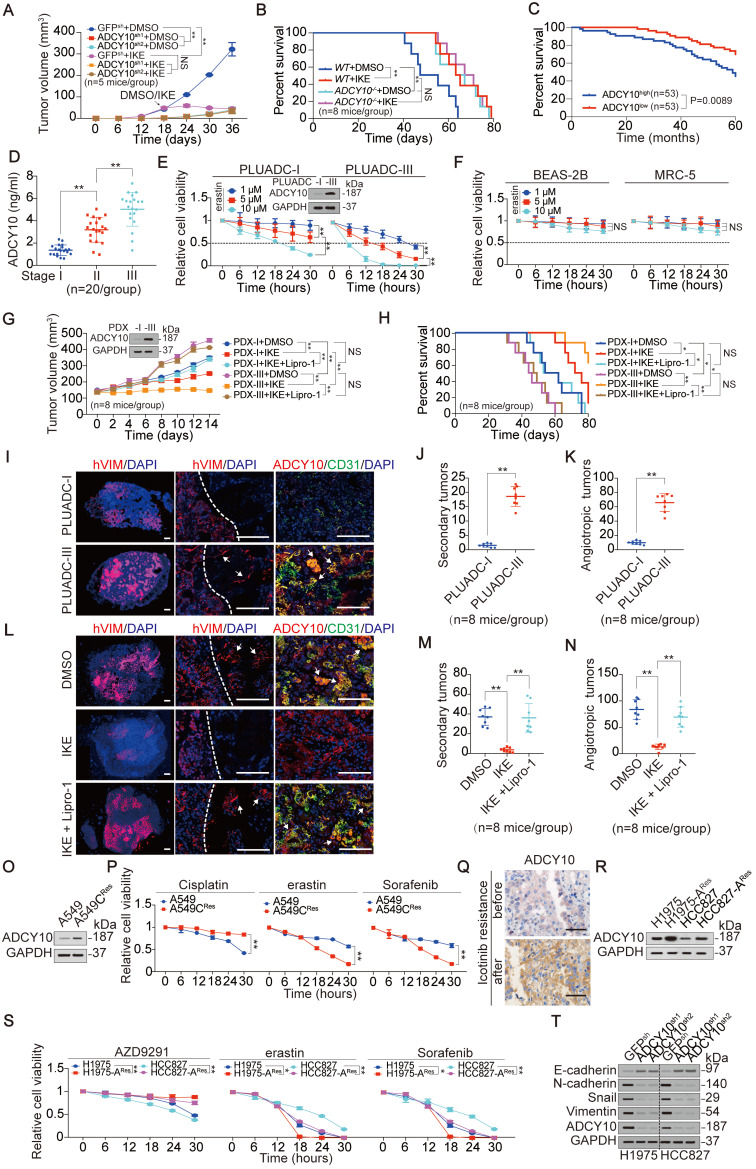
** LUADs with higher ADCY10 expression are more susceptible to ferroptosis. (A)** Volume of xenograft formed by H1975 cells with or without knocking down ADCY10 was monitored after inoculation for indicated days. DMSO or IKE (50 mg/kg) was administrated once every day for 14 days began at day 18 after inoculation. (n = 5/group). **(B)** Survival of mice bearing xenograft formed by H1975 cells with or without knocking out ADCY10 was monitored after inoculation for indicated days, DMSO or IKE (50 mg/kg) was administrated once every day for 14 days began at day 18 after inoculation. (n = 8/group). **(C)** Survival in LUAD patients with ADCY10 high (n = 53) or low (n = 53) expression after curative surgery. **(D)** ADCY10 level was measured in stage I, II and III LUADs (n = 20/group). **(E)** Cell viability was assayed by measuring cellular ATP in primary LUAD cells after treating with increasing concentrations of erastin for indicated hours. **(F)** Cell viability was measured in BEAS-2B or MRC-5 cells under the same treatment as that in panel E. **(G)** Volume of PDX for indicated times after treating with IKE (50 mg/kg) with or without Lipro-1 (10 mg/kg) once every day for 14 days (n = 8/group). **(H)** Survival of PDX-bearing mice after treating with IKE (50 mg/kg) with or without Lipro-1 (10 mg/kg) once every day for 14 days (n = 8/group). **(I)** Representative micrograph of lung sections from mice intrapleurally injected with PLUADC-I/III cells for 36 days. Proteins were stained as indicated. Dashed lines separate the primary tumor mass and the normal lung. Nuclei were visualized by DAPI (blue). Arrows in middle graph point to the secondary tumors. Arrows in right graph point to the ADCY10 and CD31 co-expressing angiotropic tumors (n = 8/group). Scale bar, 500 µm. **(J-K)** Quantification of the number of hVIM^+^ secondary tumors (J) and ADCY10^+^/CD31^+^ angiotropic tumors (K) detected in the whole lungs, which are the same as those in panel I (n = 8/group). **(L)** Representative micrograph of lung sections from mice intrapleurally injected with PLUADC-III cells for 24 days before administration with IKE (50 mg/kg) with or without Lipro-1 (10 mg/kg) for another 14 days. (n = 8/group). Scale bar, 500 µm. **(M-N)** Quantification of the number of PLUADC-III formed hVIM^+^ secondary tumors (M), and ADCY10^+^/CD31^+^ angiotropic tumors (N) under the same treatment as those in panel L (n = 8/group). **(O-P)** ADCY10 expression (O) and relative cell viability (P) were measured in A549 Cisplatin resistant (C^Res^) and parental cells treating with or without Cisplatin (10 µM), erastin (10 µM) or Sorfenib (5 µM) for indicated hours. **(Q)** ADCY10 expression was measured in LUAD tissues before and after Icotinib resistance from the same patient by IHC. **(R-S)** ADCY10 expression (R) and relative cell viability (S) was measured in parental and AZD9291 resistant (A^Res^) H1975 and HCC827 cells treating with or without AZD9291 (10 µM), erastin (10 µM) or Sorfenib (5 µM) for indicated hours. **(T)** ADCY10 promotes EMT. EMT indicators were measured by IB in H1975 and HCC827 cells with or without ADCY10 knocked down. The data are shown as the mean ± SD from three biological replicates (including IB). *P < 0.05, **P < 0.01 indicates statistical significance. Data in D, J, K, M, N were analyzed using a one-way ANOVA test. Data in A, E, F, G, P, S were analyzed using a two-way ANOVA test. Data in B, C, H were analyzed using the log rank analysis.

ADCY10 is upregulated in LUAD tissues compared to adjacent lung tissues (Figure 5G); however, its expression is also correlated with ferroptosis sensitivity of LUAD cells (Figure 5). Because LUAD cells are more sensitive to ferroptosis than lung fibroblast cells (Figure 1A-E and Figure S1A-D), we wondered whether ADCY10 is also protumorigenic. To test this possibility, we impaired ADCY10 expression in LUAD cells and performed *in vitro* and *in vivo* functional experiments. Knocking down ADCY10 by two independent shRNAs (Figure S6A) significantly impaired anchorage-independent growth of H1975 cells in soft agar (Figure S6B), spheroid formation in 3D culture (Figure S6C) and tumor growth in CDX mouse models (Figure 6A and Figure S6D). Limiting dilution cell transplantation assays demonstrated that ADCY10 deficiency in H1975 cells also decreased the frequency of tumor incidence in mice (Figure S6F). While knocking out ADCY10 resulted in a longer overall survival (OS) of CDX-burdened mice (Figure 6B), a higher ADCY10 expression signified a poorer OS in human LUAD patients (Figure 6C). KH7 is a specific inhibitor that inhibits ADCY10 in various cell types 51. Indeed, treating H1975 cells with KH7 substantially reduced their proliferation (Figure S6G). Exchange proteins respectively activated by cAMP (EPAC) and PKA are two major downstream targets of cAMP. KH7-impaired cell proliferation was rescued by the EPAC-selective activator 8-pCPT; however, it was not rescued by the PKA-specific activator 6-Bnz (Figure S6G). These results demonstrated that, in addition to a role in sensitizing ferroptosis, ADCY10 also promotes EPAC-dependent tumorigenesis.

Due to the importance of ADCY10 in both ferroptosis and tumorigenesis, we wondered whether enhanced vulnerability to ferroptosis benefits LUADs with higher ADCY10 expression. We first evaluated the association between the efficacy of ferroptosis and the expression of ADCY10, and IKE, an erastin analog with high *in vivo* metabolic stability 52, was administrated to CDX mouse models. We found that IKE was ineffective in further reducing tumor growth, increasing MDA concentration or prolonging OS time once upon ADCY10 expression was impaired (Figure 6A-B and Figure S6D-E), suggesting that the expression of ADCY10 is essential for the efficacy of ferroptosis-based therapy *in vivo*.

We also asked, what kind of LUAD patients have high ADCY10 expression levels? Given that ADCY10 was upregulated in a time course-dependent manner not only in 3D spheroids but also in transplanted tumors (Figure S6H), we speculated that ADCY10 is highly expressed in LUADs with advanced stage. As expected, the upregulation of ADCY10 was associated with tumor stage in human LUADs (Figure 6D). Do LUAD patients in advanced stages benefit from ferroptosis induction? To answer this question, we established two primary LUAD cell lines derived from stage I and III patients. Hereafter, these cell lines are termed as primary LUAD cell-I (PLUADC-I) and PLUADC-III. Compared to the PLUADC-I cells, the PLUADC-III cells with higher ADCY10 expression were more sensitive to erastin-induced reduction in cell viability and suppression of GFPT1 and YAP (Figure 6E and Figure S6I). We also found that, compared to PLUAD-I, PLUAD-III was resistant to routine chemotherapeutic drugs including gemcitabine, paclitaxel and Cisplatin (Figure S6J). Fortunately, erastin concentrations as low as 1 μM exhibited a strong killing effect on PLUAD-III cells (Figure 6E). However, erastin at concentrations as high as 10 μM, did not kill bronchial epithelial BEAS-2B cells or lung fibroblast MRC-5 cells (Figure 6F), suggesting that ferroptosis-based therapy is a relatively safe strategy to treat LUAD. PDX mouse models are useful tools to evaluate drug effects. PDX-I and PDX-III models were constructed from LUAD cells derived from patients in stages I and III. Tumors in relative higher ADCY10-expressing PDX-III models grew faster than those in relative lower ADCY10-expressing PDX-I models; however, IKE inhibited tumor growth and prolonged the OS time of PDX-III mice to a greater extent than it did in the PDX-I mice. These effects were found to be related to ferroptotisis because they were blocked by Lipro-1, a stable *in vivo* ferroptosis inhibitor (Figure 6G-H). We also found that PLUADC-III cells were more invasive and displayed more vascular co-option in the lung than the PLUADC-I cells (Figure 6I-K), and these effects were largely inhibited by IKE (Figure 6L-N). Overall, ferroptosis-related treatment is a good choice for treating high ADCY10-expressing LUAD patients, such as those in advanced-stage ones.

Then, we evaluated whether therapy resistance alone resulted in a higher ADCY10 expression and increased sensitivity to ferroprosis. Cisplatin resistance of A549 cells caused the elevated expression of ADCY10 and increased sensitivity to both erastin and Sorafenib (Figure 6O-P). In addition, higher ADCY10 expression was exhibited in a LUAD patient after failure of treatment of Icotinib, a first-generation epidermal growth factor receptor-tyrosine kinase inhibitor (EGFR-TKI) (Figure 6Q), suggesting that patients who fail targeted therapy exhibit higher expression of ADCY10. Indeed, resistance of AZD9291, another third-generation EGFR-TKI in H1975 and HCC827 cells also acquired a higher ADCY10 expression and more sensitivity to erastin and Sorafenib (Figure 6R-S), suggesting that ferroptosis-based therapy might benefit for those LUAD patients with targeted therapy resistance. As high therapy-resistant mesenchymal high status confers increased sensitivity to ferroptosis 17, we wondered whether ADCY10 drives epithelial-mesenchymal transition (EMT). Knocking down ADCY10 in H1975 and HCC827 cells caused a significant increase in E-cadherin level and reductions in N-cadherin, Snail and Vimentin (Figure 6T), demonstrating that inducing the EMT might be another function of ADCY10 that may make LUAD cells more sensitive to ferroptosis.

### Ferroptosis sensitivity varies among LUAD cells following a YAP suppression-dependent labile iron elevation

**Figure 7:**
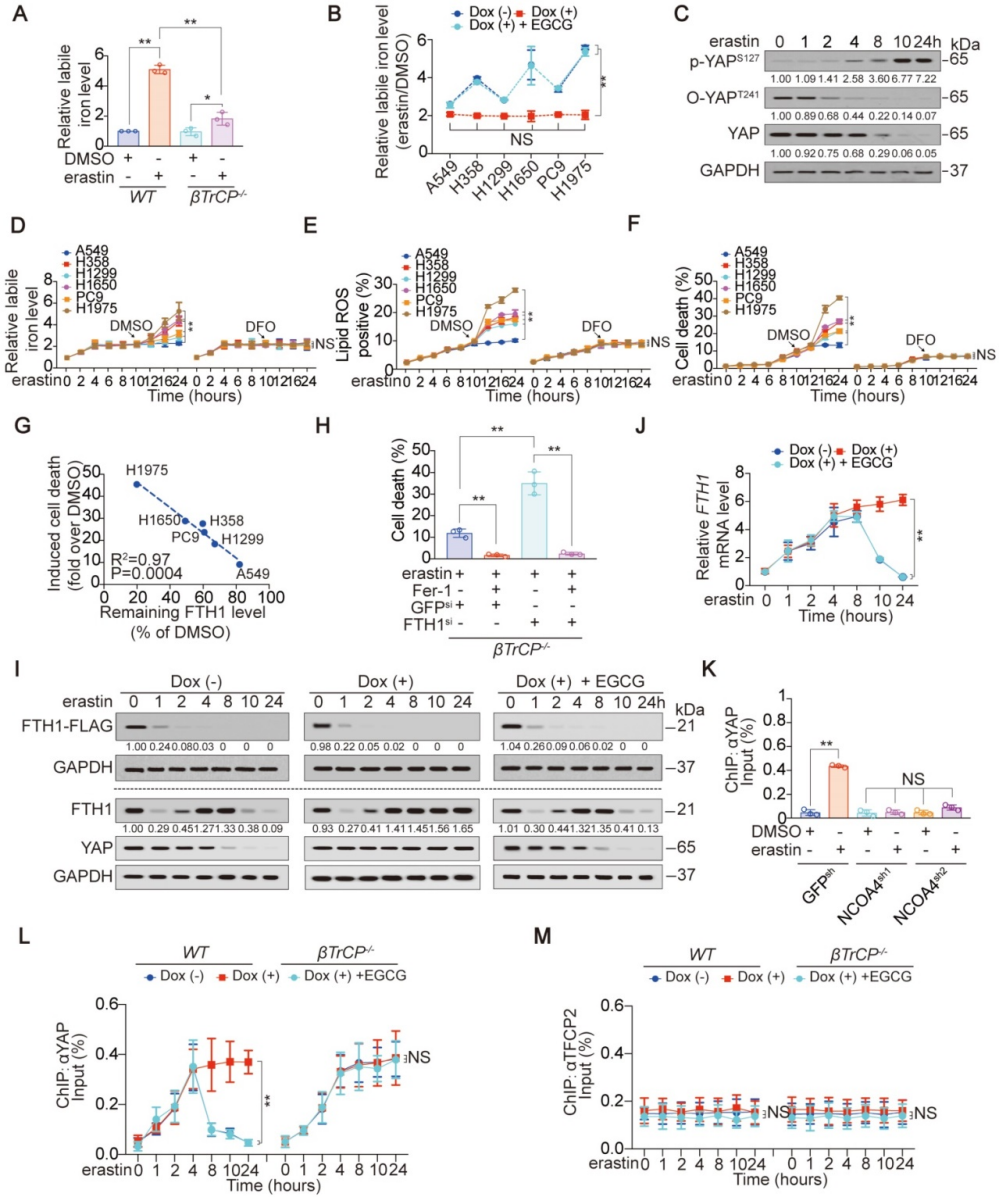
** Reduction of YAP sensitizes ferroptosis by elevating iron via FTH1. (A)** Relative labile iron was measured in* WT* and *βTrCP^-/-^* H1975 cells after treating with or without erastin (10 µM) for 24 h. **(B)** Relative labile iron was measured in GGG cells under the same treatment as that in Figure 2D. The labile iron was normalized between those treated with erastin and DMSO. **(C)** p-YAP^S127^, O-YAP^T241^ and total YAP were measured by IB in H1975 cells treated with erastin (10 µM) for indicated hours. **(D-F)** Relative labile iron (D), lipid ROS generation (E) and cell death (F) were measured in LUAD cells treated with erastin (10 µM) for indicated hours. DMSO or DFO (A549, 2 µM; H358,15 µM; H1299, 5 µM; H1650, 18 µM; PC9, 8 µM; H1975, 25 µM) was added at 10 h post erastin treatment. **(G)** Correlation between induced cell death and remaining FTH1 level after erastin (10 µM) treatment for 24 h. Induced cell death and remaining FTH1 level was calculated as fold or percentage to the ones treated with DMSO. **(H)** FTH1 was knocked down in *βTrCP^-/-^* H1975 cells treating with erastin (10 µM) with or without Fer-1 (1 µM) for 24 h. Cell death was measured using SYTOX green staining followed by flow cytometry. **(I-J)** FTH1 protein (I) and mRNA (J) in H1975-based GGG cells pretreated with or without Dox (1 µg/ml) and EGCG (5 µM) for 24 h before further treating with erastin (10 µM) for indicated hours. The level of proteins was normalized to that of GAPDH, and the normalized level of proteins in erastin-treated 0 h cells was arbitrarily set to 1 (I). **(K)** Ferritinophagy boosts YAP binding to *FTH1* promoter. H1975 cells with or without NCOA4 knockdown were treated with erastin (10 µM) for 4 h. Enrichments of YAP at TFCP2 motif within the *FTH1* promoter were measured by ChIP-qPCR. **(L-M)** Dynamic YAP and TFCP2 binding to the *FTH1* promoter triggered by erastin. *WT* or *βTrCP^-/-^* H1975-based GGG cells were under the same treatment as that in panel I. Enrichments of YAP (L) or TFCP2 (M) at TFCP2 motif within the *FTH1* promoter were measured by ChIP-qPCR at indicated hours. The data are shown as the mean ± SD from three biological replicates (including IB). *P < 0.05, **P < 0.01 indicates statistical significance. Data in A, H, K were analyzed using a one-way ANOVA test. Data in B, J, L, M were analyzed using a two-way ANOVA test. Data in G were analyzed using the Spearman rank-correlation analysis.

Although the suppression of YAP following system X_C_^-^ inhibition is determined by glutamate level and the ADCY10-dependent inhibition of GFPT1 (Figure 1-5), the downstream mechanism by which YAP suppression modulates ferroptotic circumstance is unknown. Compared to that in parental cells, the prevention of YAP suppression in *βTrCP^-/-^* H1975 cells did not influence the erastin-induced reduction in GSH levels (Figure S7A). βTrCP deletion also failed to affect AA or AdA (Figure S7B-C); however, erastin-induced increase in labile iron was significantly reduced (Figure 7A). The trend of labile iron elevation was contrary to that YAP was suppressed, and such was also glutamate-dependent (Figure 2I and 7B). Thus, YAP suppression might facilitate labile iron elevation following system X_C_^-^ inhibition.

Via time course measurements, we found that p-GFPT1^S205^ was rapidly induced at 0.5 h following erastin treatment and maintained at high levels for up to 4 h in H1975 cells. In contrast, a substantial decrease in GFPT1 was observed starting at 1 h and was maintained at a low level thereafter (Figure S7D). O-YAP^T241^, p-YAP^S127^ and total YAP levels were reduced or induced in succession after the alteration of GFPT1 (Figure 7C). An obvious YAP decrease was observed 8 h after treatment with erastin in H1975 cells (Figure 7C), leading us to investigate whether the ferroptotic responses of LUAD cells vary after that time. As expected, a varied secondary increase in labile iron in LUAD cells was observed beginning at 10 h after erastin treatment (Figure 7D). Lipid ROS generation was also observed at 10 h (Figure 7E), which might be because the iron-mediated Fenton reaction enhances lipid peroxidation at that time. The secondary enhanced erastin-induced cell death and -reduced cell viability were detected ~2 h later (at 12 h) (Figure 7F and Figure S7E). To further investigate whether the varied ferroptotic responses were outcomes following secondary elevation of labile iron, we blocked labile iron increases by DFO at 10 h after treatment with erastin (Figure 7D). As expected, the variations in ferroptotic responses were diminished upon the addition of DFO (Figure 7E-F and Figure S7E). These results demonstrated that ferroptosis sensitivity is determined by YAP suppression at later stages via elevating a varied secondary labile iron in LUAD cells following system X_C_^-^ inhibition.

### YAP suppression leads to the failure of transcriptionally sustained ferritin at later stages following system X_C_^-^ inhibition

By proteomics analysis, which is shown in Figure 1G, we found that iron metabolism-associated proteins, including FTH1, FTL1 and IREB2, were reduced, while TFRC, TF and CISD1 were increased following the erastin treatment of H1975 cells (Figure S7F). Among these proteins, only the degree to which FTH1 (the heavy subunit of ferritin) suppressed were varied between A549 and H1975 cells (Figure S7G). We also evaluated other iron metabolism-related proteins that were not detected by the proteomics, i.e., NCOA4, FPN1, HSPB1, NRF2, PHKG2 and DMT1, and found that none of them changed differentially between A549 and H1975 cells (Figure S7H). Therefore, we focused on FTH1 thereafter. Similar to YAP, the remaining FTH1 level following erastin treatment was negatively correlated with the extent of ferroptosis (Figure 1I and 7G), suggesting that YAP suppression-promoted ferroptosis depends on FTH1. Indeed, knocking down FTH1 by siRNA resensitized *βTrCP*^-/-^ H1975 cells to erastin-induced ferroptosis when YAP suppression was blocked (Figure 7H and Figure S7I).

Next, we investigated the mode by which YAP regulates FTH1 following system X_C_^-^ inhibition. Erastin-triggered iron release is a result of ferritinophagy 6. Even when glutamate was depleted, exogenous FTH1 protein was still abolished within 2 h upon erastin treatment in H1975 cells (Figure 7I), indicating that ferritinophagy did occur in a glutamate-independent manner. However, the trend of endogenous FTH1 protein alteration was completely different. Due to ferritinophagy, endogenous FTH1 declined within the 1^st^ hour (Figure 7I). Excessive iron feedback induces *FTH1* gene transcription 7, thus explaining why *FTH1* mRNA showed a compensatory increase and the FTH1 protein was maintained from the 2^nd^ h until the 8^th^ hour (Figure 7I-J). Unfortunately, the FTH1 protein level was not sustained by its mRNA thus far 2 h later (~10^th^ hour) after YAP was significant decreased in a glutamate-dependent manner (Figure 7I-J). These results demonstrated that FTH1 is transcriptionally stimulated by YAP following the inhibition of system X_C_^-^ at an early stage but cannot be sustained at a late stage.

Subsequently, we explored how YAP transcriptionally regulates FTH1 following the inhibition of system X_C_^-^. YAP acts as a coactivator to stimulate the activity of transcription factors (TFs), such as TEA domain family members (TEAD) and TFCP2 28. The dynamic changes in TFCP2-based luciferase activity were more obvious than those of TEAD, which changed earlier than the changes in FTH1 expression (Figure 7I-J and Figure S7J), indicating that YAP regulation of FTH1 is TFCP2-dependent. With FIMO software (http://meme-suite.org/doc/fimo.html), a TFCP2 consensus motif (-157~-148 bp relative to the transcription start site) was predicted to be located within the *FTH1* promoter (Figure S7K). The binding between TFCP2 and the *FTH1* promoter was subsequently verified by EMSA and ChIP experiments (Figure S7L-M). Without an intact TFCP2 motif, YAP failed to reinforce TFCP2-induced *FTH1* promoter activity (Figure S7N). These results indicate that TFCP2 is fundamental for YAP to control *FTH1* transcription. Notably, treatment with erastin or Sorafenib enabled YAP to bind with the TFCP2 motif within the *FTH1* promoter in a dose-dependent manner (Figure S7O-P). NCOA4 is a cargo receptor involved in FTH1 degradation by ferritinophagy following system X_C_^-^ inhibition 53. NCOA4 depletion diminished the erastin-induced YAP recruitment to the *FTH1* promoter in H1975 cells (Figure 7K and Figure S7Q), indicating that ferritinophagy triggers the YAP-dependent transcriptional compensation of FTH1. Finally, we examined whether YAP suppression at later stages leads to the dissociation of YAP from the *FTH1* promoter following system X_C_^-^ inhibition. Although YAP recruitment to the *FTH1* promoter was sustainably increased during the first 4 hours following erastin treatment, it began to decrease thereafter following YAP suppression in a glutamate-dependent manner (Figure 7L). These effects were consistent with the decrease in YAP; however, these effects were diminished in *βTrCP*^-/-^ H1975 cells (Figure 7C, 7I and 7L). Moreover, TFCP2 enrichment at the *FTH1* promoter was not affected (Figure 7M), demonstrating that the effects of YAP are specific. Overall, YAP suppression fails to transcriptionally sustain ferritin at later stages.

## Discussion

System X_C_^-^ imports extracellular cystine in exchange for intracellular glutamate 10. Although cystine uptake is inhibited following the inhibition of system X_C_^-^, the effects of accumulating endogenous glutamate have not been fully elucidated. Prior studies have reported that the addition of exogenous glutamate at a high concentration can also inhibit system X_C_^-^ and trigger ferroptosis via the disruption of the concentration gradient between extracellular and intracellular glutamate 54, 55. In this study, although accumulated endogenous glutamate following inhibition of system X_C_^-^ was identified as an indicator of the degree of ferroptosis, it was found to be insufficient to trigger ferroptosis. Therefore, the functions of the “exogenous” and “endogenous” glutamate are diverse, at least for the initiation and progression of ferroptosis.

**Figure 8:**
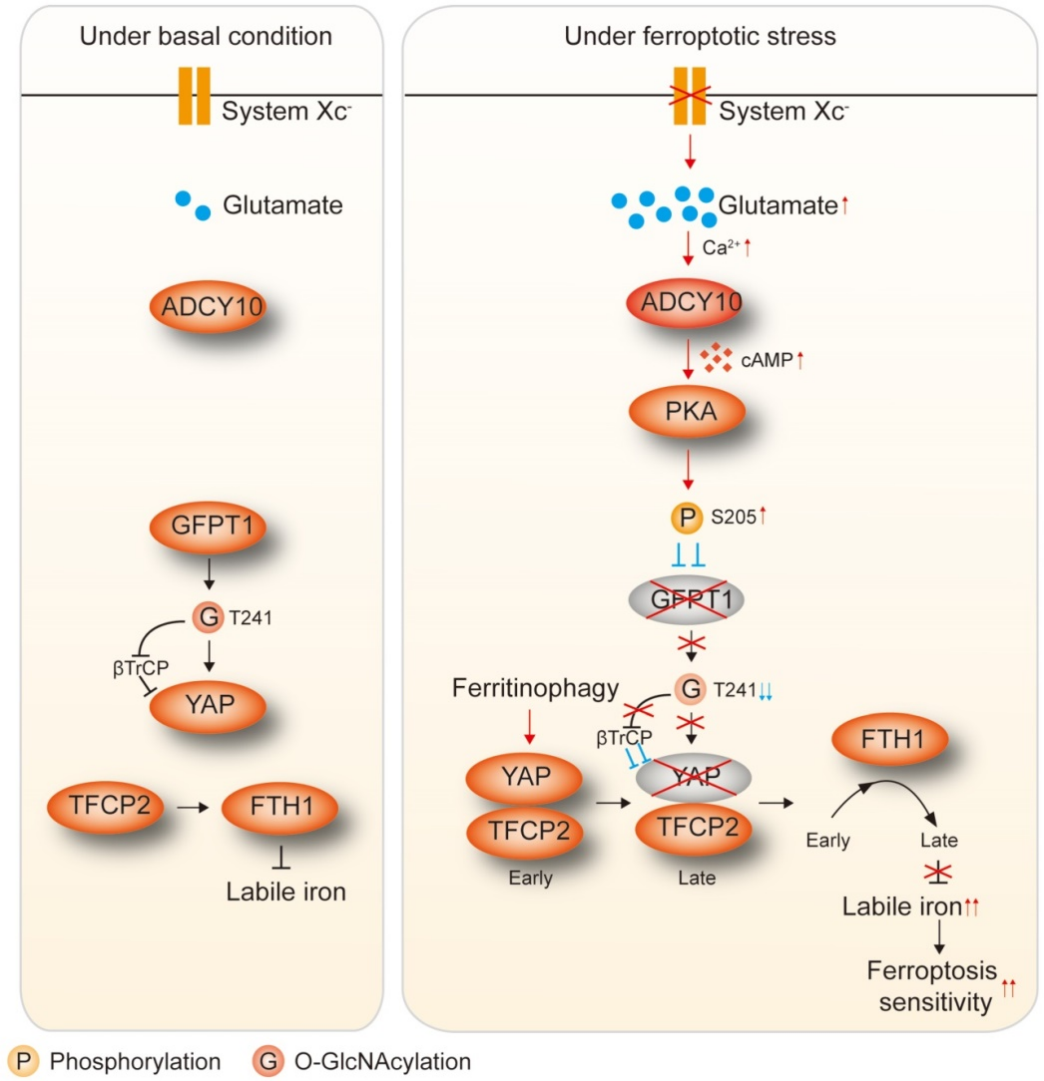
** The model of the study.** Under basal condition, GFPT1 maintains O-GlcNAcylation of YAP to prevent YAP from Hippo pathway-dependent suppression, such like βTrCP-mediated ubiquitination. Meanwhile, basal transcription of FTH1 is controlled by TFCP2 to regulate iron homeostasis. When system X_C_^-^ is inhibited, endogenous glutamate is accumulated, by which promotes Ca^2+^-dependent cAMP production by ADCY10 to stimulate PKA-associated phosphorylation and suppression of GFPT1. Subsequently, YAP is inevitably suppressed and fail to sustain ferritinophagy-triggered transcriptional compensatory of FTH1 at later stage. This also leads to a varied labile iron elevation and ferroptosis sensitivity among LUAD cells.

To the best of our knowledge, we are the first to reveal that glutamate accumulation determines ferroptosis sensitivity by stimulating Ca^2+^-dependent activation of ADCY10 in LUAD cells. ADCY10 acts as a key downstream target to produce cAMP and diversifies the impacts of glutamate to trigger cAMP and PKA-dependent phosphorylation and suppression of GFPT1. Subsequently, the O-GlcNAcylation and stability of YAP are reduced. We also found that ferritinophagy-induced YAP recruitment to the TFCP2 motif within the *FTH1* promoter is indispensable for compensator ferritin increases. However, the remarkable decline in YAP failed to sustain ferritin at the later stage. Labile iron was then elevated, finally sensitizing LUAD cells to ferroptosis (Figure 8). On the basis of our cell-based and preclinical models, we also revealed that the responses to the induction of ferroptosis depend on the existing intracellular ADCY10 expression; thus merely measuring ADCY10 expression is a promising method to evaluate whether the LUAD patients are sensitive to the ferroptosis-based therapy. Moreover, ADCY10 is elevated at advanced stages and also when therapy resistance is evident. Given that ferroptosis sensitivity is correlated with the level of ADCY10, ferroptosis-based treatment might be beneficial for advanced-stage and therapy-resistant LUAD patients.

Ferroptosis sensitivity varies widely in cells and tissues and may be related to the expression of certain factors, such as p53 11. However, the mechanism by which ferroptosis is augmented in LUAD cells remains largely unknown. In addition to its tumorigenic function, YAP was recently shown to promote ferroptotic environments in cancer cells by stimulating TEAD-based transcription of Acyl-CoA synthetase long chain family member 4 (ACSL4) and TFRC 17, which are both critical ferroptotic modulators 4, 56. A ferroptotic environment can be disrupted by the activation of the Hippo pathway 17. Interestingly, we reveal here that the Hippo pathway suppression of YAP has another role in augmenting ferroptosis in LUAD cells following the inhibition of system X_C_^-^. This finding is not contradictory to the prior study 17 because the TEAD-based transcription program stimulated by YAP is a prerequisite for triggering ferroptosis, while the degree of ferroptosis is determined by the remaining YAP levels to compensate for TFCP2-based ferritin transcription. Hence, YAP is critical to modulate both the initiation and progression of ferroptosis. In addition, our findings indicating that YAP strongly declined 8 h following system X_C_^-^ inhibition, explains, to a certain extent, why apparent ferroptosis occurs at similar time points upon treatment with erastin 1.

XBP1s is critical for sustaining GFPT1 under ER stress 39. However, our findings demonstrated that ineffective splicing of XBP1 facilitates GFPT1 degradation following the inhibition of system X_C_^-^ because of inefficient phosphorylation and activation of IRE1α, which processes XBP1 transcripts coding XBP1s upon ER stress 57. Low basal levels and delayed induction of IRE1α in the lung might also account for the ineffective retention of GFPT1 58. IRE1α does not increase significantly until stimulation under ER stress for at least 12 h in the lung 58; however, our data showed that rapid and robust suppression of GFPT1 only requires 4 h. Even if it is possible for XBP1 splicing to compensate for GFPT1 after 12 h, it is too late to prevent impaired YAP O-GlcNAcylation and ferroptosis. The reported phosphorylation sites within GFPT1 include Ser205, Ser235 and Ser243 24, 45. The phosphorylation of Ser205 and Ser235 is PKA-dependent, and the phosphorylation of Ser205 also inhibits GFPT1 activity 24; however, only Ser205 is phosphorylated in LUAD cells, which might be due to a LUAD cell-specific effect. mTORC2 signaling regulates phosphorylation at Ser243 45. Unfortunately, our data also exclude phosphorylation at Ser243 following system X_C_^-^ inhibition. Posttranslational modification is critical for protein homeostasis 59. However, how GFPT1 is degraded following phosphorylation remains unknown and needs to be investigated in the future.

EPAC and PKA are two major downstream targets of cAMP, and they can be generated by ADCY10 60. When cells transition from benign to malignant, the upregulation of ADCY10 stimulates cell growth in an EPAC-dependent manner 60. Our findings also showed that ADCY10 exerts its protumorigenic roles in LUAD cells via EPAC. However, the augmentation of ferroptosis by ADCY10 is related to PKA. These results suggest that the tumorigenic and ferroptotic functions of ADCY10 are separate. ADCY10 is increased with tumor progression and therapy resistance, indicating that advanced-stage and therapy-resistant LUADs are more susceptible to ferroptosis. The EMT boosts metastasis, which is another major characteristic of tumor progression 61, and indeed, cancer cells with EMT and metastatic properties are highly sensitive to ferroptosis 17. The fact that ADCY10 is an EMT-promoting protein in LUAD cells further supports that increasing ADCY10 can sensitize LUAD cells to ferroptosis. Although ferroptosis has antitumor effects, the example of ADCY10 emphasizes that the contributor to promote tumorigenesis might also be the determinant of the increased ferroptosis sensitivity of LUAD cells.

The alteration of FTH1 following ferroptosis initiation is dynamic. Through a 24 h monitoring, the changes in FTH1 were shown to involve initial downregulation, secondary upregulation and final downregulation. Although this action is influenced by multiple signals, YAP participates in every process. The initial downregulation is due to the rapid onset of ferritinophagy 6, 62. During this stage, YAP is recruited to the *FTH1* promoter where it can initiate transcriptional compensation for the loss of ferritin. The secondary upregulation of FTH1 can also be regarded as a feedback response to the increase in labile iron 7. A study revealed that NRF2 and FTH1 are both increased upon erastin treatment of hepatocellular carcinoma cells, suggesting that NRF2 stimulates *FTH1* transcription upon ferroptosis 63. However, considering our findings, we suggest that YAP is the key regulator that transcriptionally sustains FTH1 in LUAD cells. Failure to induce *FTH1* transcription following YAP suppression at later stages eventually leads to a secondary increase in labile iron, the level of which varies among LUAD cells and determines the degree of ferroptosis. These effects rely on the coordination among the ADCY10/PKA, HBP and Hippo pathways, suggesting that ferroptosis cannot be achieved by a single signaling pathway.

In conclusion, we emphasize the importance of accumulated endogenous glutamate to augment ferroptosis following the inhibition of system X_C_^-^. The degree to which YAP is suppressed by the ADCY10/PKA/GFPT1 signaling axis determines the iron-dependent ferroptosis sensitivity of LUAD cells. ADCY10 simultaneously exerts tumorigenic and ferroptotic effects in LUAD; therefore, patients with elevated ADCY10 expression are more likely to benefit from ferroptosis-based therapy. Ferroptosis might be a good choice for cancer treatment, and clinical trials will be conducted based on ferroptosis in the near future. We are confident that there will be additional attractive factors that can serve as biomarkers for the prediction of both the prognosis and ferroptosis sensitivity, or as the targets for ferroptosis themselves.

## Supplementary Material

Supplementary figures and tables.

## Abbreviations

6-Bnz: N^6^-Benzoyladenosine-3',5'-cyclic monophosphate
8-pCPT-cAMP: 8-(4-Chlorophenylthio)-2'-O-methyladenosine 3',5'-cyclic monophosphate
A549-C^Res^: A549-based Cisplatin resistant
AA: arachidonic acid
ACSL4: Acyl-CoA synthetase long chain family member 4
AdA: adrenic acid
ADCY10: adenylate cyclase 10
ALB: albumin
AOA: aminooxyacetic acid
BME: basement membrane extract
CA12: carbonic anhydrase 12
cAMP: cyclic adenosine 3′,5′-monophosphate
cAMP: cyclic adenosine monophosphate
CD31: cluster of differentiation 31
CDX: cell-derived xenograft
CHX: cycloheximide
ChIP: chromatin immunoprecipitation
CISD1: CDGSH iron sulfur domain 1
co-IP: co-immunoprecipitation
DFO: deferoxamine
DMEM: Dulbecco's modified eagle medium
DMK: dimethyl alpha ketoglutarate
DMT1: divalent metal transporter 1
DPBS: Dulbecco's phosphate buffered saline
E-cadherin: epithelial cadherin
EGCG: epigallocatechol gallate
EGFR-TKI: epidermal growth factor receptor-tyrosine inhibitor
EGTA: ethylenebis (oxyethylenenitrilo) tetraacetic acid
ELISA: enzyme linked immunosorbent assay
EM: electronic microscope
EMSA: electrophoretic mobility shift assay
EMT: epithelial-mesenchymal transition
EPAC: exchange proteins activated by cAMP
ER: endoplasmic reticulum
F-6-P: fructose-6-phosphate
FHL2: four and a half LIM domains 2
FPN1: ferroportin-1
FTH1: ferritin heavy chain 1
FTL1: ferritin light chain 1
GAPDH: glyceraldehyde-3-phosphate dehydrogenase
GDH: glutamate dehydrogenase
GFPT1: glutamine-fructose-6-phosphate transaminase
GLAST: glutamate aspartate transporter
GlcN: glucosamine
GlcN-6-P: glucosamine-6-P
GlcNAc: N-acetylglucosamine
GlcNAc-1-P: N-acetylglucosamine-1-phosphate
GLS: glutaminase
GLT1: glutamate transporter 1
GLUD1: glutamate dehydrogenase 1
GOT2: glutamic-oxaloacetic transaminase 2
GPT2: glutamic pyruvate transaminase
GPX4: glutathione peroxidase 4
GSH: glutathione
H1975-A^Res^: H1975-based AZD9291 resistant
HBA1: hemoglobin subunit alpha 1
HBD: hemoglobin subunit delta
HBP: hexosamine biosynthesis pathway
HBSS: Hank's balanced salt solution
HCC827-A^Res^: HCC827-based AZD9291 resistant
HSPB1: heat shock protein family B (small) member 1
IB: immunoblotting
IF: immunofluorescence
IHC: immunohistochemistry
IKE: imidazole ketone erastin
IRE1α: inositol-requiring protein 1alpha
IREB2: iron responsive element binding protein 2
K48-Ub: lysine 48-linked polyubiquitin chain
KRT81: keratin 81
LUAD: lung adenocarcinoma
Lipro-1: liproxstatin-1
LUC: luciferase
MDA: malondialdehyde
MT2A: metallothionein 2A
NAC: Acetyl-L-cysteine
N-cadherin: nerual cadherin
NCOA4: nuclear receptor coactivator 4
NFS1: cysteine desulfurase
NRF2: nuclear factor, erythroid 2 like 2
O-GlcNAcylation: O-linked beta-N-acetylglucosaminylation
O-YAP^T241^: threonine 241 O-GlcNAcylated YAP
PDX: patient-derived tumor xenograft
PDX: patient-derived xenograft
PHKG2: phosphorylase kinase catalytic subunit gamma 2
PI: propidium Iodide
PKA: protein kinase A
PKI: PKA inhibitor
PLUADC: primary LUAD cell
PTGS2: prostaglandin-endoperoxide synthase 2
p-YAP^S127^: serine 127 phosphorylated YAP
qPCR: quantitative RT-PCR
ROS: reactive oxygen species
Rp-cAMPS: Rp-Adenosine 3',5'-cyclic monophosphorothioate
SCD1: stearoyl-Coenzyme A desaturase 1
SLC1A4: solute carrier family 1 member 4
SLC3A2: solute carrier family 3 members
SLC7A11: solute carrier family 7 member 11
Smurf1: SMAD specific E3 ubiquitin protein ligase 1
TEAD: TEA domain family mambers
TF: transferrin
TFCP2: transcription factor CP2
TFRC: transferrin receptor
TFs: transcription factors
TMT: tandem mass tags
Ub: ubiquitin
UDP-GlcNAc: UDP-N-acetylglucosamine
VIM: vimentin
VTN: vitronectin
WCL: whole cell lysates
WT: wild type
XBP1: X-box-binding protein 1
YAP: yes-associated protein
α-KG: alpha-ketoglutaric acid
β-ME: β-mercaptoethanol
βTrCP: beta-transducin repeat containing E3 ubiquitin protein ligase
