# Supplementary material for: Endogenous glutamate determines ferroptosis sensitivity via ADCY10-dependent YAP suppression in lung adenocarcinoma
Source: Theranostics. 2021 Mar 24;11(12):5650–74. doi: 10.7150/thno.55482 (PMC8058707; doi:10.7150/thno.55482)

Supplementary Figure S1

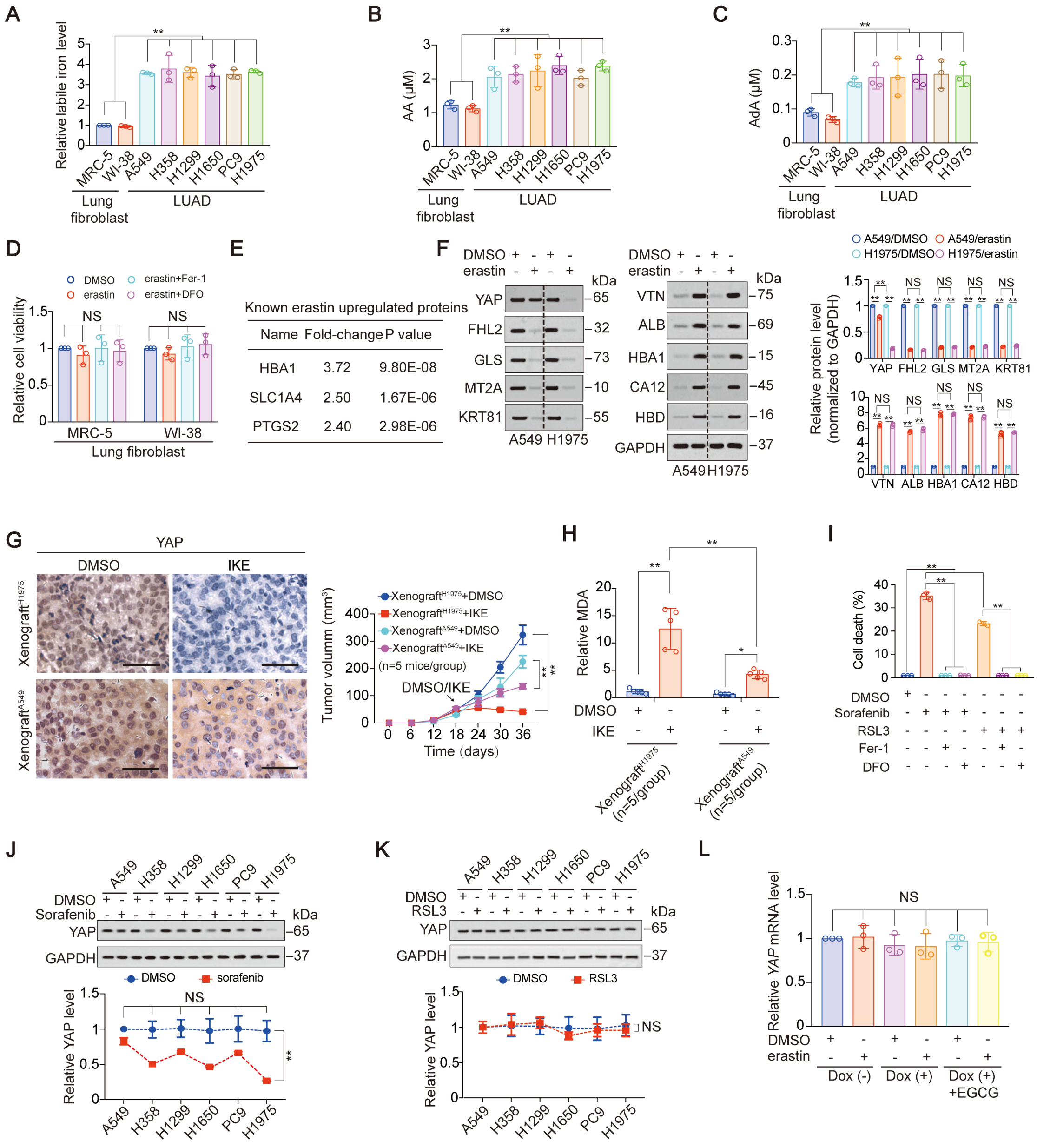

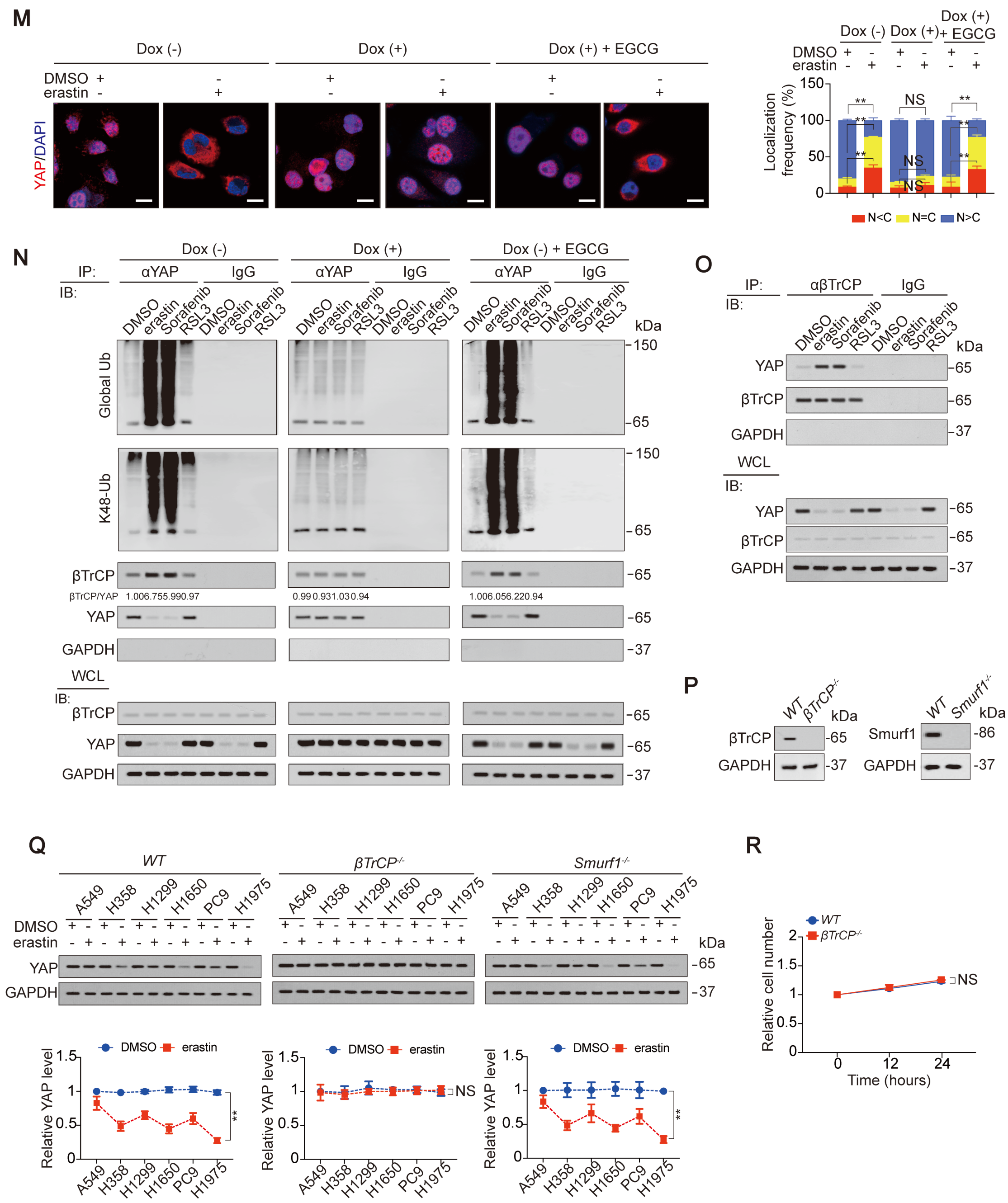

Supplementary Figure S1. Ferroptosis sensitivity is determined by reduction of YAP.

(A-C) Relative labile iron (A), AA (B) and AdA (C) were measured in indicated lung fibroblast or LUAD cells.

(D) Cell viability was measured in MRC-5 and WI-38 cells treating with erastin (10 μM) with or without Fer-1 (1 μM) or DFO (80 μM) for 24 h.

(E) Known erastin upregulated proteins those were also elevated in erastin-treated H1975 cells, as revealed by TMT.

(F) Indicated proteins in A549 and H1975 cells, as measured by IB after

treating with or without erastin (10 μM) for 24 h. The level of proteins was normalized to that of GAPDH and graphed on the right.
(G) Representative YAP IHC image for xenografts formed by H1975 or A549 cells. DMSO or IKE (50 mg/kg) was administrated at day 18 after inoculation. Tumor volume was monitored for another 18 days, (scale bar, 200 μm).
(H) MDA was examined in xenografts at day 36 with the same treatment as those in panel G.
(I) Cell death was measured by SYTOX green staining followed by flow cytometry in H1975 cells with Sorafenib (5 μM) or RSL3 (5 μM) treatment, in the presence or absence of Fer-1 (1 μM) or DFO (80 μM) for 24 h.
(J-K) IB of YAP in LUAD cells treated with Sorafenib (5 μM) (J) or RSL3 (5 μM) (K) for 24 h. The level of YAP was normalized to that of GAPDH, and the normalized level of YAP in DMSO-treated A549 cells was arbitrarily set to 1.
(L) H1975-based GGG cells were pretreated with or without Dox (1 μg/ml) and EGCG (5 μM) for 24 h before further treating with or without erastin (10 μM) for 24 h. Relative YAP mRNA level was measured by qRT-PCR.
(M) H1975-based GGG cells were pretreated with or without Dox (1 μg/ml) and EGCG (5 μM) for 24 h before further treating with erastin (10 μM) for 6 h. Representative IF images of YAP localization were shown on the left, and the localization frequency of YAP was graphed on the right. N > C, YAP is enriched in nucleus; N = C, YAP is evenly distributed in cytoplasm and nucleus; N < C, YAP is enriched in cytoplasm (scale bar, 20 μm)
(N) Co-immunoprecipitation was performed using anti-YAP or anti-IgG antibodies in H1975-based GGG cells pretreated with or without Dox (1 μg/ml) and EGCG (5 μM) for 24 h before further treating with or without erastin (10 μM), Sorafenib (5 μM) or RSL3 (5 μM) for another 8 h. Indicated proteins were identified by IB.
(O) Co-immunoprecipitation was performed using anti-βTrCP or anti-IgG antibodies in H1975 cells treated with or without erastin (10 μM), Sorafenib (5 μM) or RSL3 (5 μM) for 8 h. Indicated proteins were identified by IB.
(P) βTrCP and Smurf1 were measured by IB in *WT*, *βTrCP*$^{-/-}$ and *Smurf1*$^{-/-}$ H1975 cells.
(Q) IB of YAP in *WT*, *βTrCP*$^{-/-}$ or *Smurf1*$^{-/-}$ LUAD cells treated with or without

erastin (10 μM) for 24 h.

(R) Relative cell number was monitored at indicated hours in *WT* and *βTrCP* $^{-/-}$ H1975 cells. The initial cell number was 10^5 per well.

The data are shown as the mean ±SD from three biological replicates (including IB). *P < 0.05, **P < 0.01 indicates statistical significance. Data in A, B, C, D, F, H, I, L, M were analyzed using a one-way ANOVA test. Data in G, J, K, Q, R were analyzed using a two-way ANOVA test. Data in E were analyzed using a Student's t test.

Supplementary Figure S2

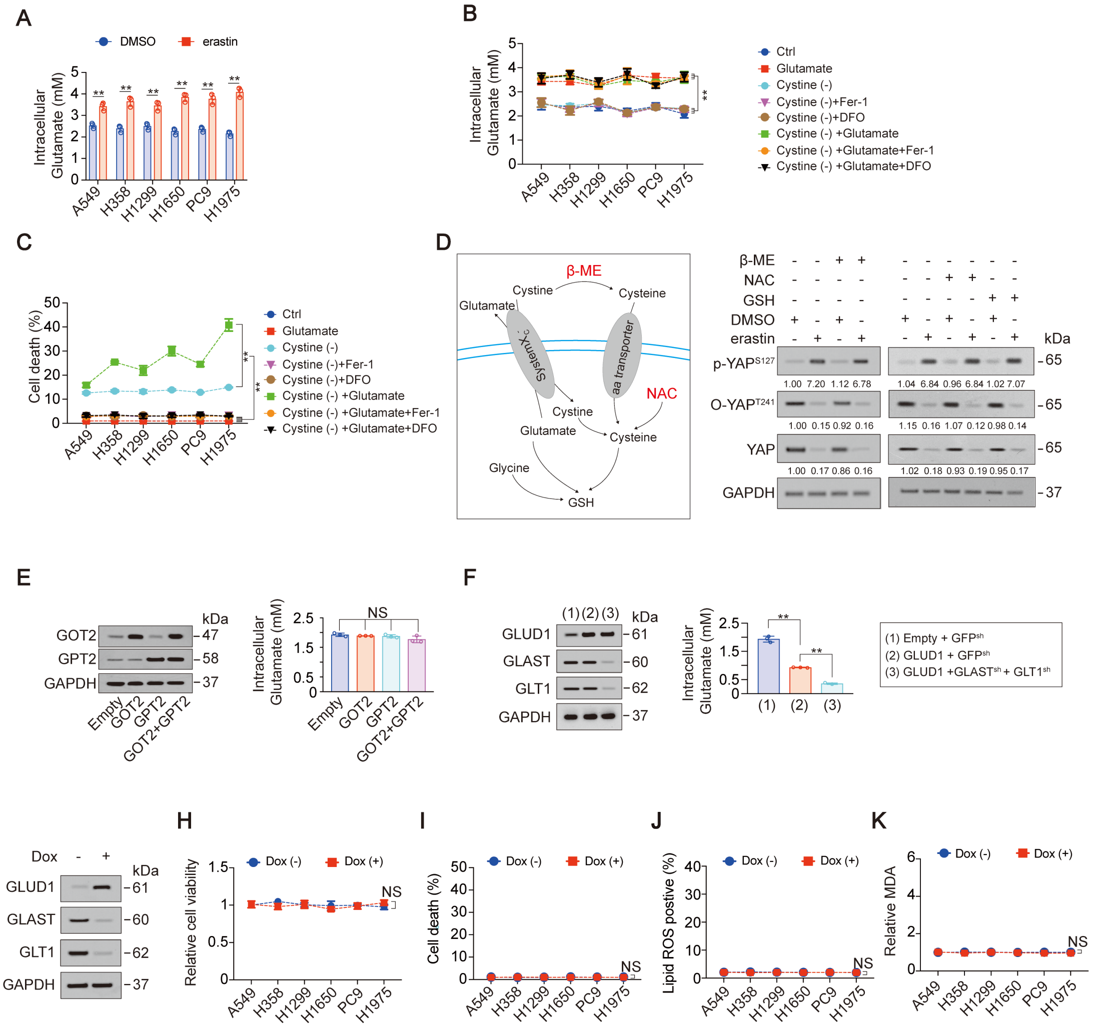

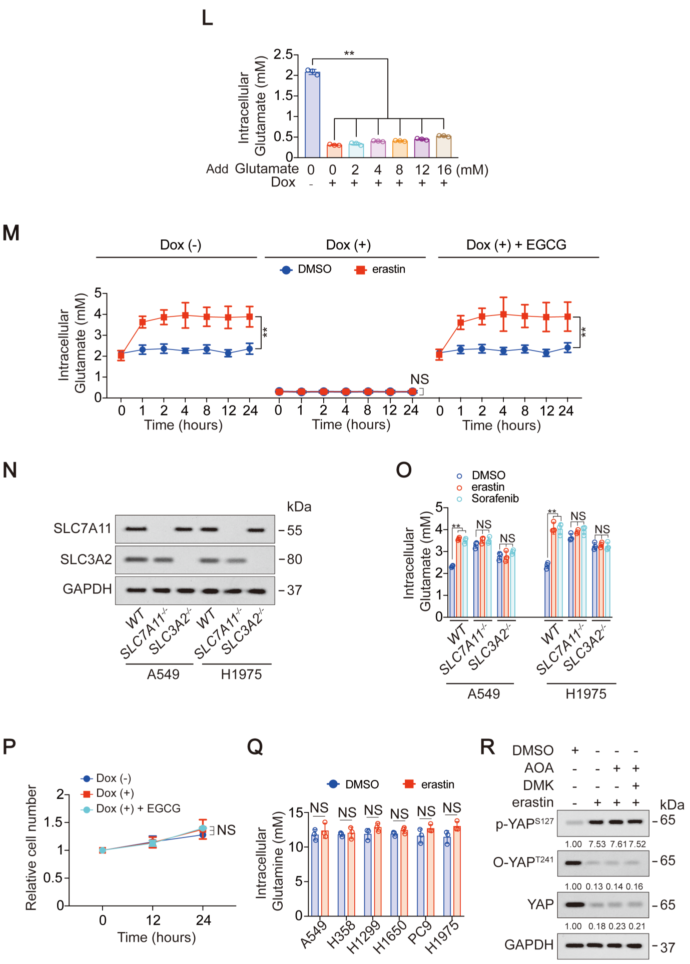

Supplementary Figure S2. Endogenous glutamate suppresses YAP.

(A) Intracellular glutamate was measured in indicated LUAD cells with or without erastin treatment (10 μM) for 24 h.

(B-C) Intracellular glutamate (B) and cell death (C) were measured in LUAD cells under cystine deprivation with or without treatment of glutamate (2 mM), Fer-1 (1 μM) or DFO (80 μM).

(D) Schematic representation shows how β-ME and NAC promotes GSH synthesis. p-YAP^{S127}, O-YAP^{T241} and total YAP were measured by IB in H1975 cells treated with or without erastin (10 μM), in the presence or absence of β-ME (50 μM), NAC (1 mM) or GSH (50 μM) for 8 h. The level of proteins was normalized to that of GAPDH, and the normalized level of proteins in DMSO-treated cells was arbitrarily set to 1.

(E) Overexpressing transaminases has no roles on glutamate. GOT2 and GPT2 were measured by IB in H1975 cells with or without GOT2 or GPT2

overexpression (left). Intracellular glutamate was also measured and graphed (right).

(F) Endogenous glutamate is suppressed by overexpressing GLUD1 with simultaneous knocking GLAST and GLT1 down. GLUD1, GLAST and GLT1 were measured by IB in H1975 cells with indicated treatment (left). Intracellular glutamate was also measured and graphed (right).

(G) GLUD1, GLAST and GLT1 were measured by IB in H1975-based GGG cells with or without Dox (1 μg/ml) treatment for 24 h.

(H-K) Dox has no roles on cell viability, cell death and lipid peroxidation, as evaluating by cellular ATP (H), SYTOX green (I) and C11BODIPY (J) probing followed by flow cytometry, and MDA concentration (K) in LUAD cells after treating with or without Dox (1 μg/ml) for 24 h in LUAD cells.

(L) Adding excessive exogenous glutamate is ineffective to restore endogenous glutamate in Dox-treated H1975-based GGG cells. H1975-based GGG cells were pretreated with or without Dox (1 μg/ml) for 24 h before further treating with exogenous glutamate at indicated concentration for 24 h. Intracellular glutamate was measured and graphed.

(M) Endogenous glutamate is accumulated by erastin. H1975-based GGG cells were pretreated with or without Dox (1 μg/ml) and EGCG (5 μM) for 24 h before further treating with or without erastin (10 μM) for indicated hours.

(N) SLC7A11, SLC3A2 expression was measured in *WT*, *SLC7A11*$^{-/-}$ or *SLC3A2*$^{-/-}$ A549 and H1975 cells.

(O) Intracellular glutamate was measured in *WT*, *SLC7A11*$^{-/-}$ or *SLC3A2*$^{-/-}$ A549 and H1975 cells treated with or without erastin (10 μM) or Sorafenib (5 μM) for 24 h.

(P) Endogenous glutamate has no effect on cell proliferation during the timeframe of our treatment. Relative cell number was monitored at indicated hours in H1975-based GGG cells treated with or without Dox (1 μg/ml) and EGCG (5 μM). The initial cell number was 10^5 per well.

(Q) Intracellular glutamine was measured in indicated LUAD cells with or without erastin treatment (10 μM) for 24 h.

(R) α-KG has no roles on YAP modification and expression. p-YAP^{S127}, O-YAP^{T241} and total YAP were measured by IB in H1975 cells treated with or

without erastin (10 μM), in the presence or absence of AOA (1 mM) and DMK (4 mM) for 8 h. The level of proteins was normalized to that of GAPDH, and the normalized level of proteins in DMSO-treated cells was arbitrarily set to 1. The data are shown as the mean ±SD from three biological replicates (including IB). $^{**}P < 0.01$ indicates statistical significance. Data in A, E, F, L, O, Q were analyzed using a one-way ANOVA test. Data in B, C, H, I, J, K, M, P were analyzed using a two-way ANOVA test.

Supplementary Figure S3

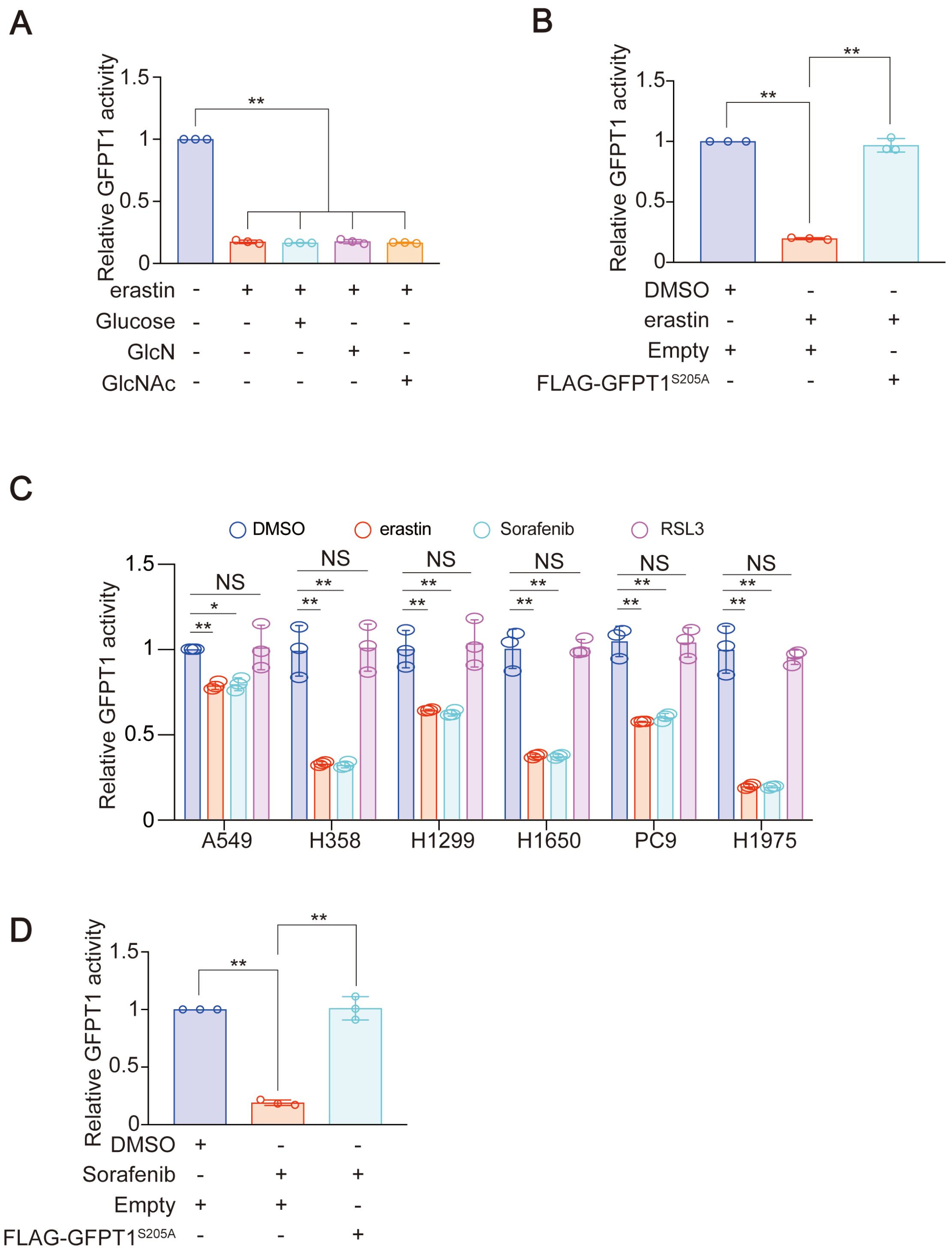

Supplementary Figure S3. GFPT1 activity is decreased by erastin and Sorafenib.

(A) GFPT1 activity was measured using GDH method in H1975 cells treated with or without erastin (10 μM), glucose (25 mM), GlcN (5 mM) or GlcNAc (5 mM) for 8 h.

(B) GFPT1^{S205A} compensates reduced GFPT1 activity by erastin.

FLAG-GFPT1^{S205A} was ectopically expressed in H1975 cells prior to the treatment with or without erastin (10 μM) for 8 h. GFPT1 activity was measured using GDH method.

(C) GFPT1 activity was measured using GDH method in indicated LUAD cells treated with or without erastin (10 μM), Sorafenib (5 μM), or RSL3 (5 μM) for 8 h.

(D) FLAG-GFPT1^{S205A} was ectopically expressed in H1975 cells prior to the treatment with or without Sorafenib (5 μM) for 8 h. GFPT1 activity was measured using GDH method.

The data are shown as the mean ±SD from three biological replicates. *P < 0.05, **P < 0.01 indicates statistical significance. All the data in were analyzed using a one-way ANOVA test.

Supplementary Figure S4

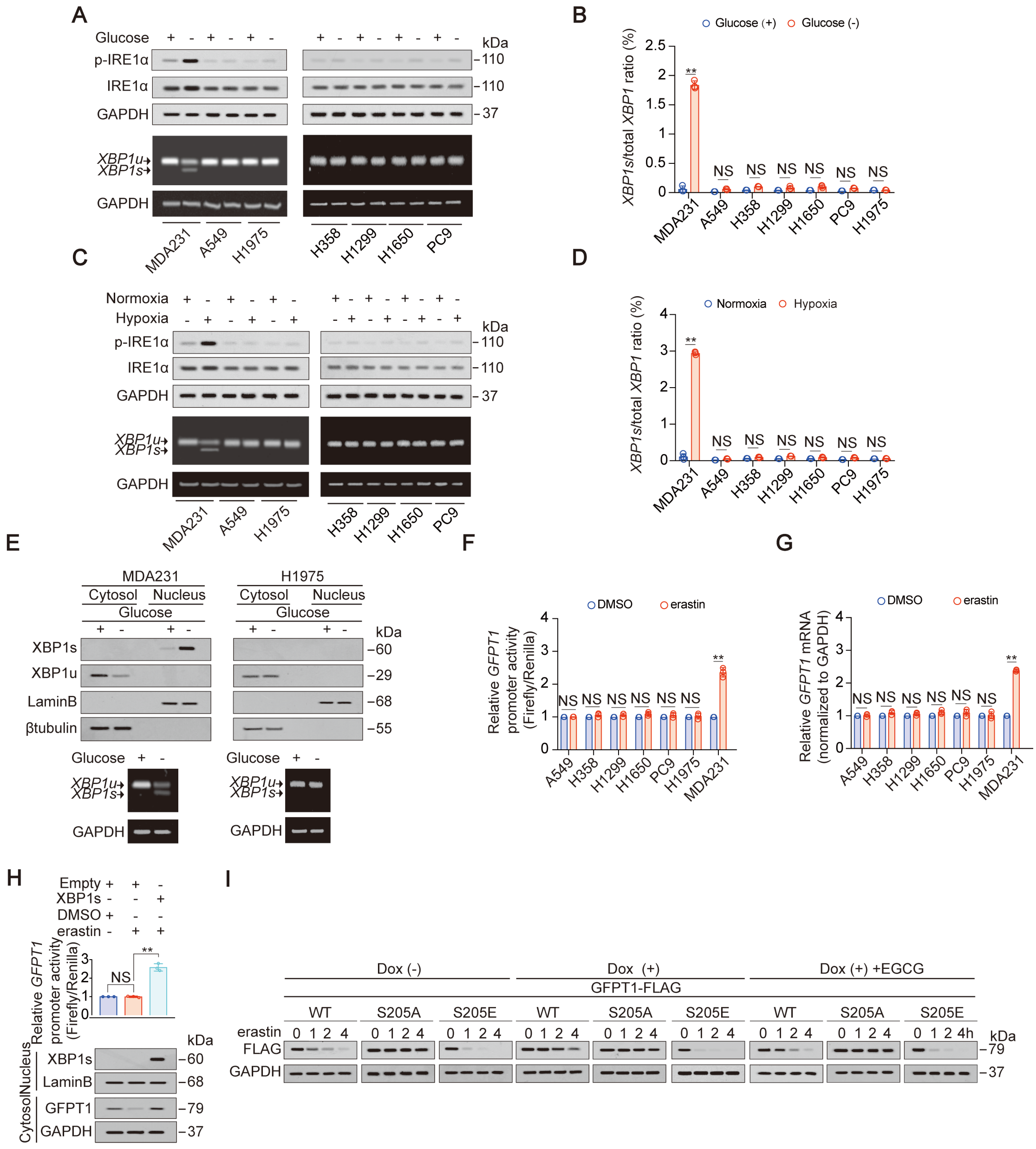

Supplementary Figure 4. Splicing of XBP1 is ineffective in LUAD cells under stress.

(A) Phosphorylated or total IRE1α and spliced (s) or unspliced (u) form of *XBP1* mRNA in MDA-MB-231 and indicated LUAD cells were treated with or without glucose deprivation for 12 h. Protein and mRNA were measured by IB and semi-RT-qPCR via agarose gel electrophoresis, respectively.

(B) *XBP1s*/total *XBP1* mRNA ratio in MDA-MB-231 and indicated LUAD cells under the same treatment as that in panel A.

(C) Phosphorylated or total IRE1α and spliced or unspliced form of *XBP1* mRNA in MDA-MB-231 and indicated LUAD cells treated with normoxia or

hypoxia condition (0.1% O_2) for 12 h.

(D) *XBP1s*/total *XBP1* mRNA ratio in MDA-MB-231 and indicated LUAD cells under the same treatment as that in panel C.

(E) Spliced or unspliced form of XBP1 proteins in nuclear and cytosolic fractions of MDA-MB-231 and H1975 cells treated with or without glucose deprivation for 12 h. Spliced or unspliced form of *XBP1* mRNAs in the cell lysates were simultaneously measured.

(F-G) *GFPT1* promoter activity (F) and mRNA level (G) were analyzed by Dual-luciferase assay and qPCR in MDA-MB-231 and indicated LUAD cells with or without erastin (10 μM) treatment for 8 h.

(H) H1975 cells were ectopically expressed with XBP1s before further treating with or without erastin (10 μM) for 8 h. *GFPT1* promoter activity was analyzed by a Dual-luciferase assay (firefly luciferase activity was normalized to that of renilla). Indicated proteins in nuclear and cytosolic fractions were analyzed by IB.

(I) Indicated GFPT1-FLAG was ectopically expressed and measured by IB in H1975-based GGG cells pretreating with or without Dox and EGCG for 24 h before further treating with erastin (10 μM) for indicated hours.

The data are shown as the mean ±SD from three biological replicates (including IB and agarose gel electrophoresis). **$P < 0.01$ indicates statistical significance. Data in B, D, F, G, H were analyzed using a one-way ANOVA test.

Supplementary Figure S5

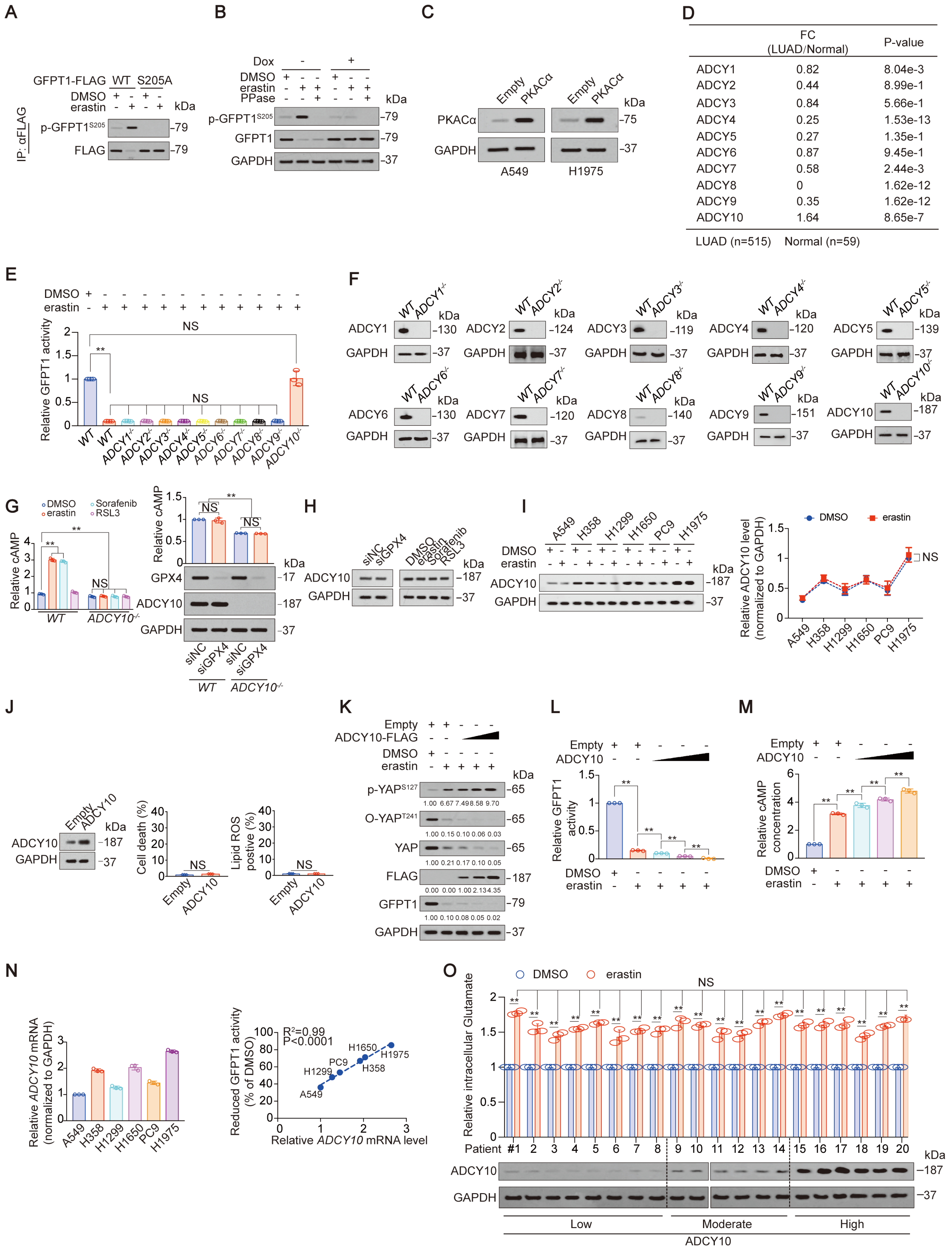

Supplementary Figure S5. ADCY10 determines ferroptosis sensitivity in LUAD cells.

(A) Co-immunoprecipitation was performed using anti-FLAG antibodies in H1975 cells expressing WT- or S205A-GFPT1-FLAG treated with or without erastin (10 μM) for 4 h. Indicated proteins were identified by IB.

(B) Cell lysates extracted from H1975-based GGG cells pretreated with or without Dox for 24 h before further treating with or without erastin (10 μM) for 4 h were incubated with or without PPase for 30 min. p-GFPT1^{S205} and total GFPT1 were measured by IB.
(C) PKACα overexpressed in A549 and H1975 cells as measured by IB.
(D) UALCAN database was used to analyze alterations of ADCY1-10 expression between LUAD (n = 515) and normal lung (n = 59) tissues.
(E) Relative GFPT1 activities were measured in *WT* or in *ADCY1*$^{-/-}$-*10*$^{-/-}$ H1975 cells with or without erastin (10 μM) treatment for 8 h.
(F) Indicated ADCY-proteins were measured by IB in *WT* or in *ADCY1*$^{-/-}$-*10*$^{-/-}$ H1975 cells.
(G) cAMP was measured in control and H1975 cells with ADCY10 knockout transfected with control siRNA or siRNA targeting against GPX4, or treated with or without erastin (10 μM), Sorafenib (5 μM) or RSL3 (5 μM) for 8 h.
(H) ADCY10 expression was measured in H1975 cells transfected with control siRNA or siRNA targeting against GPX4, or treated with or without erastin (10 μM), Sorafenib (5 μM) or RSL3 (5 μM) for 24 h.
(I) ADCY10 protein level in indicated LUAD cells treated with or without erastin (10 μM) for 24 h, as measured by IB. ADCY10 level was normalized to that of GAPDH and graphed on the right. The normalized level of ADCY10 in DMSO-treated A549 cells was arbitrarily set to 1.
(J) Cell death and Lipid ROS generation were measured in H1975 cells with or without ADCY10 overexpression.
(K-M) H1975 cells transfected with empty plasmids or increasing concentration of ADCY10-expressing plasmids (1-4 μg/well) were further treated with or without erastin (10 μM) for 8 h. Indicated proteins, GFPT1 activity and cAMP were measured by IB (K), GDH assay (L) and a cAMP determination kit (M), respectively.
(N) *ADCY10* mRNA was measured in indicated LUAD cells by qPCR, and its correlation with reduced GFPT1 activity (calculated as percentage of erastin treating group to the ones treated with DMSO) was analyzed using the Spearman rank-correlation analysis.
(O) Intracellular glutamate was measured in the same specimens as those in

Figure 5N after treating with DMSO or erastin (10 μM) for 24 h.

The data are shown as the mean ±SD from three biological replicates (including IB). **P < 0.01 indicates statistical significance. Data in E, G, L, M, O were analyzed using a one-way ANOVA test. Data in D, J were analyzed using a Student's t test. Data in I were analyzed using a two-way ANOVA test. Data in N were analyzed using the Spearman rank-correlation analysis.

Supplementary Figure S6

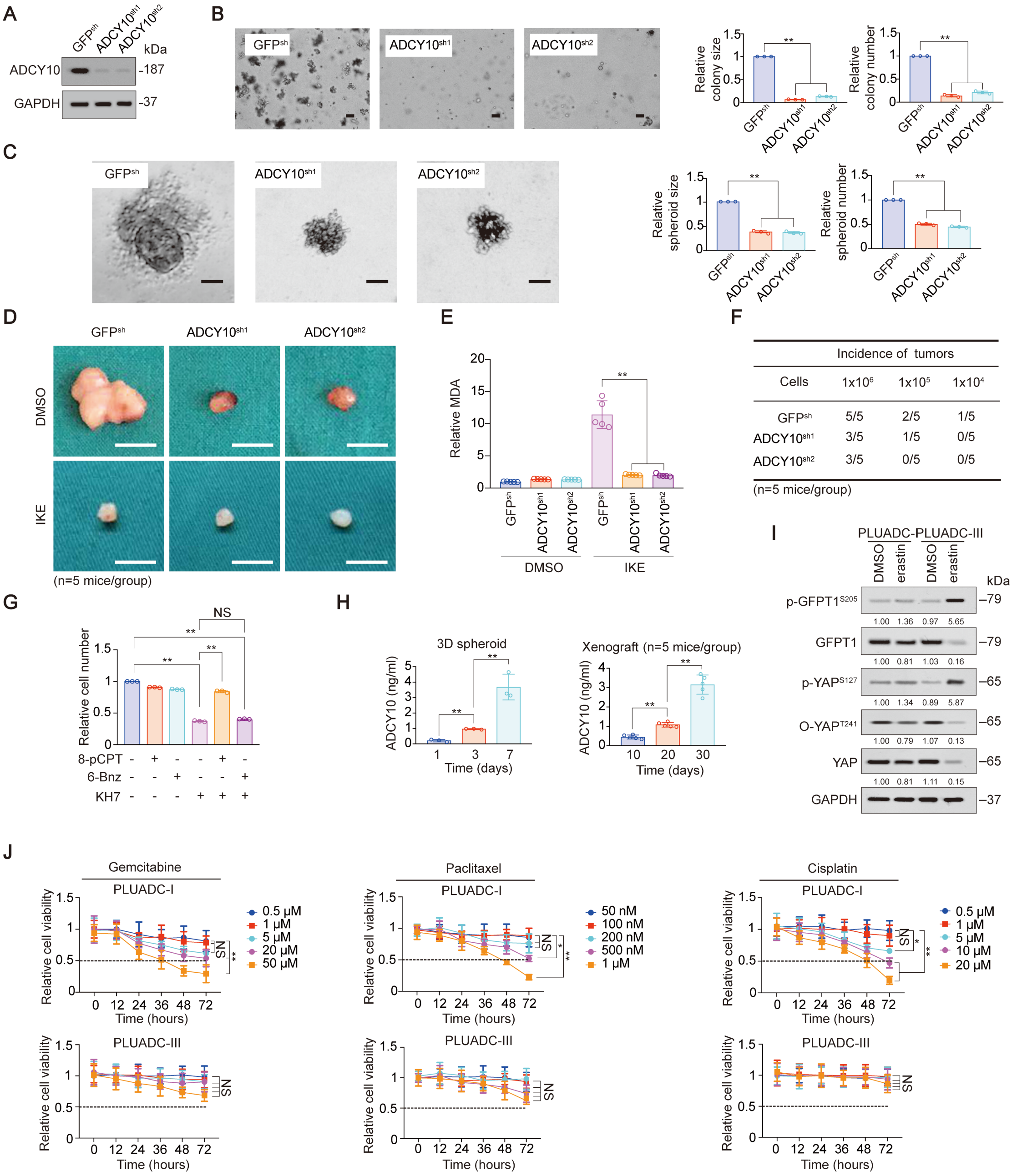

Supplementary Figure S6. The protumorigenic and proferroptotic roles of ADCY10 in LUAD cells.

(A) ADCY10 was measured by IB in H1975 cells with or without ADCY10 knockdown.

(B) Colony formation was analyzed by anchorage-independent soft agar assay in H1975 cells with or without ADCY10 knockdown. Relative colony size and amounts (Φ >30 μm) were counted and graphed on the right. Scale bar, 50 μm.

(C) 3D spheroids generated from H1975 cells with or without ADCY10 knockdown. Relative spheroid size and amounts (Φ >30 μm) were counted and

graphed on the right. Scale bar, 50 μm.

(D) Representative images of xenografts that formed at the endpoint of the experiments which were the same as those in Figure 6A. Scale bar, 5 mm.

(E) MDA was measured in xenografts which were formed in Figure 6A and Supplementary Figure 6D (n = 5/group).

(F) The indicated number of H1975 cells with or without ADCY10 knockdown were injected into athymic mice and the tumor incidence reported at 12-week post-transplantation.

(G) Relative cell number was measured in H1975 cells treating with 8-pCPT (100 μM), 6-Bnz (100 μM) or KH7 (20 μM) for 24 h. The initial cell number was 10^5 per well.

(H) ADCY10 levels in 3D spheroids or xenografts were measured at the indicated day post plating or implantation.

(I) Indicated proteins were measured by IB in PLUADC-I/III cells with or without erastin (10 μM) treatment for 8 h. The level of proteins was normalized to that of GAPDH, and the normalized level of proteins in DMSO-treated cells was arbitrarily set to 1.

(J) Relative cell viability was measured in PLUADC-I/III cells treating with gemcitabine, paclitaxel or cisplatin at indicated concentrations for indicated hours.

The data are shown as the mean ±SD from three biological replicates (including IB). **P < 0.01 indicates statistical significance. Data in B, C, E, G, H were analyzed using a one-way ANOVA test. Data in I were analyzed using a two-way ANOVA test.

Supplementary Figure S7

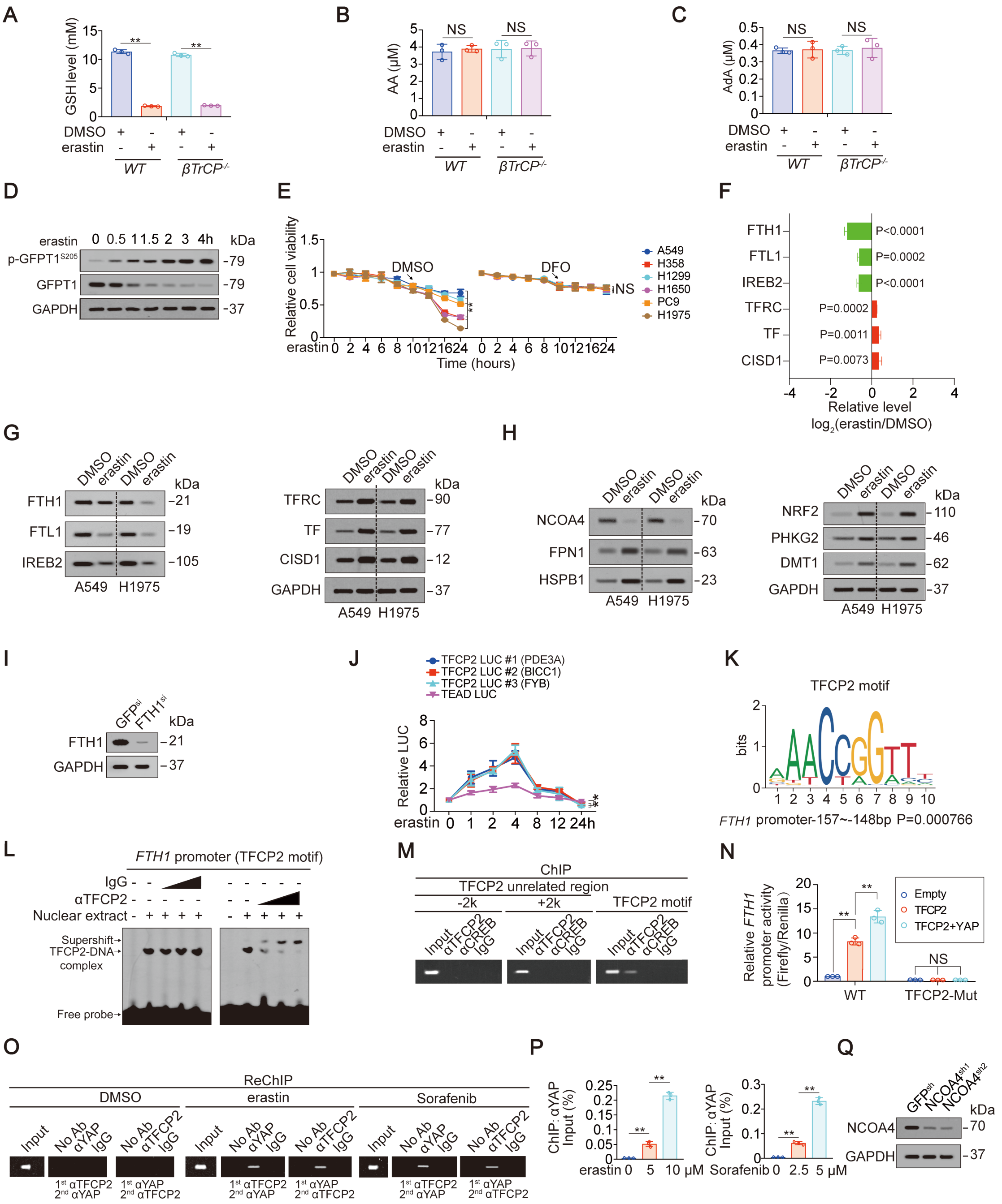

Supplementary Figure S7. YAP compensates FTH1 by regulating its transcription following inhibition of system X_C^-.

(A-C) GSH (A), AA (B) and AdA (C) were measured in *WT* and *βTrCP-/-* H1975 cells after treating with or without erastin (10 μM) for 24 h.

(D) p-GFPT1^{S205} and total GFPT1 were measured in H1975 cells by IB after erastin (10 μM) treatment for indicated hours.

(E) Relative cell viability was measured in LUAD cells treated with erastin (10 μM) for indicated hours, DMSO or DFO (A549, 2 μM; H358,15 μM; H1299, 5

μM; H1650, 18 μM; PC9, 8 μM; H1975, 25 μM) was added at 10 h post erastin treatment.

(F) Alterations of iron-related proteins in H1975 cells treated with DMSO or erastin (10 μM) for 24 h, as revealed by TMT.

(G-H) Iron-related protein level in A549 and H1975 cells treated with DMSO or erastin (10 μM) for 24 h, as validated by IB.

(I) FTH1 knockdown efficiency in H1975 cells transfected with or without siRNA targeting against FTH1.

(J) Luciferase from 3 TFCP2-based and 1 TEAD-based reporters were measured in H1975 cells post erastin (10 μM) treatment for indicated hours using a Dual-luciferase assay.

(K) A TFCP2 motif (image from JASPAR database) was identified within human *FTH1* promoter (-157~-148 bp relative to TSS) as predicted using FIMO software.

(L) Nuclear extracts from H1975 cells were incubated with anti-IgG or anti-TFCP2 antibodies for 15min prior to further incubating with biotin-labeled probes containing TFCP2 motif from *FTH1* promoter. The TFCP2-DNA complexes and supershifts were measured using EMSA.

(M) Enrichments of TFCP2 but not CREB at TFCP2 motif within the *FTH1* promoter were measured in H1975 cells using ChIP followed by agarose gel electrophoresis. Regions at -2k/+2k were used as negative controls.

(N) The luciferase activities of *FTH1* promoter with or without TFCP2 motif mutation were measured in H1975 cells ectopically expressed with TFCP2 and YAP.

(O) Co-occupancy of YAP and TFCP2 within the *FTH1* promoter were measured in H1975 cells with or without erastin (10 μM) or Sorafenib (5 μM) treatment for 4 h using Re-ChIP followed by agarose gel electrophoresis.

(P) Treating erastin (0-10 μM) and Sorafenib (0-5 μM) for 4 h recruited YAP onto the *FTH1* promoter in H1975 cells, as measured by ChIP-qPCR.

(Q) NCOA4 knockdown efficiency in H1975 cells transfected with or without shRNAs targeting against NCOA4.

The data are shown as the mean ±SD from three biological replicates (including IB and agarose gel electrophoresis). **P < 0.01 indicates statistical

significance. Data in A, B, C, N, P were analyzed using a one-way ANOVA test. Data in J were analyzed using a two-way ANOVA test. Data in F were analyzed using a Student's t test.

Supplementary Table S1. Sequence of primers, siRNA, sgRNA and probes for EMSA were listed.

Primers used for qPCR

Name	5'-3'
YAP-qPCR-F	CCTCGTTTTGCCATGAACCAG
YAP-qPCR-R	GTTCTTGCTGTTTCAGCCGCAG
GAPDH-qPCR-F	ATCATCCCTGCCTCTACTGG
GAPDH-qPCR-R	GTCAGGTCCACCACTGACAC
FTH1-qPCR-F	CCTCCTACGTTTACCTGTCCATG
FTH1-qPCR-R	GTCTGGTTTCTTGATATCCTGAAGG
XBP1-qPCR-F	AGGAGTTAAGACAGCGCTTGGGG
XBP1-qPCR-R	CTGAATCTGAAGAGTCAATACCG
XBP1s-qPCR-F	AGGAGTTAAGACAGCGCTTGGGG
XBP1s-qPCR-R	AATACCTGCACCTGCTGCGGACTCAGCAGA
ADCY10-qPCR-F	CTGAGGTCTGGCTTTTCCTCAGC
ADCY10-qPCR-R	GCTGAGGAAAAGCCAGACCTCAG

Primers used for ChIP-qPCR

Name	5'-3'

FTH1-(-2k)-ChIP-F	AGCTAATGAGGTGTCTCTATGCTGTCTC
FTH1-(-2k)-ChIP-R	GCACTCCAGCCTGGAAGACAAAAC
FTH1-(TFCP2)-ChIP-F	CTCCTCGCAGCTTCCCTCCAACTCC
FTH1-(TFCP2)-ChIP-R	TCGCGCGCTCTGGCGGA
FTH1-(2k)-ChIP-F	CACTGGCTGTAAGTTTTCTGTGCAG
FTH1-(2k)-ChIP-R	TCAAAGCACAGCATGAAGGAATC

Primers used for promoter analysis

Name	5'-3'
WT-FTH1-promoter-F	ATGCGGTACCAGCTAATGAGGTGTCTCTATGCTGTC
WT-FTH1-promoter-R	ATGCCTCGAGAGCCGCGTCGGCGTCAGGCCCGCCC
Mut-FTH1-promoter-F	CCGCTCCCGAGCTCCGCCAGAGCGC
Mut-FTH1-promoter-R	GGAGCTCGGGAGCGGCCGACTGCCTCTGGGACAGCGGTGG
WT-GFPT1-promoter-F	GCCGGGTACCTATTTAGACAAATTCTAAAAGACCTG
WT-GFPT1-promoter-R	AATAATAATAGCTAGCGCCGCCGGGCCCCGCCCCT

siRNA sequence

Name	5'-3'
si-GPX4	CAGGGAGUAACGAAGAGAUTT

si-FTH1	CCUUCAGGAUAUCAAGAAATT

sgRNA sequence

Name	5'-3'
ADCY1-sg-F	CACCGGTGCGCAGCCTGCTGGCCAT
ADCY1-sg-R	AAACATGGCCAGCAGGCTGCGCACC
ADCY2-sg-F	CACCGGCCTCTTCTCGTTGGTGATA
ADCY2-sg-R	AAACTATCACCAACGAGAAGAGGCC
ADCY3-sg-F	CACCGGTCCAATTCCAGCCACGGCG
ADCY3-sg-R	AAACCGCCGTGGCTGGAATTGGACC
ADCY4-sg-F	CACCGGCTTATGCTGTGGAGGACGC
ADCY4-sg-R	AAACGCGTCCTCCACAGCATAAGCC
ADCY5-sg-F	CACCGGCCGCGCCTGGATGCACTCT
ADCY5-sg-R	AAACAGAGTGCATCCAGGCGCGGCC
ADCY6-sg-F	CACCGGCAGCATGAGAATCGGCAGC
ADCY6-sg-R	AAACGCTGCCGATTCTCATGCTGCC
ADCY7-sg-F	CACCGGCTGCTCACGCTCCTGCTGG
ADCY7-sg-R	AAACCCAGCAGGAGCGTGAGCAGCC
ADCY8-sg-F	CACCGGCTGCAGGTCATCCTCCAAG

ADCY8-sg-R	AAACCTTGGAGGATGACCTGCAGCC
ADCY9-sg-F	CACCGGAGATGGTGAACATGAGAGT
ADCY9-sg-R	AAACACTCTCATGTTCACCATCTCC
ADCY10-sg-F	CACCGGAAGTCTGTCACCTCACAAG
ADCY10-sg-R	AAACCTTGTGAGGTGACAGACTTCC
SMURF1-sg-F	CACCGGCAGTCCACCACCGAGCCGC
SMURF1-sg-R	AAACGCGGCTCGGTGGTGGACTGCC
βTrCP-sg-F	CACCGGCGAAGGCATGCTGTCCGCC
βTrCP-sg-R	AAACGGCGGACAGCATGCCTTCGCC
GFPT1-sg-F	CACCGGGAGAGAGTTATCCAACAAT
GFPT1-sg-R	AAACATTGTTGGATAACTCTCTCCC
SLC3A2-sg-F	CACCGGGCAGCCGCGGCTAAGTTCA
SLC3A2-sg-R	AAACTGAACTTAGCCGCGGCTGCCC
SLC7A11-sg-F	CACCGGATATCACAGCAGTAGCTGC
SLC7A11-sg-R	AAACGCAGCTACTGCTGTGATATCC

Probes used for EMSA

Name	5'-3'
WT-TF-FTH1-F	AGGCAGTCGGCTACCGGTCCCCGC

WT-TF-FTH1-R	GCGGGGACCGGTAGCCGACTGCCT

Primers used for plasmid construction

Name	5'-3'
GFPT1-WT-FLAG-F	ATGCGGTACCATGTGTGGTATATTTGCTTACTTAA
	ATGCGGATCCTAGCTTGTCATCGTCATCCTTGTAATCATATTC
GFPT1-WT-FLAG-R	CTCACTCTACAGTCACAG
GFPT1-S205A-F'	CCTCTGTTGATTGGTGTACGGAGTG
GFPT1-S205A-R'	ACCAATCAACAGAGGCGCACCTCGCCTTGTGCCAACTGCT
GFPT1-S205E-F'	CCTCTGTTGATTGGTGTACGGAGTG
GFPT1-S205E-R'	ACCAATCAACAGAGGCTCACCTCGCCTTGTGCCAACTGCT
GFPT1-S235A-F'	TGCAATCTCTCTCGTGTGGACAGCA
GFPT1-S235A-R'	ACGAGAGAGATTGCACGCTCCTTTCTTGTCTTTGCCTGTT
GFPT1-S243A-F'	ACAACCTGCCTTTTCCCGGTGGAAG
GFPT1-S243A-R'	GAAAAGGCAGGTTGTCGCGTCCACACGAGAGAGATTGCAG
iGLUD1-F	ATGCGAATTCATGTACCGCTACCTGGGCGAAGC
iGLUD1-R	ATCGGGATCCCTATGTGAAGGTCACACCAGCTTC

Supplementary Document. Certificates of short tandem repeat analysis for cell lines used in the present study.

MRC-5

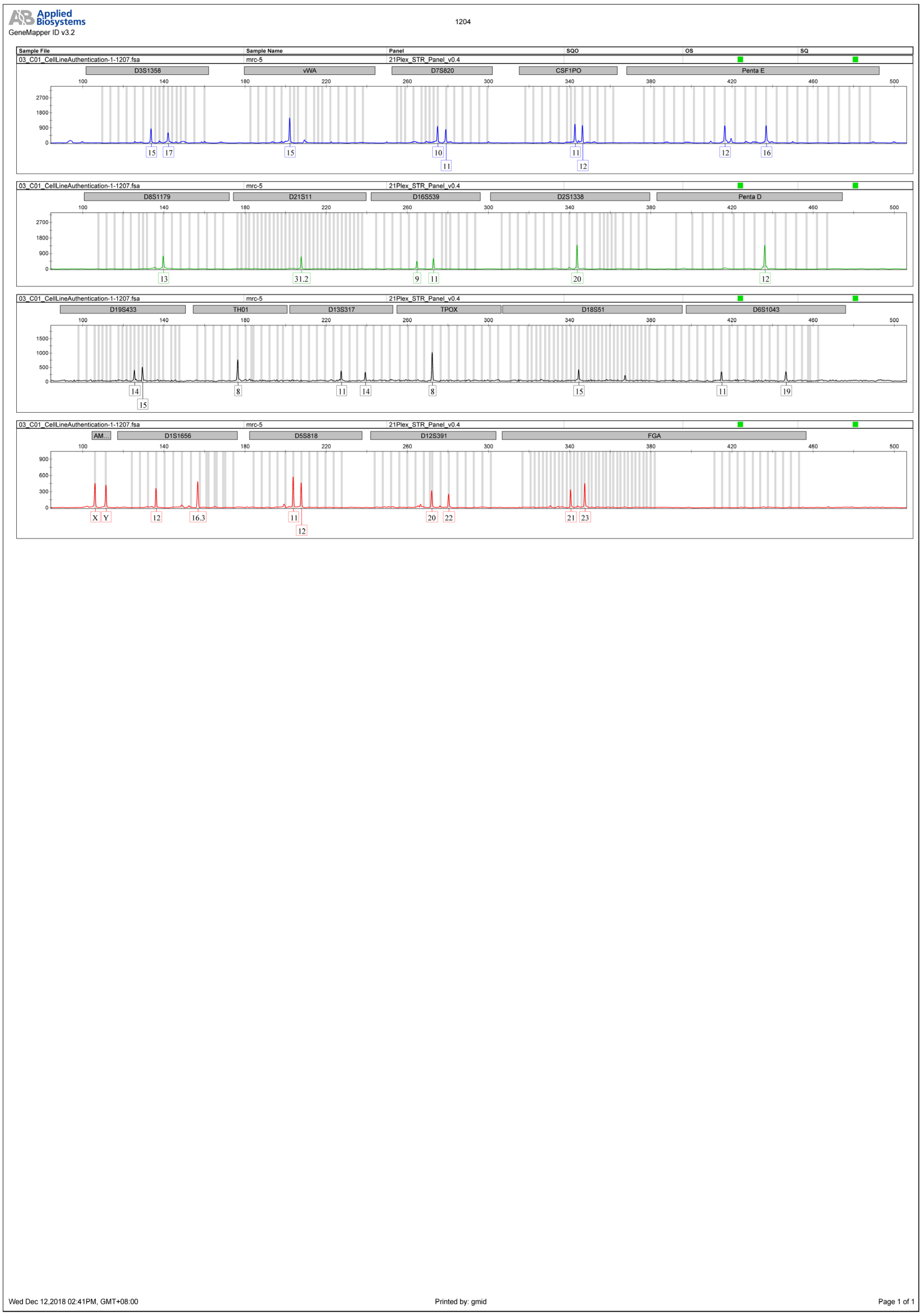

WI38

Applied Biosystems
GeneMapper 4.0

Cell Line Authenticaion-10

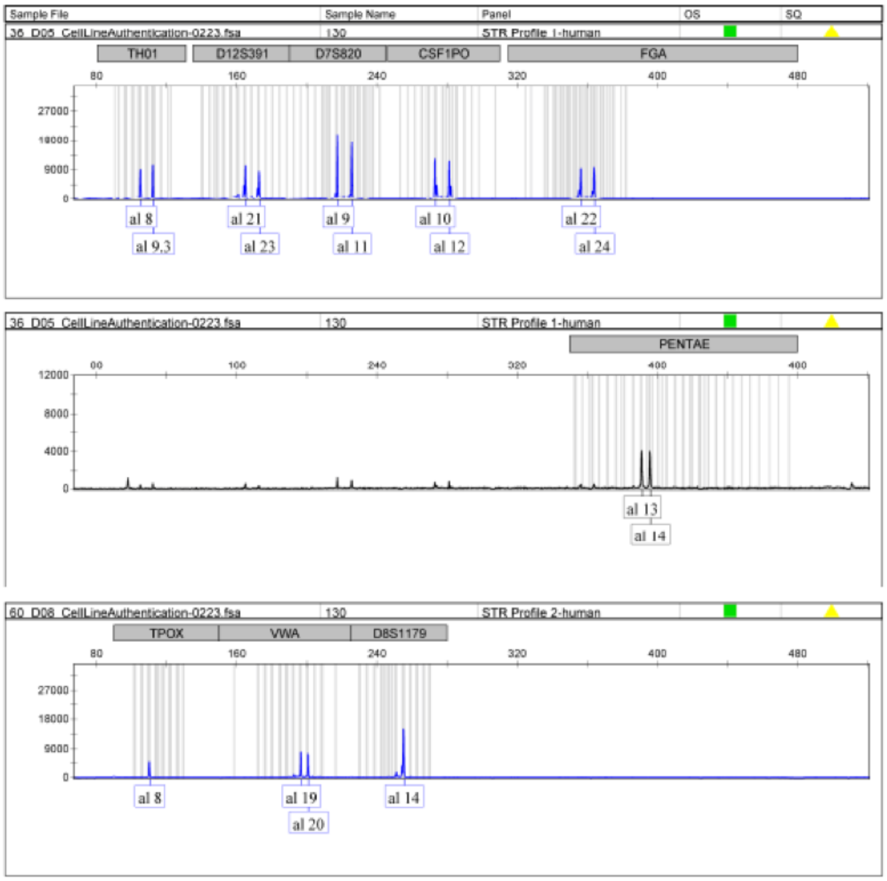

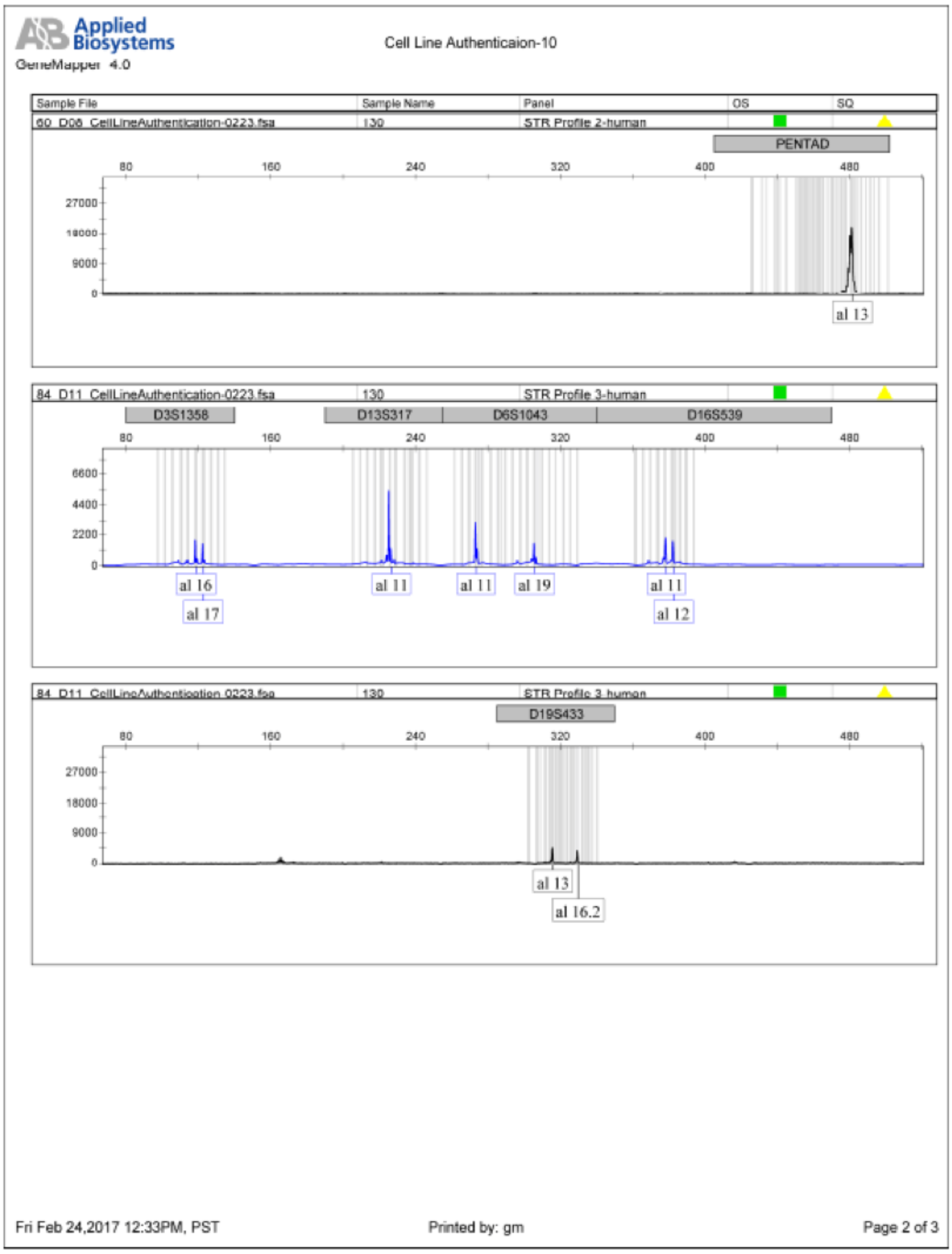
Applied Biosystems
GeneMapper 4.0
Cell Line Authenticaion-10
Sample File
Sample Name
Panel
OS
SQ
60_D08_CellLineAuthentication-0223.fsa
130
STR Profile 2-human
PENTAD
al 13
84_D11_CellLineAuthentication-0223.fsa
130
STR Profile 3-human
D3S1358
D13S317
D6S1043
D16S539
al 16
al 17
al 11
al 11
al 19
al 11
al 12
D19S433
al 13
al 16.2
Fri Feb 24,2017 12:33PM, PST
Printed by: gm
Page 2 of 3

Applied Biosystems

GeneMapper 4.0

Cell Line Authenticaion-10

Sample File	Sample Name	Panel	OS	SQ
12_D02_CellLineAuthentication-0223.fsa	130	STR Profile 4-human		

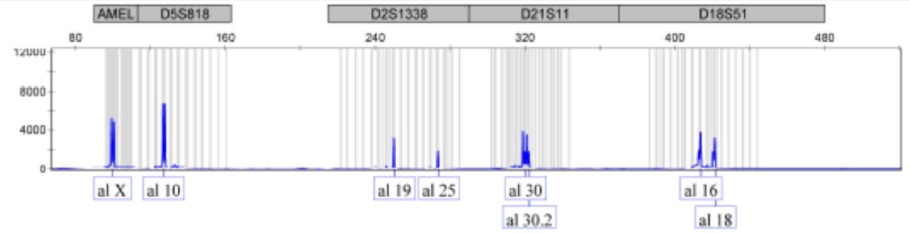

12_D02_CellLineAuthentication-0223.fsa	130	STR Profile 4-human		

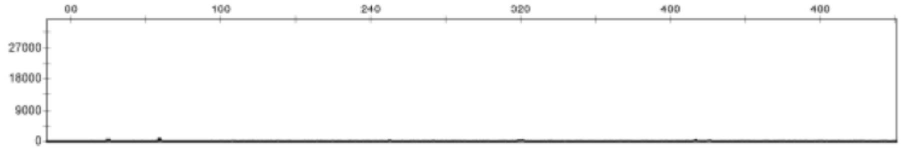

BEAS-2B

Applied Biosystems
GeneMapper 4.0

Cell Line Authentication 0906

Sample File	Sample Name	Panel	OS	SQ
16_H02_CellLineAuthentication-1-0907.fsa	1	STR Profile 1-human		

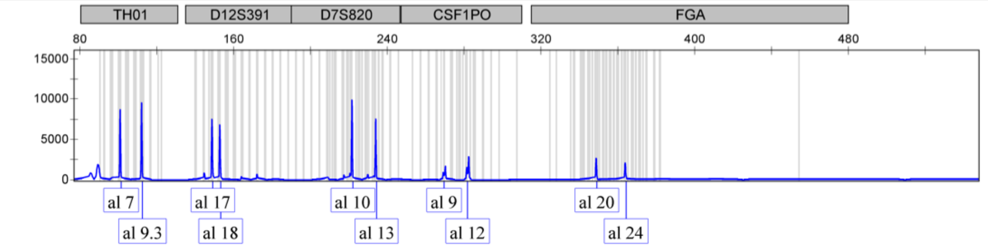

Sample File	Sample Name	Panel	OS	SQ
16_H02_CellLineAuthentication-1-0907.fsa	1	STR Profile 1-human		

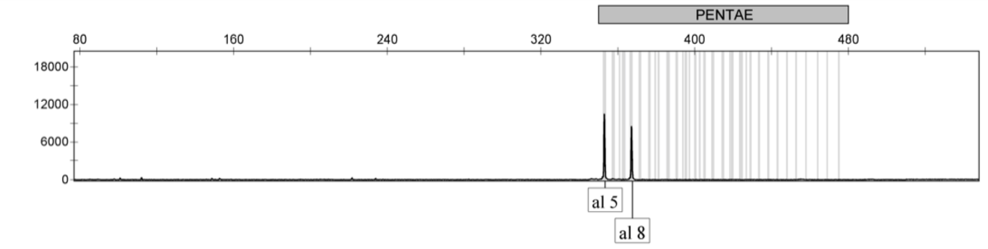

Sample File	Sample Name	Panel	OS	SQ
37_E05_CellLineAuthentication-1-0907.fsa	1	STR Profile 2-human		

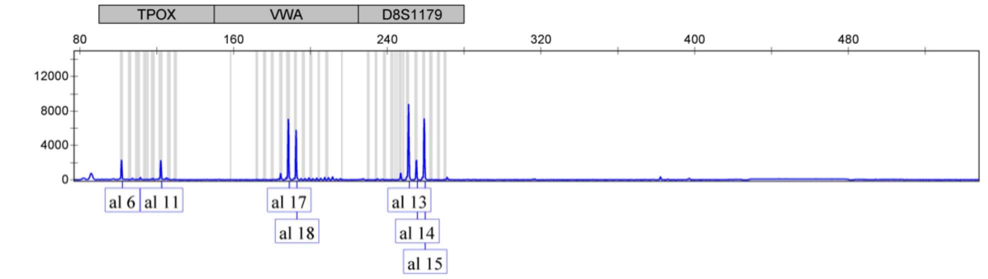

Sample File	Sample Name	Panel	OS	SQ
37_E05_CellLineAuthentication-1-0907.fsa	1	STR Profile 2-human		

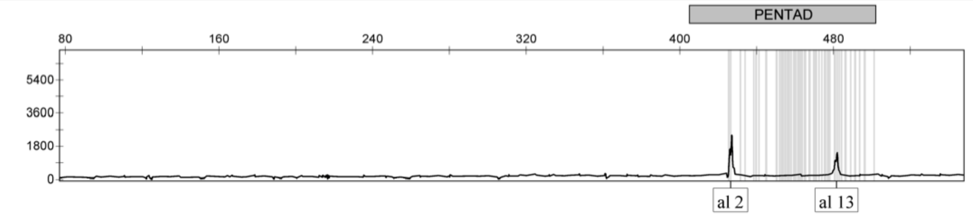

Sample File	Sample Name	Panel	OS	SQ
39_G05_CellLineAuthentication-1-0907.fsa	1	STR Profile 3-human		

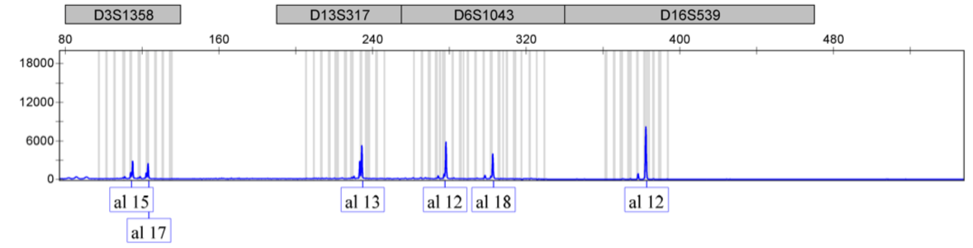

Sample File	Sample Name	Panel	OS	SQ
39_G05_CellLineAuthentication-1-0907.fsa	1	STR Profile 3-human		

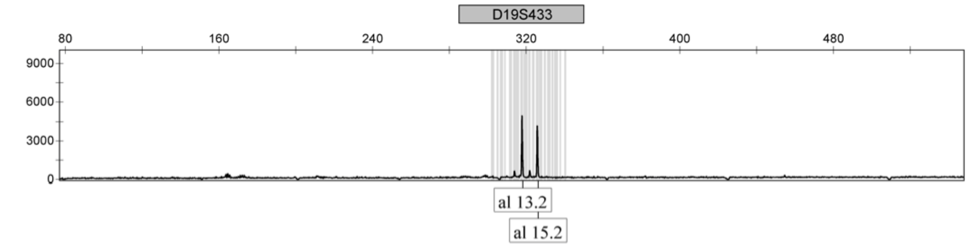

Applied Biosystems

GeneMapper 4.0

Cell Line Authentication 0906

Sample File	Sample Name	Panel	OS	SQ
88_H11_CellLineAuthentication-1-0907.fsa	1	STR Profile 4-human		

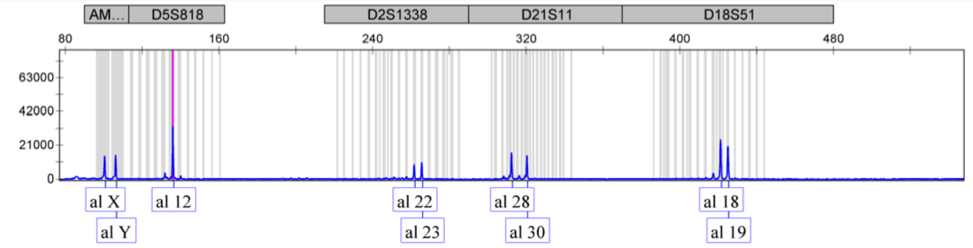

88_H11_CellLineAuthentication-1-0907.fsa	1	STR Profile 4-human		

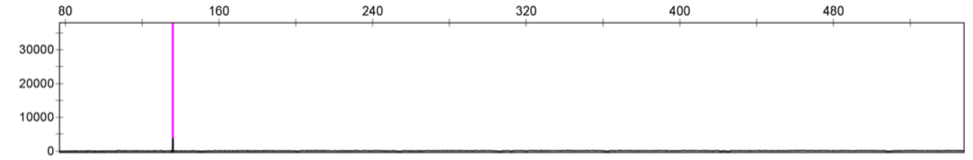

H358

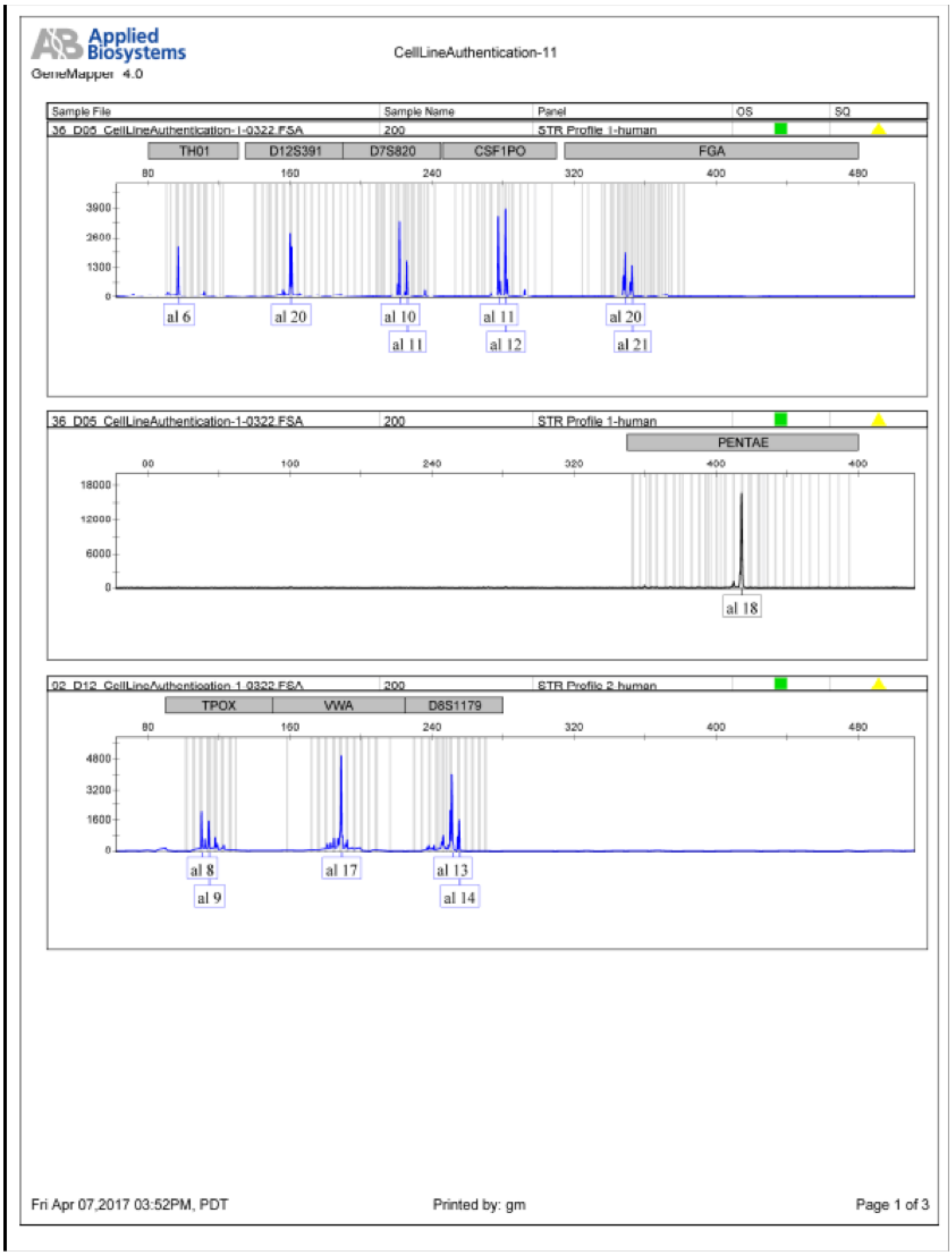
Applied Biosystems
GeneMapper 4.0
CellLineAuthentication-11
Sample File
Sample Name
Panel
OS
SQ
36_D05_CellLineAuthentication-1-0322.FSA
200
STR Profile 1-human
TH01
D12S391
D7S820
CSF1PO
FGA
al 6
al 20
al 10
al 11
al 11
al 12
al 20
al 21
PENTAE
al 18
02_D12_CellLineAuthentication-1-0322.FSA
STR Profile 2-human
TPOX
VWA
D8S1179
al 8
al 9
al 17
al 13
al 14

Applied Biosystems
GeneMapper 4.0

CellLineAuthentication-11

Sample File	Sample Name	Panel	OS	SQ
92_D12_CellLineAuthentication-1-0322.FSA	200	STR Profile 2-human		

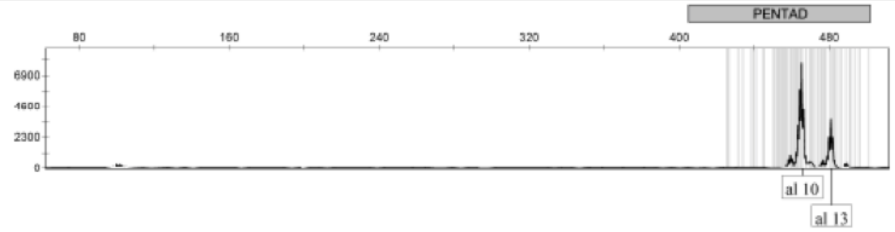

52_D07_CellLineAuthentication-0323.fsa	200	STR Profile 3-human		

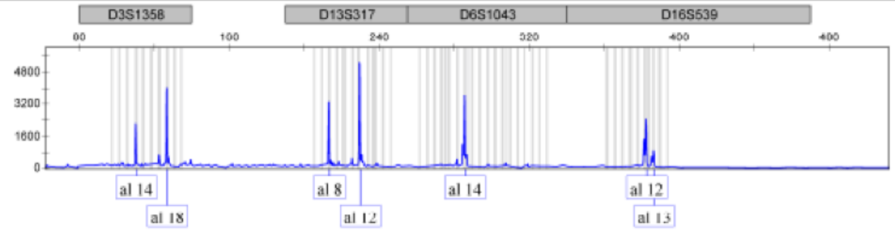

52_D07_CellLineAuthentication-0323.fsa	200	STR Profile 3-human		

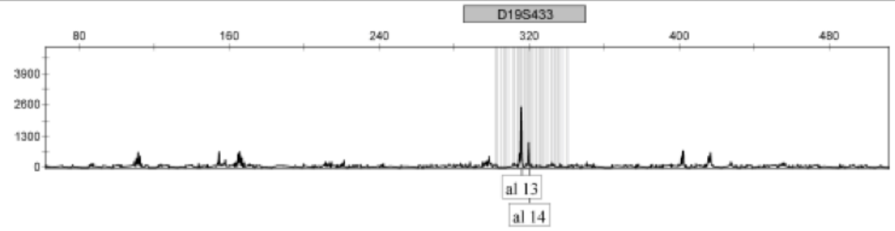

Applied Biosystems
GeneMapper 4.0

CellLineAuthentication-11

Sample File	Sample Name	Panel	OS	SQ
36_D05_XBJD1-9WZC0316(81)(1-3)1012-0324.fsa	200	STR Profile 4-human		

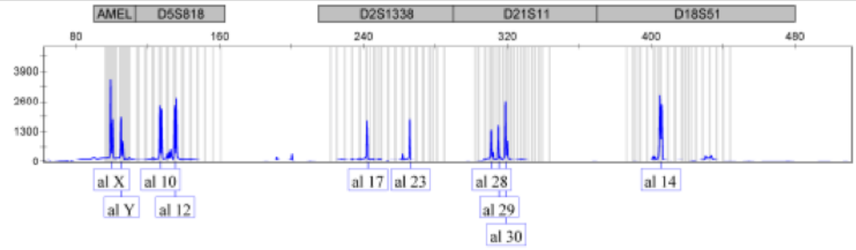

36_D05_XBJD1-9WZC0316(81)(1-3)1012-0324.fsa	200	STR Profile 4-human		

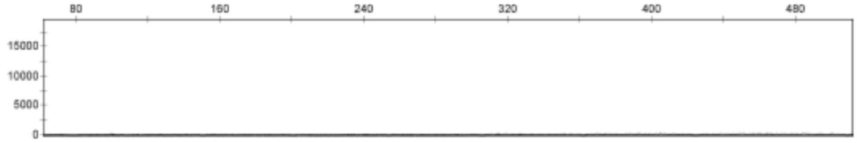

H1650

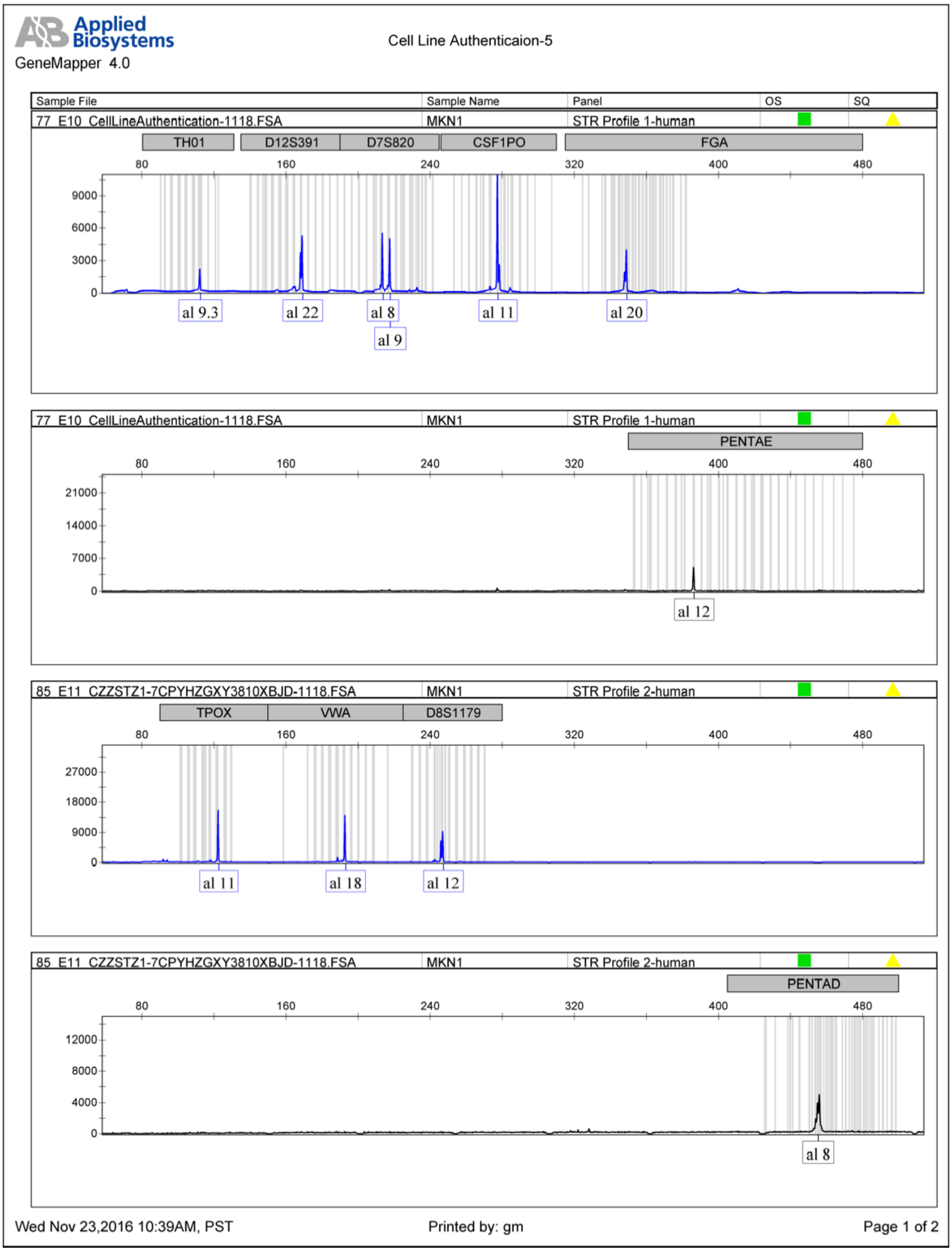

Applied Biosystems
GeneMapper 4.0
Cell Line Authenticaion-5
Sample File
Sample Name
Panel
OS
SQ
77_E10_CellLineAuthentication-1118.FSA
MKN1
STR Profile 1-human
TH01
D12S391
D7S820
CSF1PO
FGA
al 9.3
al 22
al 8
al 9
al 11
al 20
77_E10_CellLineAuthentication-1118.FSA
MKN1
STR Profile 1-human
PENTAE
al 12
85_E11_CZZSTZ1-7CPYHZGXY3810XBJD-1118.FSA
MKN1
STR Profile 2-human
TPOX
VWA
D8S1179
al 11
al 18
al 12
85_E11_CZZSTZ1-7CPYHZGXY3810XBJD-1118.FSA
MKN1
STR Profile 2-human
PENTAD
al 8
Wed Nov 23,2016 10:39AM, PST
Printed by: gm
Page 1 of 2

Applied Biosystems
GeneMapper 4.0

Cell Line Authenticaion-5

Sample File	Sample Name	Panel	OS	SQ
93_E12_CZZSTZ1-7CPYHZGXY3810XBJD-1118.FSA	MKN1	STR Profile 3-human		

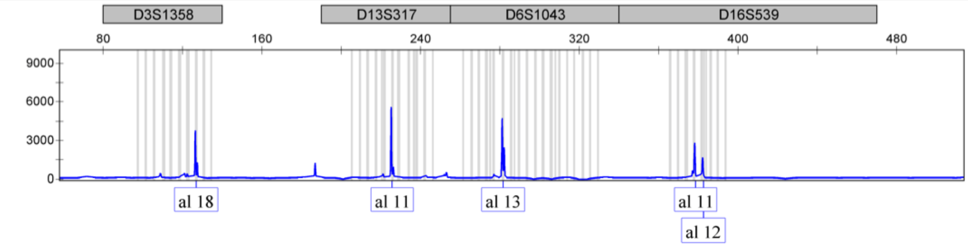

93_E12_CZZSTZ1-7CPYHZGXY3810XBJD-1118.FSA	MKN1	STR Profile 3-human		

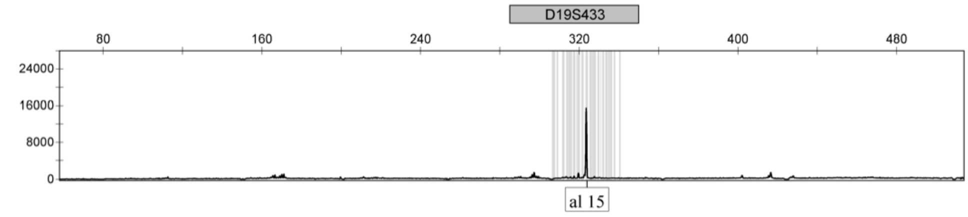

93_E12_CellLineAuthentication-1118.FSA	MKN1	STR Profile 4-human		

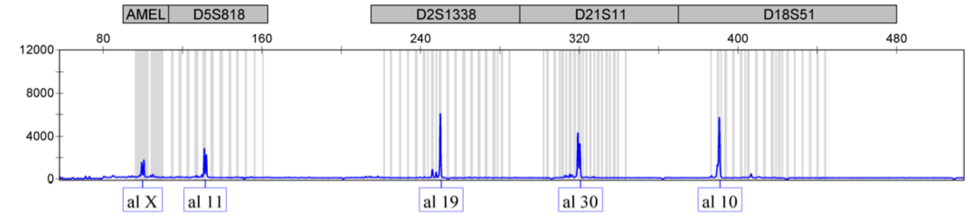

93_E12_CellLineAuthentication-1118.FSA	MKN1	STR Profile 4-human		

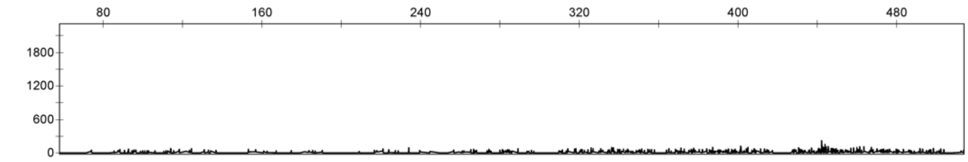

PC9

Applied Biosystems

CellLineAuthentication-0403

GeneMapper 4.0

Sample File	Sample Name	Panel	SQI	OS	SQ
11_C02_CellLineAuthentication-1-0411.fsa	99	STR Profile 1-human			

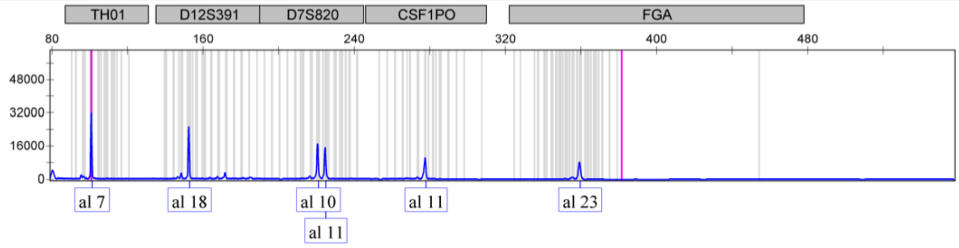

11_C02_CellLineAuthentication-1-0411.fsa	99	STR Profile 1-human			

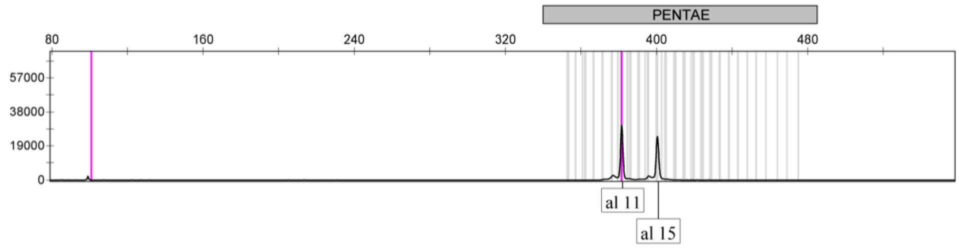

27_C04_CellLineAuthentication-1-0411.fsa	99	STR Profile 2-human			

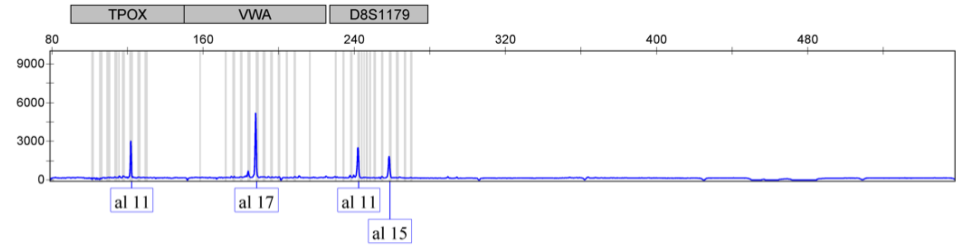

Sample File	Sample Name	Panel	SQI	OS	SQ
27_C04_CellLineAuthentication-1-0411.fsa	99	STR Profile 2-human			

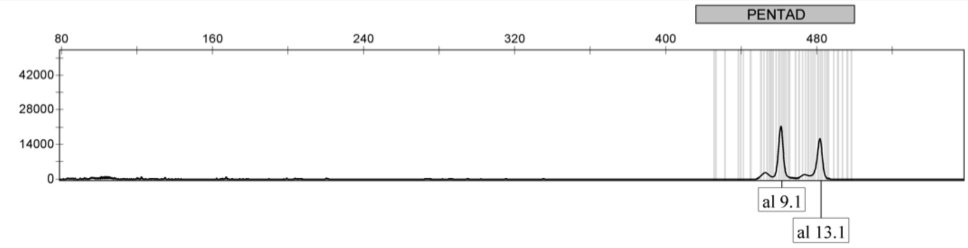

Sample File	Sample Name	Panel	SQI	OS	SQ
43_C06_CellLineAuthentication-1-0411.fsa	99	STR Profile 3-human			

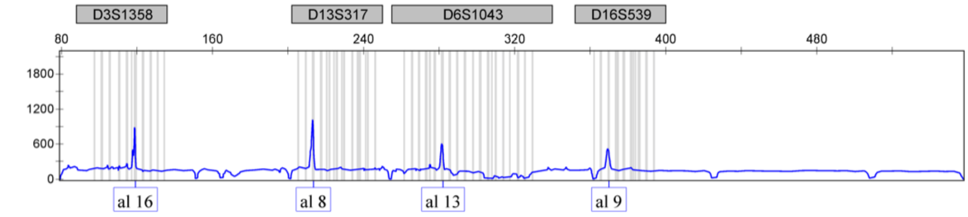

Sample File	Sample Name	Panel	SQI	OS	SQ
43_C06_CellLineAuthentication-1-0411.fsa	99	STR Profile 3-human			

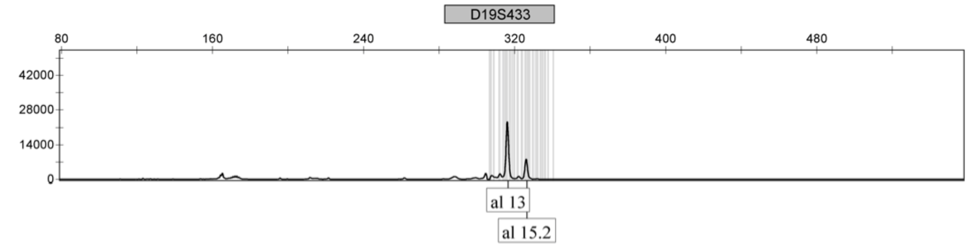

Sample File	Sample Name	Panel	SQI	OS	SQ
59_C08_CellLineAuthentication-1-0411.fsa	99	STR Profile 4-human			

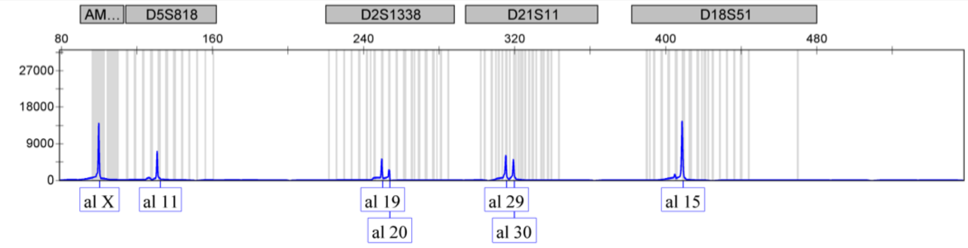

59_C08_CellLineAuthentication-1-0411.fsa	99	STR Profile 4-human			

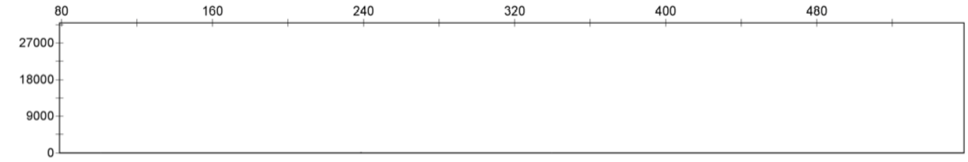

H1975

Applied Biosystems

CELL-13

GeneMapper 4.0

Sample File	Sample Name	Panel	OS	SQ
01_A01_XiBaoJianDing1-10WZC0419-0424.fsa	H2030	STR Profile 1-human		

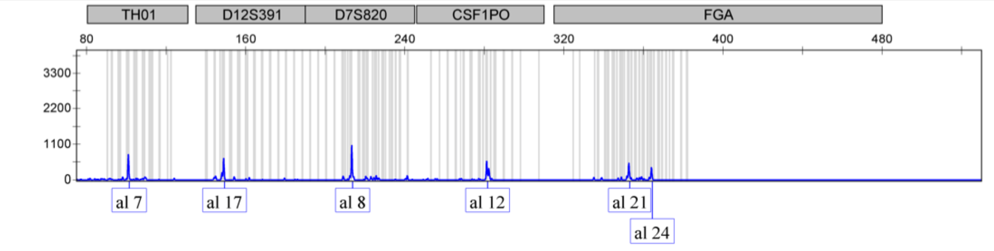

Sample File	Sample Name	Panel	OS	SQ
01_A01_XiBaoJianDing1-10WZC0419-0424.fsa	H2030	STR Profile 1-human		

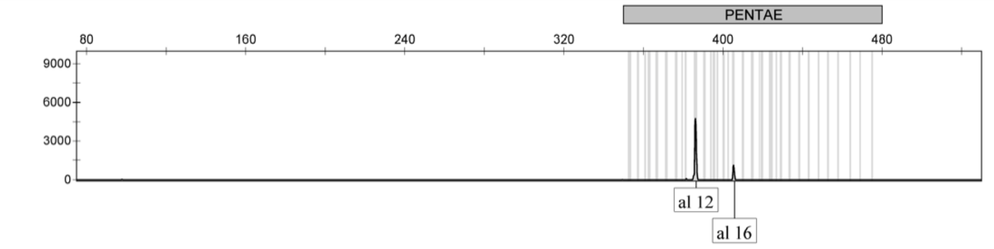

Sample File	Sample Name	Panel	OS	SQ
25_A04_XiBaoJianDing1-10WZC0419-0424.fsa	H2030	STR Profile 2-human		

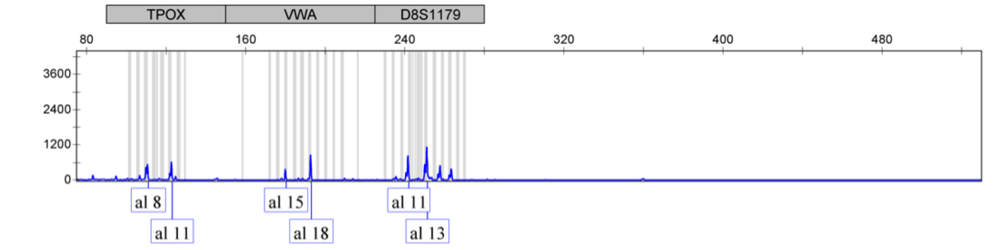

Sample File	Sample Name	Panel	OS	SQ
25_A04_XiBaoJianDing1-10WZC0419-0424.fsa	H2030	STR Profile 2-human		

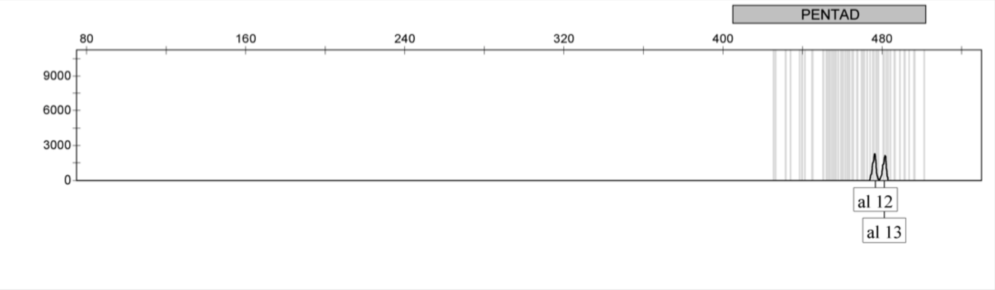

Sample File	Sample Name	Panel	OS	SQ
41_A06_XiBaoJianDing1-10WZC0419-0424.fsa	H2030	STR Profile 3-human		

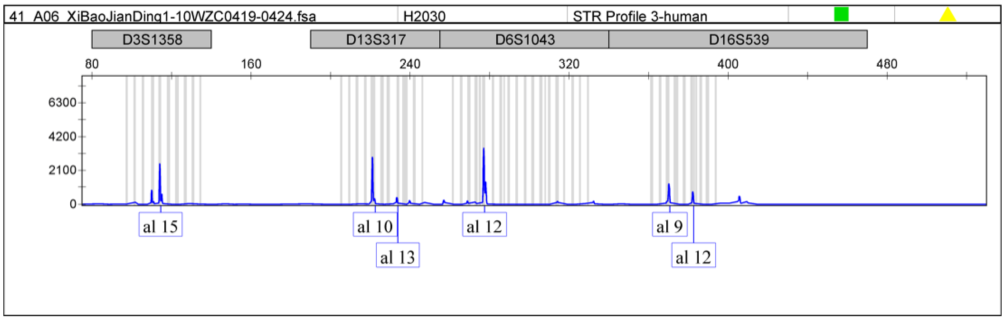

Sample File	Sample Name	Panel	OS	SQ
41_A06_XiBaoJianDing1-10WZC0419-0424.fsa	H2030	STR Profile 3-human		

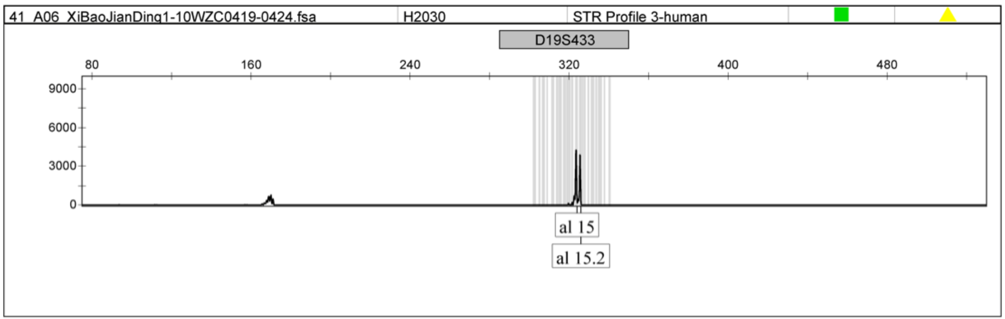

GeneMapper 4.0

CELL-13

Sample File	Sample Name	Panel	OS	SQ
65_A09_XiBaoJianDing1-10WZC0419-0424.fsa	H2030	STR Profile 4-human		

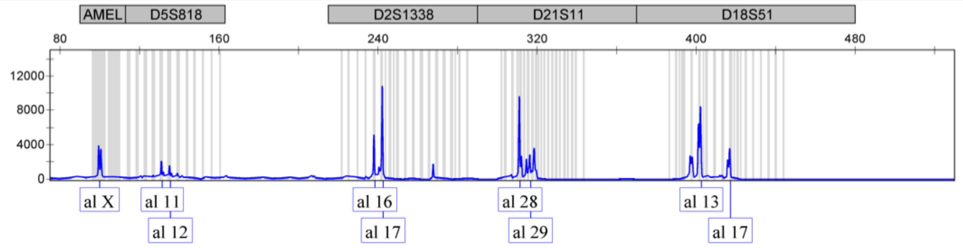

65_A09_XiBaoJianDing1-10WZC0419-0424.fsa	H2030	STR Profile 4-human		

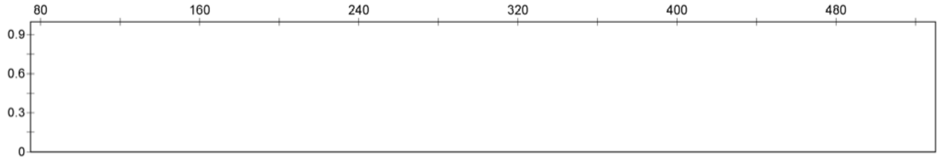

A549

Applied Biosystems
GeneMapper 4.0

Cell Line Authentication-2

Sample File	Sample Name	Panel	OS	SQ
65_A09_CellLineAuthentication09105-0919.FSA	035	STR Profile 1-dup		

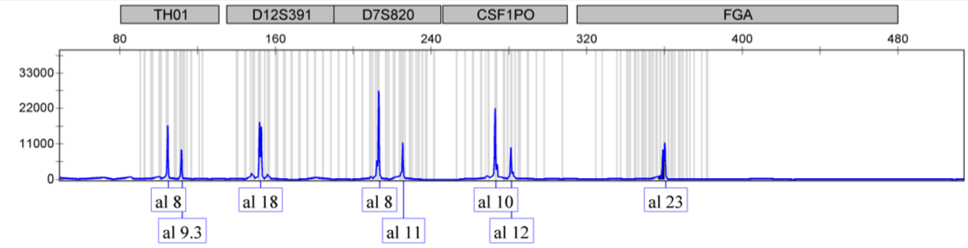

Sample File	Sample Name	Panel	OS	SQ
01_A01_CellLineAuthentication09122-0919.FSA	035	STR Profile 2-dup		

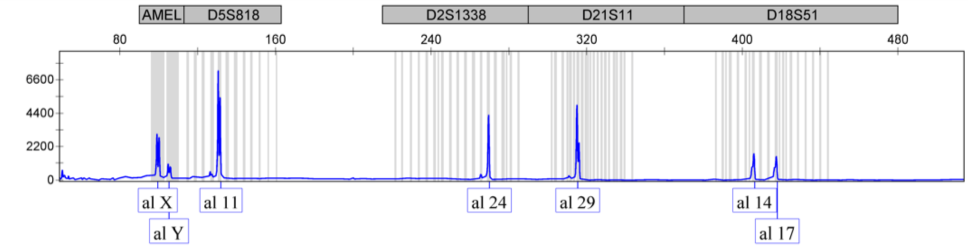

Sample File	Sample Name	Panel	OS	SQ
33_A05_CellLineAuthentication09122-0919.FSA	035	STR Profile 3-dup		

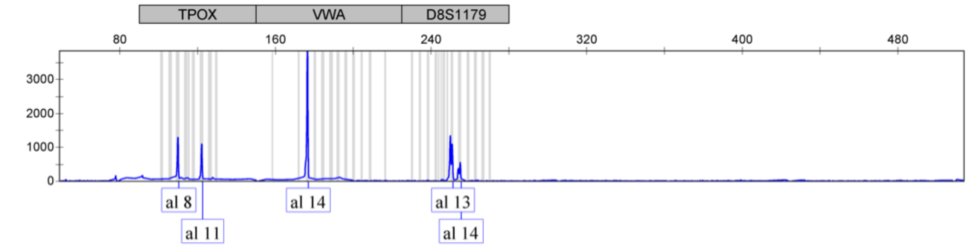

GeneMapper 4.0

Cell Line Authentication-2

Sample File	Sample Name	Panel	OS	SQ
65_A09_CellLineAuthentication09122-0919.FSA	035	STR Profile 4-dup		

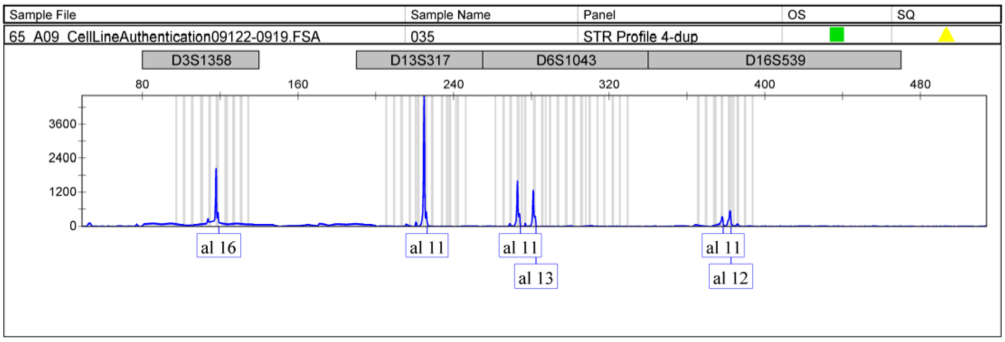

Sample File	Sample Name	Panel	OS	SQ
01_A01_CellLineAuthentication09125-0919.fsa	035	STR Profile 5-dup		

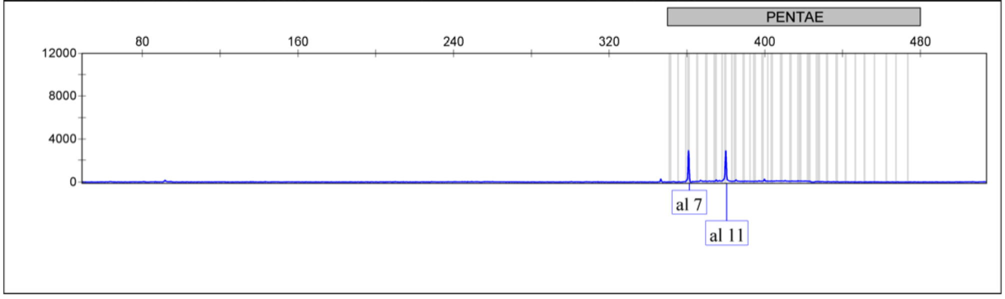

Sample File	Sample Name	Panel	OS	SQ
33_A05_CellLineAuthentication09125-0919.fsa	035	STR Profile 6-dup		

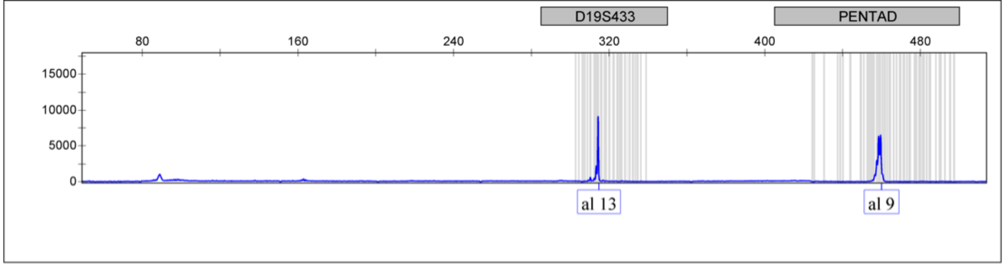

H1299

Applied Biosystems

GeneMapper 4.0

14

Sample File	Sample Name	Panel	OS	SQ
39_G05_CellLineAuthentication-0519.FSA	2	STR Profile 1-human		

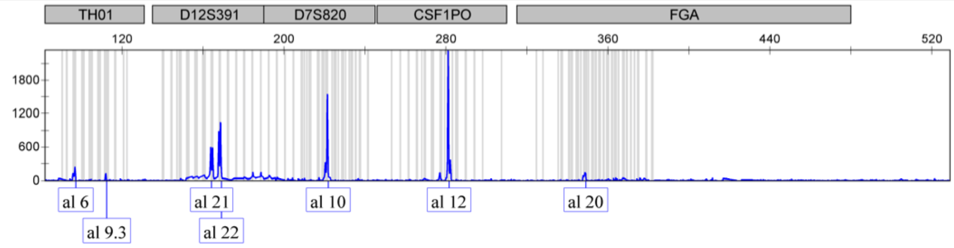

39_G05_CellLineAuthentication-0519.FSA	2	STR Profile 1-human		

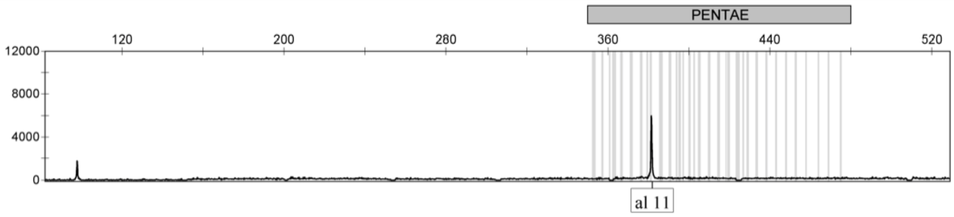

63_G08_CellLineAuthentication-0519.FSA	2	STR Profile 2-human		

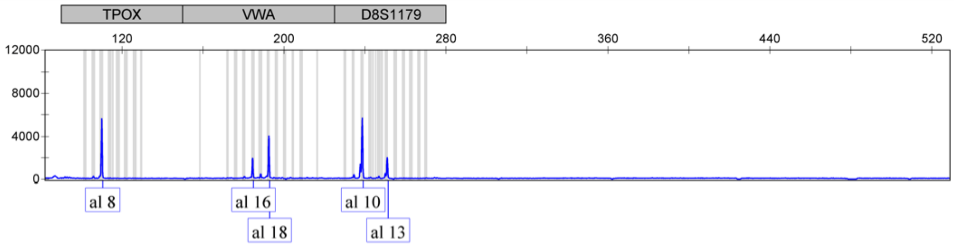

Sample File	Sample Name	Panel	OS	SQ
63_G08_CellLineAuthentication-0519.FSA	2	STR Profile 2-human		

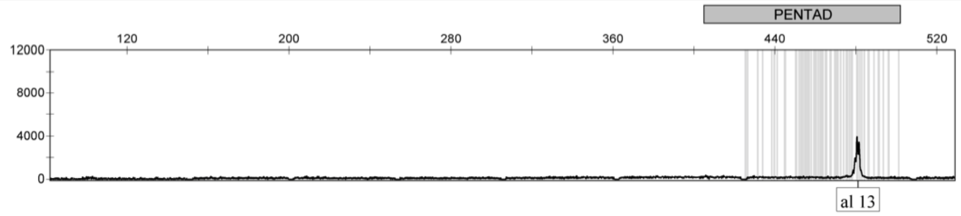

Sample File	Sample Name	Panel	OS	SQ
23_G03_CellLineAuthentication-0523.fsa	2	STR Profile 3-human		

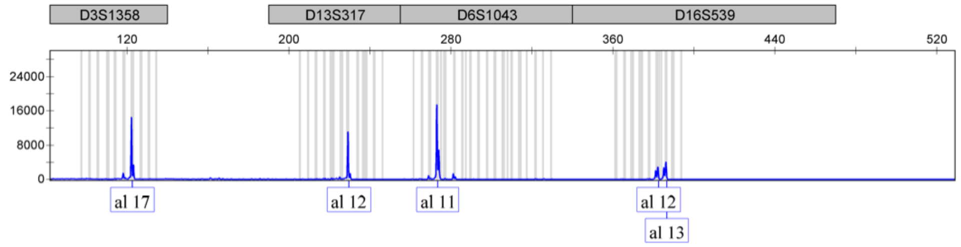

Sample File	Sample Name	Panel	OS	SQ
23_G03_CellLineAuthentication-0523.fsa	2	STR Profile 3-human		

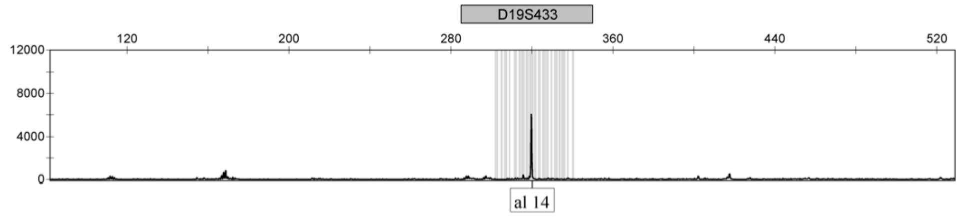

Sample File	Sample Name	Panel	OS	SQ
49_A07_CellLineAuthentication-0523.fsa	2	STR Profile 4-human		

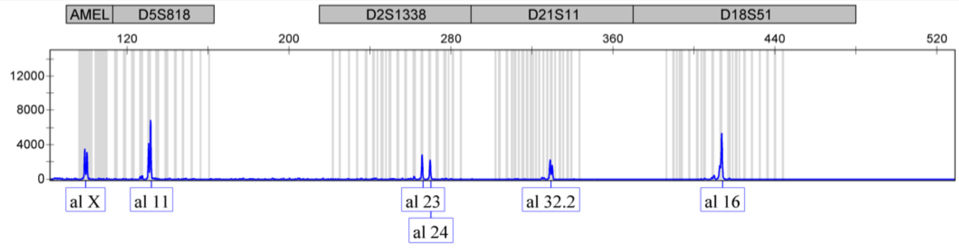

49_A07_CellLineAuthentication-0523.fsa	2	STR Profile 4-human		

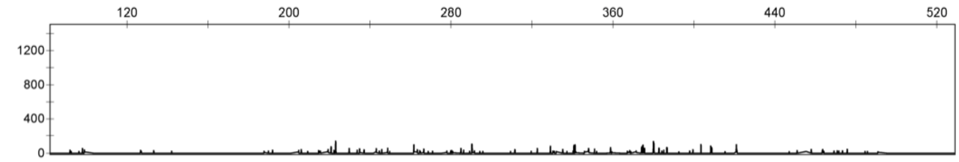

HCC827

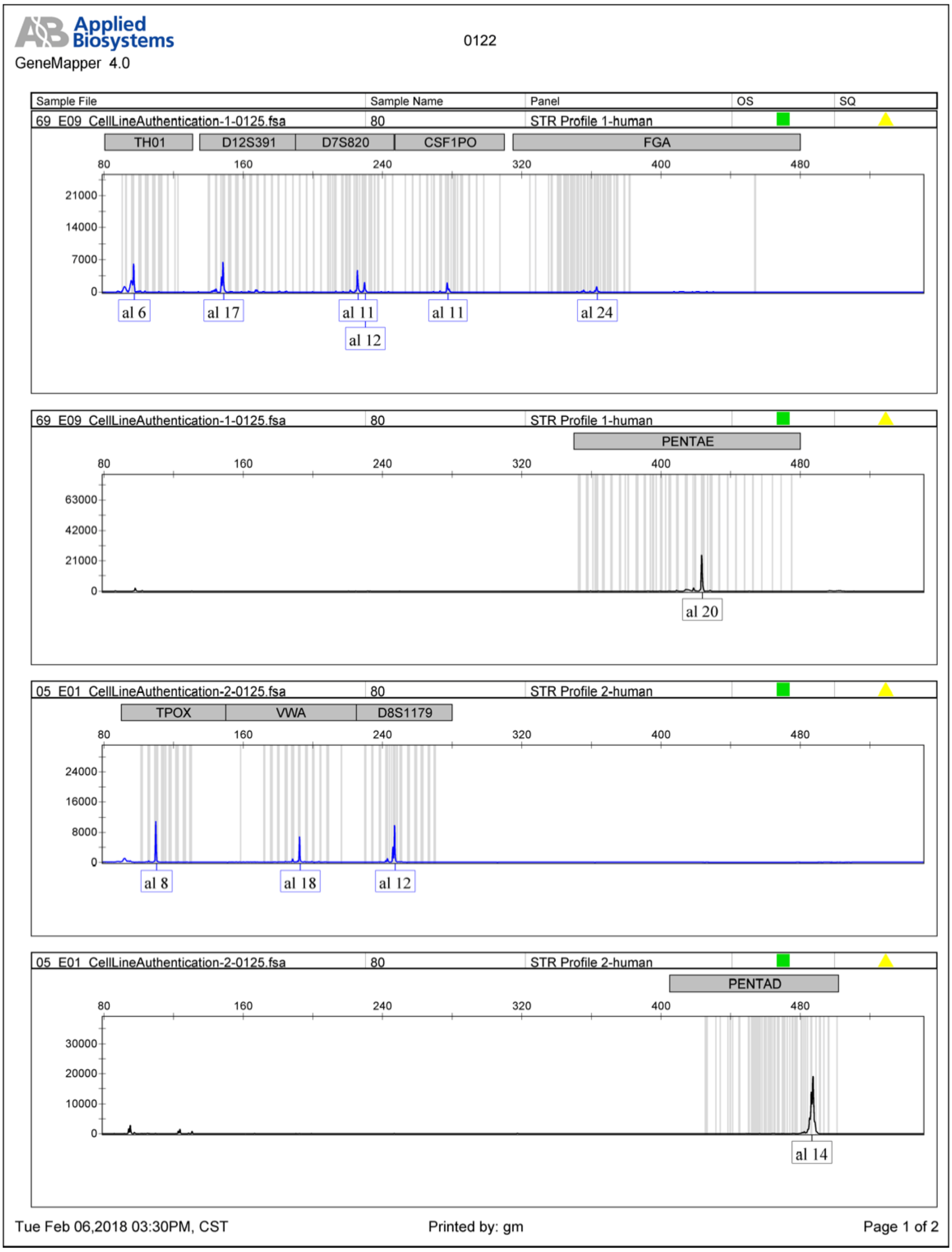
Applied Biosystems
GeneMapper 4.0
0122
Sample File
Sample Name
Panel
OS
SQ
69_E09_CellLineAuthentication-1-0125.fsa
80
STR Profile 1-human
TH01
D12S391
D7S820
CSF1PO
FGA
al 6
al 17
al 11
al 12
al 11
al 24
PENTAE
al 20
05_E01_CellLineAuthentication-2-0125.fsa
STR Profile 2-human
TPOX
VWA
D8S1179
al 8
al 18
al 12
PENTAD
al 14
Tue Feb 06,2018 03:30PM, CST
Printed by: gm
Page 1 of 2

Applied Biosystems

GeneMapper 4.0

0122

Sample File	Sample Name	Panel	OS	SQ
37_E05_CellLineAuthentication-2-0125.fsa	80	STR Profile 3-human		

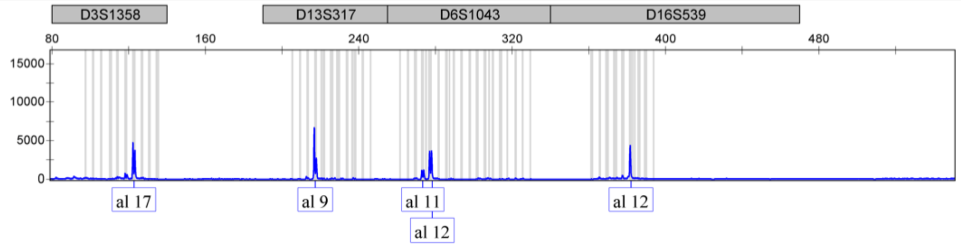

Sample File	Sample Name	Panel	OS	SQ
37_E05_CellLineAuthentication-2-0125.fsa	80	STR Profile 3-human		

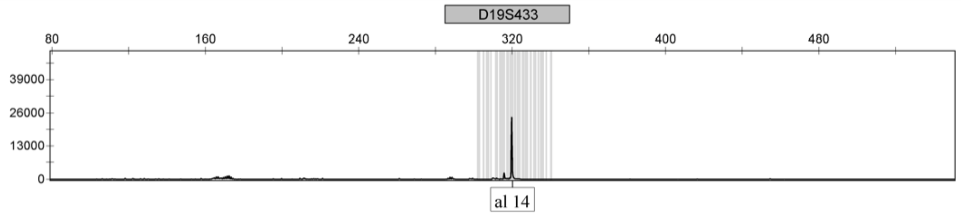

Sample File	Sample Name	Panel	OS	SQ
69_E09_CellLineAuthentication-2-0125.fsa	80	STR Profile 4-human		

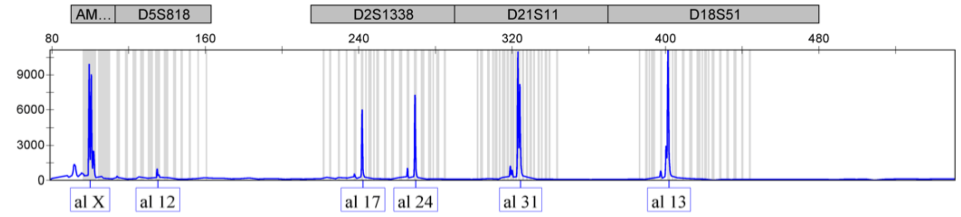

Sample File	Sample Name	Panel	OS	SQ
69_E09_CellLineAuthentication-2-0125.fsa	80	STR Profile 4-human		

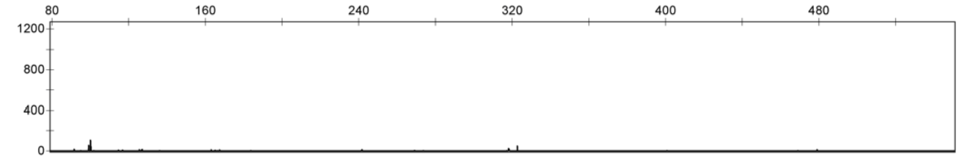

Supplement: Supplementary file 1 — Supplementary figures and tables. [file thnov11p5650s1.pdf]
